# Enantioselective Beckmann Rearrangement through Organocatalytic Desymmetrization of Cyclobutanones for the Synthesis of 4,4-Disubstituted γ‑Lactams

**DOI:** 10.1021/acs.orglett.5c04735

**Published:** 2025-12-23

**Authors:** Antón Igartua, Liher Prieto, Uxue Uria, Luisa Carrillo, Efraim Reyes, Jose L. Vicario

**Affiliations:** Department of Organic and Inorganic Chemistry, University of the Basque Country (EHU), P.O. Box 644, 48080 Bilbao, Spain

## Abstract

The Beckmann rearrangement stands as a powerful and atom economic transformation, and its enantioselective version has not yet been found. This work presents the organocatalytic enantioselective desymmetric synthesis of γ-lactams from prochiral 3,3-disubstituted cyclobutanones through the *in situ* formation of reactive *N*-sulfonyl oximes followed by Beckmann rearrangement. The reaction relies on the stereocontrolled generation of the axially chiral cyclobutanone oxime sulfonate intermediate under chiral Brønsted acid catalysis and subsequent axial-to-central chirality transfer.

## Graphical abstract

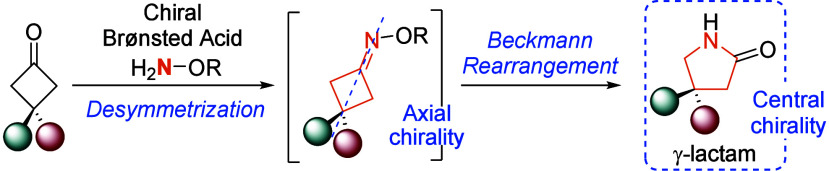

The Beckmann rearrangement can be regarded as a key synthetic tool in organic chemistry for the formation of amides, enabling the insertion of a nitrogen atom on both cyclic and acyclic oximes.^1^ It is noteworthy that Beckmann rearrangement represents a highly useful disconnection strategy in the design of complex molecules, demonstrating its applicability to the production of several key intermediates that are involved in the synthesis of fine and bulk chemicals.^2^ Despite all of the advances in the field that have widened the scope of this powerful reaction, its application to the generation of stereodefined chiral amides still has important limitations. Indeed, all examples reported in the literature involve the use of chiral enantioenriched starting materials, therefore relying on diastereoselective and/or enantiospecific versions of the Beckmann rearrangement (for an elegant example of catalytic desymmetrization to form a diastereopure chiral oxime followed by stereoretentive Beckmann rearrangement, see Scheme 1A).^3^ This observation finds its explanation in the stereospecificity associated with the migration process in the Beckmann rearrangement, where the migrating unit is the one placed *anti* to the N–O bond in the oxime derivative.

**Scheme 1:**
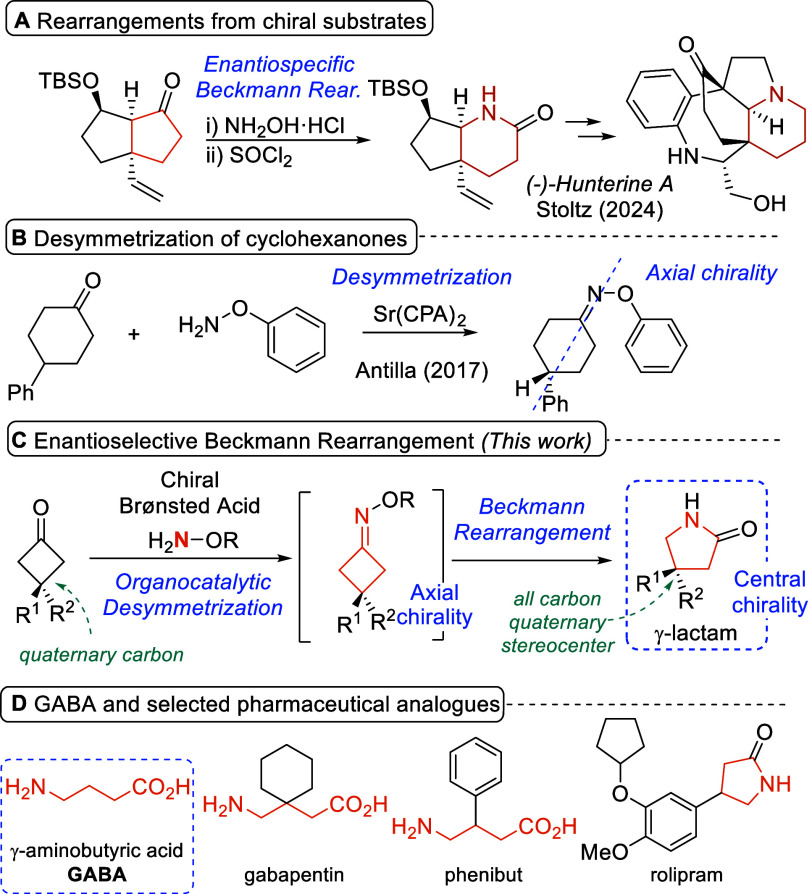
Advances in the Development of Stereoselective Beckmann Rearrangements

With the aim of facing this inherent mechanistic challenge, we envisaged the possibility of applying a desymmetrization strategy.^4^ Some literature precedents have demonstrated the potential use of asymmetric catalysis for the desymmetrization of cyclic ketones through the enantioselective formation of axially chiral cycloalkenone-derived oxime ethers.^5^ Specifically, Antilla pioneered the enantioselective synthesis of cyclohexanone oximes using a Sr­(II)-based chiral Lewis acid catalyst (Scheme 1B).^6^ This work was followed by reports outlining the possibility of using chiral Brønsted acid catalysis to trigger analogous desymmetrization transformation from prochiral cyclopentanones and cycloheptanones to form structurally related axially chiral oximes.^7^ Despite all of these advances, it should be highlighted that none of these precedents have been demonstrated to engage axially chiral oximes directly in a subsequent Beckmann rearrangement process.^8^

Our reaction design relies on the ability of activated hydroxylamine ether derivatives to undergo chiral Brønsted acid-catalyzed enantioselective condensation with the 3,3-disubstituted cyclobutanone starting material, generating an axially chiral oxime with the ability to undergo Beckmann rearrangement (Scheme 1C). The strain release associated with the conversion from the cyclobutane to the five-membered cyclic scaffold would contribute to overcoming the kinetic inertness of oximes toward the Beckmann rearrangement.^9^ Remarkably, this desymmetrization strategy can be considered as a particularly useful approach to generate otherwise challenging quaternary stereocenters enantioselectively, as the sterically congested quaternary carbon atom can be easily installed through a conventional high-yielding methodology during the preparation of the starting material. Furthermore, the methodology points to the formation of enantioenriched γ-lactams, compounds of interest that are present as core scaffolds of a variety of compounds with relevant biological activities such as substituted γ-aminobutyric acid (GABA) neurotransmitter modulators (Scheme 1D).^10^

We started with the evaluation of the reactivity of 3-methyl-3-phenylcyclobutanone **1a** as a readily available model substrate. In this initial phase, we proceeded to evaluate different aminating agents (**2a**–**d**) in the presence of stoichiometric amounts of a Brønsted acid to check the viability of the projected acid-promoted cascade condensation/Beckmann rearrangement process (Scheme 2).^11^ *O*-(2,4-Dinitrophenyl) hydroxylamine **2a** provided oxime ether **4a** in good yield, but this intermediate was unable to evolve into ring-expansion product **3a** even after prolonged refluxing in toluene. *O-*(Diphenylphosphinyl)­hydroxylamine **2b** behaved similarly, despite the reported ability of the diphenylphosphinate group to act as an excellent leaving group in related aza-Baeyer–Villiger reactions.^12^ On the other hand, *O*-sulfonylhydroxylamine-derived reagents **2c** and **2d** delivered the desired Beckmann rearrangement product **3a** in good yields. Of these, reagent **2d** gave superior results in terms of the isolated yield of **3a**, safety, handling, and stability criteria.^13^

**Scheme 2:**
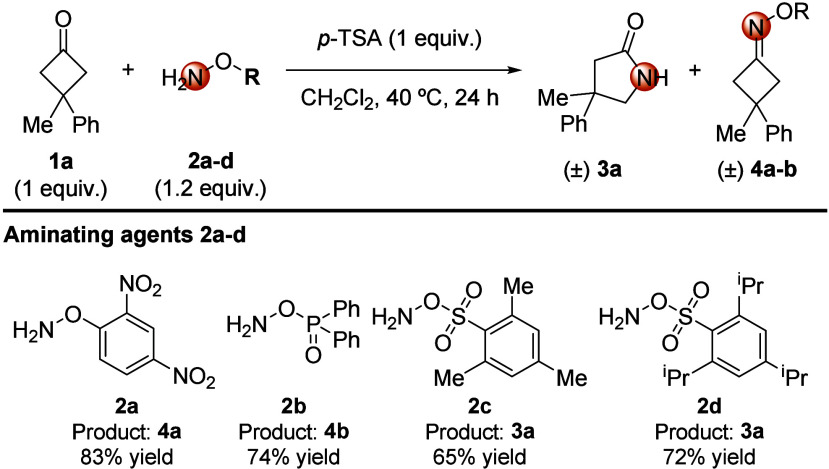
Evaluation of Aminating Agents as Rearrangement Promoters

Once suitable *O*-sulfonyl hydroxylamine **2d** had been identified, the corresponding catalytic enantioselective version of the transformation was surveyed, employing BINOL-derived chiral Brønsted acids as catalysts. The use of 3,3′-bis­(2,4,6-triisopropylphenyl)-substituted phosphoric acid **5a** in CH_2_Cl_2_ at room temperature provided a moderate yield of **3a** in a racemic form (Table 1, entry 1). *N*-Triflyl phosphoramide analogue **5b** with increased acidity^14^ at room temperature delivered the desired γ-lactam in a satisfactory yield, albeit with no enantiocontrol (Table 1, entry 2), proving the efficiency of this type of catalyst to promote the reaction. A decrease in the reaction temperature resulted in improved enantioselection with no negative impact on the yield (Table 1, entry 3). After full consumption of cyclobutanone **1a**, Na_2_CO_3_ was added to prevent acid-catalyzed racemization of the oxime sulfonate before heating to 60 °C for the Beckmann rearrangement (see Scheme 4). Other 3,3′-substituted derivatives of the catalyst were surveyed, such as π-extended antracen-9-yl **5c** and 3,5-bis­(triphenylsilyl)­phenyl **5d**, which resulted in inferior optical purity of the final product (Table 1, entries 4 and 5, respectively). Other structurally related catalysts bearing bulkier substituents such as 2,4,6-tricyclopentylphenyl-substituted triflyl phosphoramide **5e** were assessed, which albeit providing a similar enantiomeric ratio gave no better results (Table 1, entry 6). With these results, **5b** was selected to survey the effect of the solvent, including other halogenated solvents, such as chloroform and 1,2-dichloroethane (DCE). Both gave comparable enantioselectivities, although the latter gave a slightly higher enantiomeric ratio (Table 1, entries 7 and 8, respectively). Other solvents were evaluated, including aromatic solvents such as toluene (Table 1, entry 9), providing inferior enantiocontrol. Considering the efficiency of the reaction in DCE at −35 °C, we decided to employ as the reaction solvent a 1:1 CH_2_Cl_2_/DCE mixture, which could be cooled to −78 °C, achieving the highest enantiomeric ratio of 91:9 for this reaction (Table 1, entry 10). 2,4,6-Triisopropylphenyl-substituted SPINOL triflamide **5f**, albeit in satisfactory yields, failed to provide improved enantiocontrol (Table 1, entry 11).

**Table 1:**
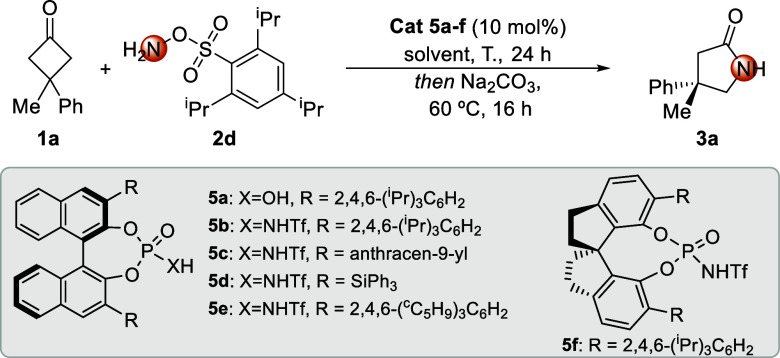
Screening of Reaction Conditions^a^

entry	catalyst	solvent	*T* (°C)	yield (%)^b^	er^c^
1	**5a**	CH_2_Cl_2_	23	55	50:50
2	**5b**	CH_2_Cl_2_	23	75	50:50
3	**5b**	CH_2_Cl_2_	–78	71	86.5:13.5
4	**5c**	CH_2_Cl_2_	–78	70	68:32
5	**5d**	CH_2_Cl_2_	–78	20	54.5:45.5
6	**5e**	CH_2_Cl_2_	–78	51	84:16
7	**5b**	CHCl_3_	–78	62	83:17
8	**5b**	DCE	–35	56	86:14
9	**5b**	toluene	–78	53	76:24
10^d^	**5b**	CH_2_Cl_2_/DCE	–78	86	91.5:8.5
11^d^	**5f**	CH_2_Cl_2_/DCE	–78	83	80:20

a Reaction conditions: **1a** (0.1 mmol), **2d** (0.12 mmol), and **5a**–**f** (0.01 mmol) in 0.5 mL of solvent at the mentioned temperature for 24 h, followed by the addition of Na_2_CO_3_ (0.3 mmol) and heating to 60 °C for 16 h in a sealed tube. DCE = 1,2-dichloroethane.

b Determined after chromatographic purification.

c Determined by chiral HPLC analysis (see the Supporting Information for details).

d A 1:1 CH_2_Cl_2_/DCE mixture was used as the solvent.

With the reaction conditions established, the scope of the transformation was surveyed regarding different cyclobutanes (Table 2). For the study of the electronic effects on the aromatic substituent on C-3 of the starting cyclobutanone, electron-donating groups, namely, methyl and methoxy, were tolerated at different positions of the phenyl ring (**3b**–**f**), achieving the formation of the product even for more sterically congested *o*-methyl-substituted **3f**. The absolute configuration of compound **3e** was established by X-ray analysis. Cyclobutanones with aryl-containing electron-withdrawing substituents, such as F, Cl, and Br, provided γ-lactam products **3g**–**i**, respectively, in good yields and high stereoselectivity. Extended aromatic systems as C-3 substituents were evaluated, among which an enantiomeric ratio of 93:7 was reached with biphenyl substituent **3j**, while 1- and 2-naphthyl rendered rearrangement products **3k** and **3l**, respectively, with moderate er values. The requirement of a quaternary carbon to achieve a high level of enantiocontrol was illustrated with 3-monosubstituted cyclobutanone **1m**, which gave poor enantiocontrol, although in good yield (**3m**). Along the same lines, the influence of replacing the methyl substituent with other alkyl groups was evaluated, which showed that while for ethyl and benzyl substituents the reaction maintained satisfactory levels of enantioselectivity (**3n** and **3o**), the enantiomeric ratio was diminished for the bulkier isopropyl group (**3p**).

**Table 2:**
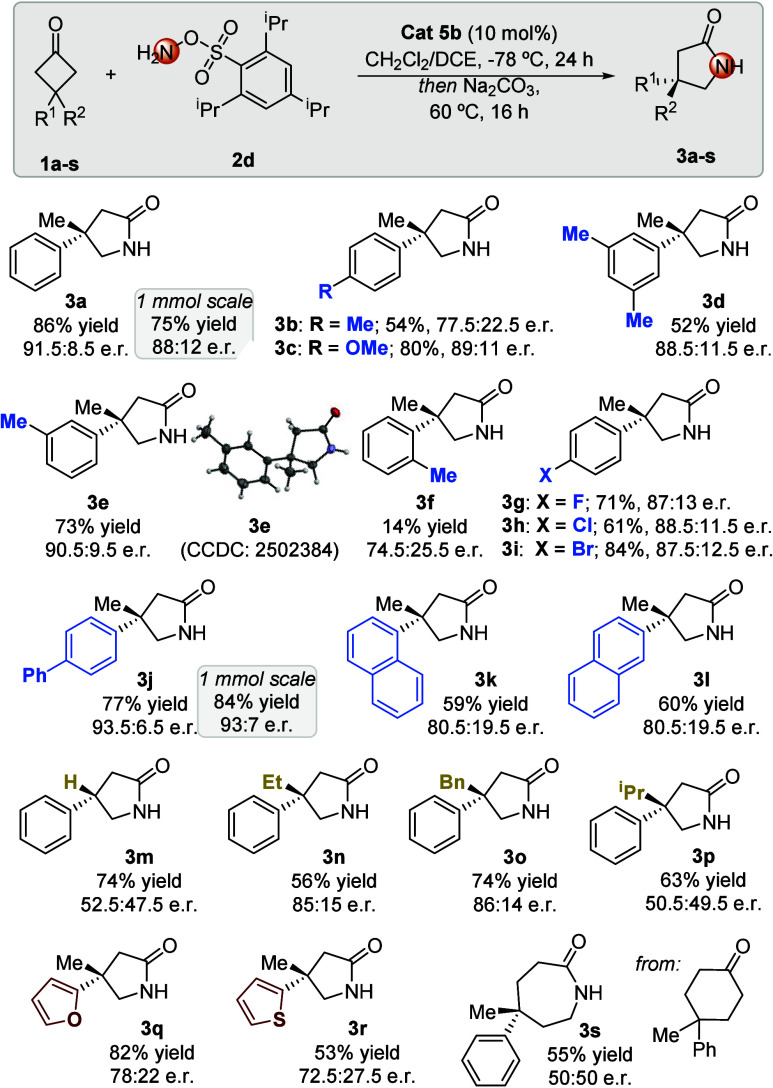
Scope of the Reaction^a^

a Reaction conditions: **1a**–**s** (0.1 mmol), **2d** (0.12 mmol), and **5b** (0.01 mmol), in 0.5 mL of a 1:1 CH_2_Cl_2_/DCE mixture at −78 °C for 24 h, followed by the addition of Na_2_CO_3_ (0.3 mmol) and heating to 60 °C for 16 h in a sealed tube. DCE = 1,2-dichloroethane. The yield was determined after chromatographic purification. The er was determined by chiral HPLC analysis (see the Supporting Information for details).

The possibility of performing the reaction with heteroaromatic groups was exemplified with furan-2-yl and thien-2-yl substituents with acceptable yields and moderate enantiomeric ratios (**3q** and **3r**, respectively). Attempts to perform the reaction employing 4,4-disubstituted cyclohexanones as starting materials resulted in racemic ε-lactams (**3s**). Trials to scale the reaction to 1 mmol were successful, maintaining satisfactory levels of yield and enantioselectivity.

The potential usefulness of this methodology was evaluated by subjecting γ-lactam products **3a** and **3j** to various transformations. The acid hydrolysis of compound **3a** afforded γ-amino acid **6a** as the chlorhydrate salt in high yield, presenting a quaternary stereocenter at C-3, as a phenibut analogue. The reduction of the amide functionality employing LiAlH_4_ followed by *N*-protection as carbamate provided a successful protocol to access pyrrolidine **7a** (Scheme 3A). Lactam **3j** reacted efficiently with benzyl bromide, yielding *N*-benzyl γ-lactam **8j**. Lactam **8j** was subjected to α-alkylation *via* enolate formation to yield product **9j**, which presents an additional stereogenic center, in high yield, as a mixture of diastereomers, with a negligible loss of optical purity for both diastereomers (Scheme 3B).

**Scheme 3:**
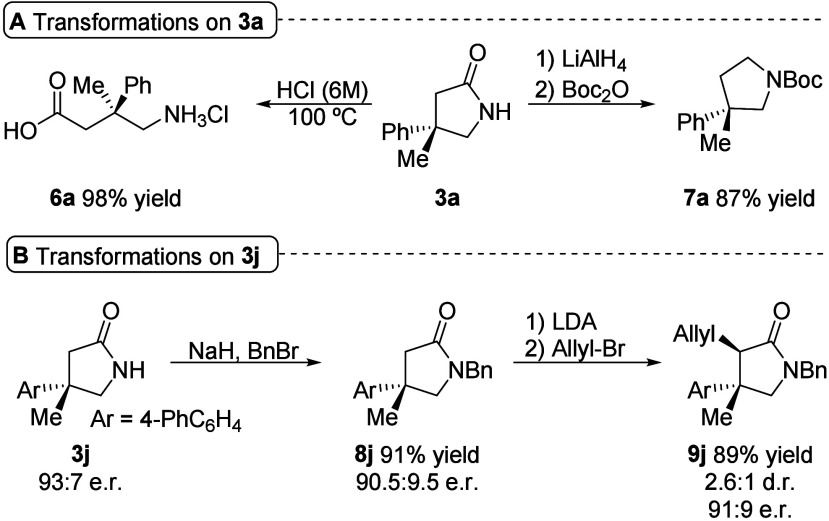
Transformations of Beckmann Rearrangement Products

As indicated previously, the stereocontrol of the final outcome of the reaction would rely on the optimal enantiocontrol of the axially chiral oxime sulfonate intermediate, and this enantioenriched intermediate would evolve in a subsequent step to the final γ-lactam product through axial-to-central chirality transfer. As the chiral oxime sulfonate intermediate presented limited stability that complicated its handling,^15^ some control experiments were carried out, beginning with the evaluation of the influence of water and an achiral Brønsted acid in the second stage of the reaction that involved the rearrangement. It was observed that after the formation of enantioenriched oxime ether **4d** under the optimal conditions, the addition of 1 equiv of *p*-TSA·H_2_O and heating rendered product **3a** in 70% yield together with appreciable amounts of starting ketone **1a**, while the er was 52:48 (Scheme 4A), which is significantly lower than that obtained under the optimized conditions (see Table 2, **3a**). These results indicate that oxime ether formation is reversible when water is present, as starting ketone **1a** is recovered. Furthermore, the fact that the enantioenriched oxime sulfonate intermediate loses enantiopurity is explained in terms of the formation of an achiral product arising from the addition of water to the C=N bond, which in the presence of an achiral catalyst evolves through a nonstereocontrolled fashion to afford product **3a**. In a different experiment, and in order to examine whether the chiral Brønsted acid **5b** could induce enantioselection during the rearrangement step, racemic oxime sulfonate *rac* **-4d** was treated under the optimal conditions, delivering the γ-lactam product in 63% yield and 53:47 er (Scheme 4B). This result indicates that chiral catalyst **5b** has a negligible effect on the enantioselection once axially chiral oxime sulfonate intermediate **4a** is formed.^16^

**Scheme 4:**
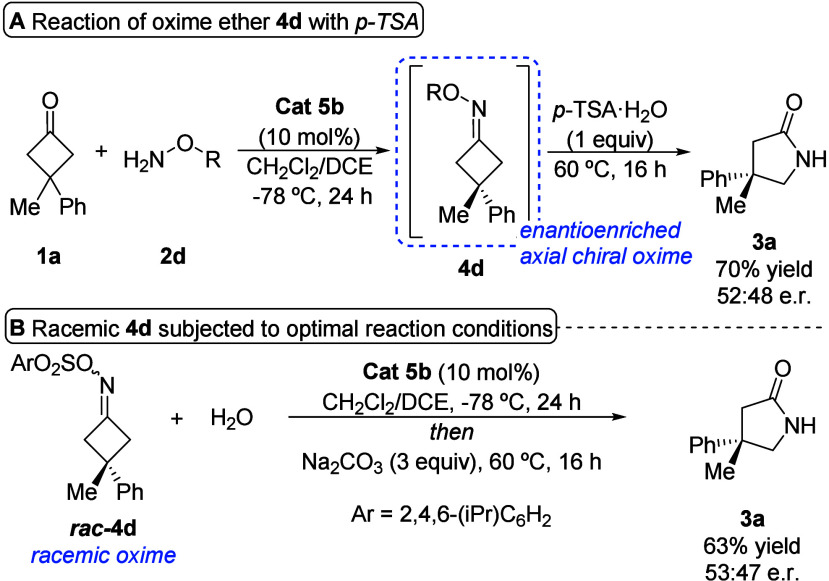
Evaluation of the Evolution of the Oxime Ether Intermediate under Different Conditions

In conclusion, an enantioselective version of the Beckmann rearrangement is presented through the desymmetrization of cyclobutanones *via* axial-to-point chirality transfer. The transformation entails two steps. The chiral BINOL-derived *N*-triflyl phosphoramide catalyst promotes the formation of an enantioenriched axially chiral oxime ether intermediate, which evolves through Beckmann rearrangement to the final central chiral γ-lactam product featuring a quaternary stereocenter. The transformation is efficient for accessing products presenting quaternary stereocenters with stereoselectivities that range from moderate to excellent. The scope of the method is broad, regarding (hetero)­aromatic substituents of diverse stereoelectronic nature, although a strong influence on the enantioselectivity is observed for monosubstituted 3-substituted cyclobutanones. The products were subjected to various transformations, taking advantage of the polyfunctionality that the lactam presents, outlining potential uses of this methodology as an enantioselective procedure to access GABA analogues with a quaternary stereocenter. In summary, this reaction platform enables the skeletal editing of cyclic ketones by enantioselectively inserting a nitrogen atom into a ring-expanding transformation, rendering optically active γ-lactams from achiral and easily accessible starting materials.

## Supplementary Material



## Data Availability

The data underlying this study are available in the published article and its Supporting Information.

## Notes

Experimental procedures, characterization of all new compounds, copies of ^1^H and ^13^C NMR spectra, and HPLC traces of all adducts prepared (PDF)
