# Supplementary material for: Enantioselective Beckmann Rearrangement through Organocatalytic Desymmetrization of Cyclobutanones for the Synthesis of 4,4-Disubstituted γ‑Lactams
Source: Org Lett. 2025 Dec 23;28(1):338–42. doi: 10.1021/acs.orglett.5c04735 (PMC12797323; doi:10.1021/acs.orglett.5c04735)
Supplement: Supplementary file 1 [file ol5c04735_si_001.pdf]

Supplementary Information for:

Enantioselective Beckmann Rearrangement through Organocatalytic Desymmetrization of Cyclobutanones for the Synthesis of 4,4-disubstituted γ-lactams

Antón Igartua, Liher Prieto,* Uxue Uria, Luisa Carrillo, Efraim Reyes and Jose L. Vicario*

Department of Organic and Inorganic Chemistry, University of the Basque Country (EHU), P.O. Box 644, 48080 Bilbao, (Spain)

Contents

1. General Information and Materials page ESI-2
2. Optimization of Reaction Conditions page ESI-3
3. Experimental Procedures and Characterizations page ESI-4
4. X-Ray Analysis of Compound **3e** page ESI-20
5. NMR Spectra page ESI-21
6. HPLC Traces page ESI-59

1. General information and materials.[1]

Analytical grade solvents and commercially available reagents were used without further purification. Anhydrous solvents were purified and dried over activated molecular sieves prior to use. For reactions under inert conditions, argon was used, which was previously dried by passing through a column of KOH, $CaCl_2$ and Silica Gel 3-5 mm with indicator (Cobalt Chloride). The glassware was dried in an oven at 140 ºC for 12 hours, and allowed to cool under a dehumidified atmosphere.[2] Reactions were monitored using analytical thin layer chromatography (TLC), in pre-coated silica-backed plates (Merck Kieselgel 60 F254). These were visualized by ultraviolet irradiation (254 and 366 nm), potassium permanganate or *p*-anisaldehyde dips.[3] For flash chromatography silica gel (Siliaflash 40-63, 230-400 mesh) was used.[4] For the removal of solvents under reduced pressure Büchi R-210 rotary evaporators were used. For precision weighting Sartorius Analytical Balance was used (±0.1 mg). For reaction heating Stuart™ Hotplate block insert was used.

NMR: Monodimensional nuclear magnetic resonance proton and carbon spectra (^{1}H NMR,^{13}C NMR and ^{19}F NMR) were acquired at 25ºC on a Bruker AC-300 spectrometer (300 MHz for ^{1}H, 75.5 MHz for ^{13}C and 282 MHz for ^{19}F) and a Bruker AC-500 spectrometer (500 MHz for ^{1}H and and 125.7 MHz ^{13}C). Chemical shifts (δ) are reported in ppm relative to residual solvent signals,[5] ($CDCl_3$, 7.26 ppm for ^{1}H NMR, $CDCl_3$, 77.0 ppm for ^{13}C NMR); and coupling constants (*J*) in hertz (Hz). The following abbreviations are used to indicate the multiplicity in ^{1}H NMR spectra: s, singlet; d, doublet; t, triplet; q, quartet; m, multiplet; bs, broad signal. ^{13}C NMR spectra were acquired on a broad band decoupled mode using DEPT experiments (Distortionless Enhancement by Polarization Transfer) for nucleus assignment.

IR: Infrared spectra (IR) were measured in a Jasco FT/IR 4100, in the interval between 4000 and 650 cm^{-1} with a 4 cm^{-1} resolution. Only characteristic bands are given in each case.

MS: Mass spectra (MS) were recorded on an Agilent 7890A gas chromatograph coupled to an Agilent 5975 mass spectrometer under electronic impact (EI) conditions at 70 eV. The obtained data is presented in mass units (m/z) and the values found in brackets belong to the relative intensities comparing to the base peak (100%).

HRMS: High-resolution mass spectra (HRMS) were acquired on an Acquity UPLC coupled to a QTOF mass spectrometer (SYNAPT G2 HDMS) using electrospray ionization (ESI^+ or ESI^-) or LC/Q-TOF with ESI Agilent Jet Stream source.

MP: Melting points were measured in a Büchi B-540 apparatus in open capillary tubes and are uncorrected.

HPLC: High performance liquid chromatography on a chiral stationary phase was performed in a Waters 2695 chromatograph coupled to a Waters 2998 photodiode array detector. Daicel Chiralpak *ASH, IA, ID-3, IF-3, OD-3, OZ-3* columns (0.46 cm x 25 cm) were used; specific conditions are indicated for each case.

X-ray data collections were performed in an Agilent Supernova diffractometer equipped with an Atlas CCD area detector, and a CuKα micro-focus source with multilayer optics (λ = 1.54184Å, 250μm FWHM beam size). The sample was kept at 120 K with a Oxford Cryosystems Cryostream 700 cooler. The quality of the crystals was checked under a polarizing miscroscope, and a suitable crystal or fragment was mounted on a Mitegen Micromount™ using Paratone N inert oil and transferred to the diffractometer.

Specific rotations $[\alpha]_D^{20}$: were measured at 20 ºC on a Jasco P-2000 polarimeter with sodium lamp at 589 nm and a path of length of 1 dm. Solvent and concentration are specified in each case.

---

[1] The authors thank for technical and human support provided by SGIker of UPV/EHU (NMR, LC/MS and X-ray analysis), MEC, GV/EJ and European Social Fund is also gratefully acknowledged.

[2] Kramer, G. W.; Levy, A. B.; Midland, M. M. *Organic Synthesis via Boranes*; John Wiley & Sons: New York, 1975.

[3] Stahl, E. *Thin Layer Chromatography*; Springer-Verlag: Berlin, 1969.

[4] Still, W. C.; Kahn, H.; Mitra, A. J. Rapid Chromatographic Technique. *J. Org. Chem.* **1978**, *43*, 2923–2925.

[5] Gottlieb, H. E.; Kotlyar, V.; Nudelman, A. NMR Chemical Shifts of Common Laboratory Solvents as Trace Impurities. *J. Org. Chem.* **1997**, *62*, 7512–7515.

2. Optimization of Reaction Conditions

Table SI-1. Optimization of Reaction Conditions Enantioselective Synthesis of 4,4-disubstituted γ-lactams through Beckmann Rearrangement.[a]

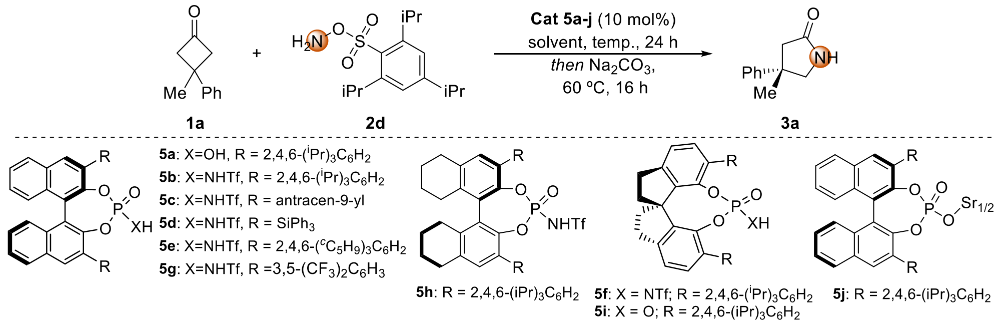

Entry	Catalyst	Solvent	Temp. (°C)	Yield (%)[b]	e.r.[c]
1	**5a**	CH_2Cl_2	23	55	50:50
2	**5a**	CH_2Cl_2	-78	35	67:34
3	**5b**	CH_2Cl_2	23	75	50:50
4	**5b**	CH_2Cl_2	-78	71	86.5:13.5
5	**5c**	CH_2Cl_2	-78	70	68:32
6	**5d**	CH_2Cl_2	-78	20	54.5:45.5
7	**5e**	CH_2Cl_2	-78	51	84:16
8	**5g**	CH_2Cl_2	-78	9	59:41
9	**5h**	CH_2Cl_2	-78	37	55:45
10	**5b**	$CHCl_3$	-78	62	83:17
11	**5b**	DCE	-35	56	86:14
12	**5b**	Toluene	-78	53	76:24
13	**5b**	EtOAc	-78	68	55:45
14	**5b**	THF	-78	59	57.5:42.5
15[d]	**5b**	CH_2Cl_2/DCE	-78	86	91.5:8.5
16[d]	**5f**	CH_2Cl_2/DCE	-78	83	80:20
17[d]	**5i**	CH_2Cl_2/DCE	-78	84	61:39
18[d]	**5j**	CH_2Cl_2/DCE	-78	37	53:47
18[d]	**5j**	CH_2Cl_2/DCE	-78	37	52.5:47.5

[a] Reaction conditions: **1a** (0.1 mmol), **2e** (0.12 mmol), **5a-g** (0.01 mmol), in 0.5 mL of solvent, at the mentioned temperature for 24 h, followed by addition of Na_2CO_3 (0.3 mmol) and heating to 60 °C for 16 h in sealed tube. DCE = 1,2-dichloroethane [b] Determined after chromatographic purification. [c] Determined by chiral HPLC analysis (see SI for details). [d] A 1:1 mixture of CH_2Cl_2/DCE was used as solvent.

3. Experimental Procedures and Characterizations.

3.1. Synthesis of 3-substituted cyclobutanone derivatives 1a-r.

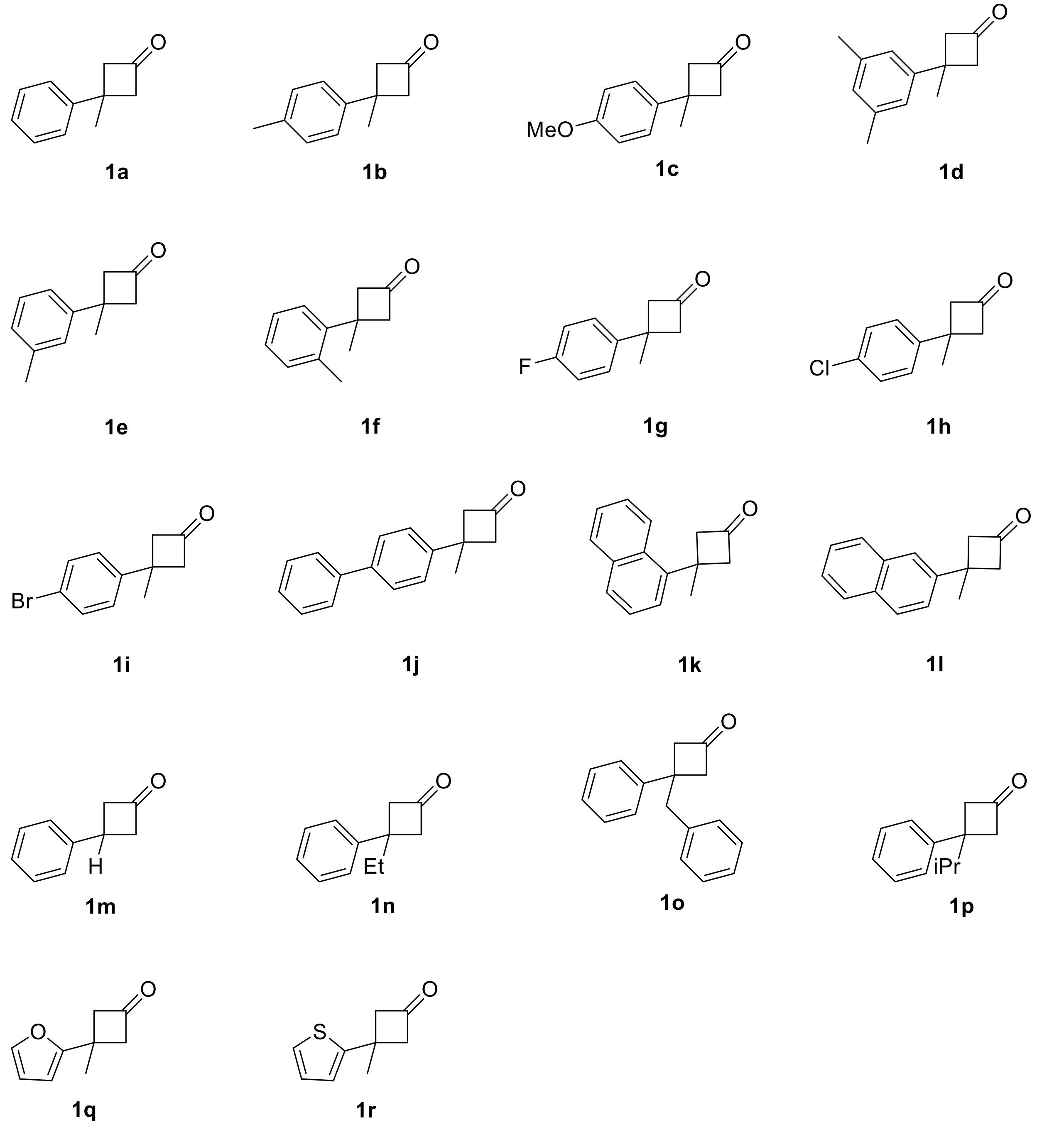

Figure SI-1. 3-substituted cyclobutanone derivatives **1a-r** used in this work.

3-substituted cyclobutanone derivatives were prepared starting from commercially available styrenes following a modified literature procedure.[6]

[6] Le Vaillant, F.; Garreau, M.; Nicolai, S.; Gryn'ova, G.; Corminboeuf, C.; Waser, J. Fine-Tuned Organic Photoredox Catalysts for Fragmentation-Alkynylation Cascades of Cyclic Oxime Ethers. *Chem. Sci.* **2018**, *9*, 5883–5889.

General Procedure A

Zinc powder (4.0 equiv) was suspended in diethyl ether (0.18M in respect to the styrene) under inert atmosphere. After the addition of the styrene (1.0 equiv), a solution of trichloroacetyl chloride (2.0 equiv) in diethyl ether (0.55M) was added dropwise over a period of 40-45 minutes. During this time, the mixture was irradiated with ultrasound, while being maintained at a temperature below 25 °C by using a thermostated water bath. Once the addition was completed, the mixture was kept under sonication for 1-4 h until full conversion confirmed by TLC (elution with hexane, $KMnO_4$ stain), accompanied by a change in color to a dark orange or brown. The reaction was diluted with diethyl ether. The solids were filtered through a plug of celite and washed with diethyl ether. The filtrate was then washed twice with water, four times with sat. aq. $NaHCO_3$, and brine; it was then dried over anhydrous $NaSO_4$, filtered and concentrated under vacuum. Column chromatography (Hexanes:EtOAc) furnished the corresponding 2,2-dichlorocyclobutanone intermediates.

To a suspension of zinc (5 equiv.) and NH_4Cl (2.5 equiv.) in methanol (1.76 M for NH_4Cl) was added dropwise a mixture of the corresponding 2,2-dichlorocyclobutanone (1 equiv.) in methanol (0.7 M) keeping the temperature at 0 ºC. The solution was then heated to 70 ºC for 3 hours using a Stuart™ Hotplate block insert. The mixture was cooled down to room temperature and filtrated with celite, and the solvent was evaporated. The obtained solid was diluted with HCl (1M), and the aqueous layer was extracted with diethyl ether three times and the organic layer was washed with brine aqueous solution. The organic extracts were dried over anhydrous Na_2SO_4, filtered and the solvent was evaporated. The crude compound was purified by flash column chromatography in silica gel and the solvent was evaporated to obtain the corresponding cyclobutanone.

Ketones **1a**,[6] **1b**,[7] **1c**,[10] **1d**,[8] **1e**,[9] **1f**,[10] **1g**,[10] **1h**,[11] **1j**,[10]**¡Error! Marcador no definido.** **1k**,[7] **1l**,[11] **1m**,[12] **1n**[7] and **1o**[8] were synthetized according to literature procedures and their spectroscopic data was in agreement with that reported in the literature.

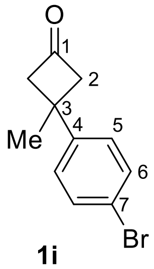

1i

3-methyl-3-phenylcyclobutan-1-one (1i)

Following the *general procedure A*, using 1-bromo-4-(prop-1-en-2-yl)benzene (1.61 g, 8.15 mmol), **1i** (716 mg, 2.99 mmol) was isolated by flash chromatography (petroleum ether/EtOAc gradient 50:1 to 95:5) as a white solid. Yield: 37%. Rf (petroleum ether/EtOAc 50:1): 0.33. **^{1}H NMR** (300 MHz, $CDCl_3$) δ 7.52 – 7.45 (m, 2H), 7.22 – 7.15 (m, 2H), 3.50 – 3.35 (m, 2H), 3.19 – 3.04 (m, 2H), 1.59 (s, 3H). **^{13}C NMR** (75 MHz, CDCl3) δ 205.7, 147.3, 131.7, 127.6, 120.2, 59.2, 33.8, 30.9). **IR** (ATR, cm^{-1}): 2959, 2923, 2866, 1782. **HRMS** (ESI) m/z: $[M + H]^+$ Calcd for $C_{11}H_{12}BrO$ 239.0066; Found 239.0065. **MP**: 35 – 37 ºC.

[7] Capel, E.; Rodríguez-Rodríguez, M.; Uria, U.; Pedrón, M.; Tejero, T.; Vicario, J. L.; Merino, P. Catalyzed Desymmetrizative Ring Expansion of 1-Vinylcyclobutanols: Absence of Intermediates and Mechanistic Insights. *J. Org. Chem.* **2022**, *87*, 693–707.

[8] Ano, Y.; Takahashi, D.; Yamada, Y.; Chatani, N. Palladium-Catalyzed Skeletal Rearrangement of Cyclobutanones via C–H and C–C Bond Cleavage. *ACS Catalysis*, **2023**, *13*(4), 2234. Ano, Y.; Takahashi, D.; Yamada, Y.; Chatani, N. *ACS Catal.* **2023**, *13* (4), 2234–2239.

[9] Matsuda, T.; Yuihara, I. Rhodium(I)-Catalysed Formal Intramolecular C−C/C−H Bond Metathesis. *Chemical Communications*, **2015**, *51*, 7393–7396.

[10] Ma, Y.; Ai, Y.; Yu, S. Chemoselective Strain Release of Bicyclo[1.1.1]pentanyl Alcohols. *Synlett* **2023**, *34*, 359–363.

[11] Seiser, T.; Cramer, N. Enantioselective C-C bond activation of allenyl cyclobutanes: access to cyclohexenones with quaternary stereogenic centers. *Angew. Chem. Int. Ed.* **2008**, *47*, 9294–9297.

[12] Featherston, A. L.; Shugrue, C. R.; Mercado, B. Q.; Miller, S. J. Phosphothreonine (pThr)-Based Multifunctional Peptide Catalysis for Asymmetric Baeyer-Villiger Oxidations of Cyclobutanones. *ACS Catal.* **2019**, *9*, 242–252.

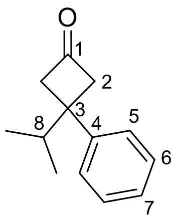

1p

3-isopropyl-3-phenylcyclobutan-1-one (1p)

Following *general procedure A*, using (3-methylbut-1-en-2-yl)benzene (9.15 g, 63.0 mmol) as starting material, **1p** (2.34g, 18.0 mmol) was isolated by flash chromatography (petroleum ether/EtOAc 50:1) as a yellowish oil. Yield: 29%. Rf (petroleum ether/EtOAc 92:08): 0.48. ^{1}**H NMR** (300 MHz, $CDCl_3$) δ 7.29 – 7.08 (m, 5H), 3.38 – 3.30 (m, 2H), 3.27 – 3.19 (m, 2H), 1.90 (hept, *J* = 6.8 Hz, 1H), 0.75 (d, *J* = 6.7 Hz, 6H). ^{13}**C-NMR** (75 MHz, $CDCl_3$) δ 207.2, 143.7, 128.4, 127.8, 126.3, 57.0, 42.2, 37.5, 18.0. **IR** (ATR) (cm^{-1}): 3026, 2966, 1778, 1494, 1379, 1106, 948. **HRMS** (ESI) m/z: $[M + H]^+$ Calcd for $C_{13}H_{17}O$ 189.1274; Found 189.1277.

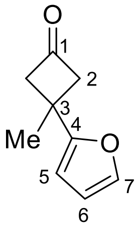

1q

3-methyl-3-(furan-2-yl)cyclobutan-1-one (1q)

Following the *general procedure A*, using 2-(prop-1-en-2-yl)furane (292 mg, 2.7 mmol) as starting material, **1q** (154 mg, 1.0 mmol) was isolated by flash chromatography (petroleum ether/EtOAc 50:1 to 95:5) as a colourless oil. Yield: 37%. Rf (PE/EA 95:5): 0.38. ^{1}**H NMR** (300 MHz, $CDCl_3$) δ 7.36 (dd, *J* = 1.9, 0.8 Hz, 1H), 6.32 (dd, *J* = 3.2, 1.9 Hz, 1H), 6.13 (dd, *J* = 3.2, 0.9 Hz, 1H), 3.58 – 3.43 (m, 2H), 3.08 – 2.93 (m, 2H), 1.68 (s, 3H). ^{13}**C NMR** (75 MHz, $CDCl_3$) δ 206.3, 159.5, 141.7, 110.2, 104.2, 59.0, 28.9, 25.5. **IR** (ATR) (cm^{-1}): 2966, 2930, 2874, 2852, 1783, 1380, 716. **HRMS** (ESI) m/z: $[M + H]^+$ Calcd for $C_9H_{11}O_2$ 151.0754; Found 151.0752.

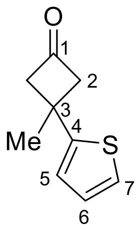

1r

3-methyl-3-(thiophen-2-yl)cyclobutan-1-one (1r)

Following the *general procedure A*, using 2-(prop-1-en-2-yl)thiophene (3.11 g, 25.0 mmol) as starting material, **1r** (1.88 g, 11.4 mmol) was isolated by flash chromatography (petroleum ether/EtOAc 95:5) as a yellowish oil. Yield: 46%. Rf (PE/EA 95:5): 0.52. ^{1}**H NMR** (300 MHz, $CDCl_3$) δ 7.20 (dd, *J* = 5.0, 1.3 Hz, 1H), 6.96 (dd, *J* = 5.0, 3.5 Hz, 1H), 6.92 (dd, *J* = 3.5, 1.3 Hz, 1H), 3.56 – 3.41 (m, 2H), 3.25 – 3.10 (m, 2H), 1.77 (s, 3H). ^{13}**C NMR** (75 MHz, $CDCl_3$) δ 206.1, 153.0, 127.1, 123.9, 123.1, 61.9, 31.4, 30.1. **IR** (ATR) (cm^{-1}): 3063, 2962, 1778, 1443, 1379, 1131, 693. **HRMS** (ESI) m/z: $[M – H_2O + H]^+$ Calcd for C_9H_9S 149.0419; Found 149.0396.

3.2. Synthesis of *O*-arylsulfonylhydroxylamine derivatives 2a-b.

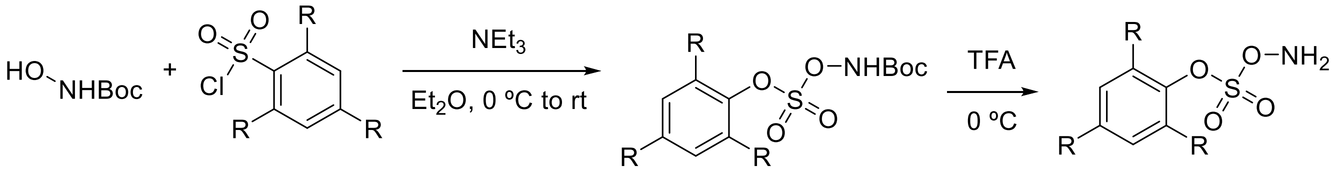

SI-1a R: -Me
SI-1b R: -*i*Pr

2c R: -Me
2d R: -*i*Pr

Figure SI-2. *O*-arylsulfonylhydroxylamine derivatives **2c-d** used in this work.

General Procedure B

tert-Butyl *N*-arylsulfonyloxycarbamate derivatives **SI-1a-b** were prepared following a modified literature procedure.[13] To a solution of sulfonyl chloride (1.0 equiv) and *tert*-butyl-*N*-hydroxycarbamate (1.0 equiv) in dichloromethane (0.43 M) at 0 ºC, triethylamine (1.0 equiv) was dropwise added and stirred for 2 h. The reaction was quenched with NH_4Cl, extracted with dichloromethane (3 x 15 mL) and dried over anhydrous Na_2SO_4. The obtained solid was purified through column chromatography (PE/EtOAc 95:5 to 8:2).

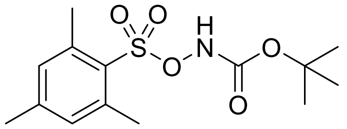

SI-1a

***tert*-Butyl *N*-(2,4,6-trimethylphenyl)sulfonyloxycarbamate (SI-1a)** Following *general procedure B* using mesitylsulfonyl chloride (555 mg, 2.53 mmol), **SI-1a** (588 mg, 1.87 mmol) was isolated as a white solid. Yield: 74%. Rf (PE/EtOAc 9:1): 0.47. **^{1}H NMR** (300 MHz, CDCl3) δ 7.51 (s, 1H), 6.99 (s, 2H), 2.68 (s, 6H), 2.32 (s, 3H), 1.32 (s, 9H). **^{13}C NMR** (75 MHz, $CDCl_3$) δ 154.5, 144.4, 141.9, 131.6, 128.5, 83.7, 27.7, 23.1, 21.1. **IR** (ATR, cm^{-1}): 3289, 2981, 2937, 1768, 1732, 1703, 1456, 1367, 1246, 1193, 1178, 1156, 747, 656. **HRMS** (ESI) m/z: $[M + Na]^+$ Calcd for $C_{14}H_{21}NO_5SNa$ 338.1033; Found 338.1041. **MP**: 104 – 106 ºC.

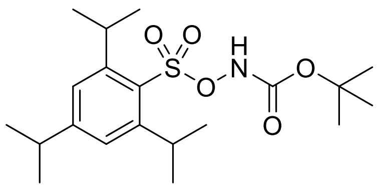

SI-1b

***tert*-Butyl *N*-(2,4,6-triisopropylphenyl)sulfonyloxycarbamate (SI-1b)** Following *general procedure B* using 2,4,6-triisopropylbenzenesulfonyl chloride (8.37 g, 26.6 mmol) **SI-1b** (9.6 g, 24.0 mmol) was isolated as a white solid. Yield: 90%. Rf (PE/EtOAc 9:1): 0.72. **^{1}H NMR** (300 MHz, CDCl3) δ 7.47 (s, 1H), 7.20 (s, 2H), 4.12 (hept, *J* = 6.7 Hz, 2H), 2.92 (hept, *J* = 6.9 Hz, 1H), 1.40 (s, 9H), 1.27 (t, *J* = 6.9 Hz, 18H). **^{13}C NMR** (75 MHz, $CDCl_3$) δ 154.7, 154.2, 152.4, 127.7, 123.9, 83.7, 34.3, 30.1, 27.9, 24.8, 23.5. **IR** (ATR, cm^{-1}): 3297, 2966, 2930, 2873, 1767, 1702, 1382, 1369, 1353, 1185, 1155, 738, 659. **HRMS** (ESI) m/z: $[M + Na]^+$ Calcd for $C_{20}H_{33}NO_5SNa$ 422.1972; Found 422.1982. **MP**: 126 – 128 ºC.

General Procedure C

The corresponding *N*-Boc-*O*-sulfonylhydroxylamine **SI-1a-b** (1.0 mmol, 1.0 equiv.) was added as a solid portion wise to TFA (3.0 mL, 0.33 M) at 0ºC while stirring. After 1h the mixture was poured onto ice-water (30 mL) for 15 min. The precipitate is vacuum filtered and dried overnight under vacuum to obtain the corresponding *O*-arylsulfonylhydroxylamine **1c-d** as a white solid. These were used without further purification.

[13] Mendiola, J.; Rincón, J. A.; Mateos, C.; Soriano, J. F.; de Frutos, Ó.; Niemeier, J. K.; Davis, E. M. Preparation, Use, and Safety of *O*-Mesitylenesulfonylhydroxylamine. *Org. Process Res. Dev.* **2009**, *13* (2), 263–267.

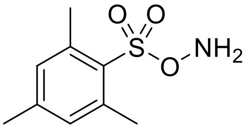

2c

***O*-((2,4,6-trimethylphenyl)sulfonyl)hydroxylamine (2c)**

Following *general procedure C* using *tert*-Butyl N-(2,4,6-triisopropylphenyl)sulfonyloxycarbamate **SI-1a** (200.0 mg, 0.63 mmol), **2b** (83.0 mg, 0.39 mmol) was isolated as a white solid. Yield: 61%. Rf (PE/EtOAc 9:1): 0.20. **^{1}H NMR** (300 MHz, CDCl3) δ 6.99 (s, 2H), 5.73 (s, 2H), 2.63 (s, 6H), 2.32 (s, 3H). **^{13}C NMR** (75 MHz, $CDCl_3$) δ 143.8, 141.0, 131.7, 129.1, 22.7, 21.1. **IR** (ATR, cm^{-1}): 3337, 3270, 2991, 1598, 1454, 1346, 1212, 1187, 1173, 854, 776, 654. **HRMS** (ESI) m/z: $[M + Na]^+$ Calcd for $C_9H_{13}NO_3SNa$ 238.0508; Found 238.0514. **MP:** Decomp. exploded at 60 ºC.

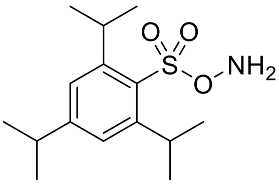

2d

***O*-((2,4,6-triisopropylphenyl)sulfonyl)hydroxylamine (2d)**

Following *general procedure C* using *tert*-butyl *N*-(2,4,6-triisopropylphenyl)sulfonyloxycarbamate **SI-1b** (400.0 mg, 1.0 mmol), **2b** (234.0 mg, 0.78 mmol) was isolated as a white solid. Yield: 79%. Rf (PE/EtOAc 9:1): 0.55. **^{1}H NMR** (300 MHz, CDCl3) δ 7.21 (s, 2H), 5.76 (s, 2H), 4.12 (hept, J = 6.8 Hz, 2H), 2.92 (hept, J = 7.0 Hz, 1H), 1.27 (dd, J = 6.8, 1.6 Hz, 18H). **^{13}C NMR** (75 MHz, $CDCl_3$) δ 154.2, 151.8, 127.7, 123.9, 77.4, 77.0, 76.6, 34.3, 29.8, 24.8, 23.5. **IR** (ATR, cm^{-1}): 3331, 3264, 2956, 2930, 2869, 1374, 1343, 1330, 1224, 1192, 1180, 1010, 861, 784, 757, 682, 660. **HRMS** (ESI) m/z: $[M + Na]^+$ Calcd for $C_{15}H_{25}NO_3SNa$ 322.1447; Found 322.1451. **MP:** Decomp. exploded at 90 ºC.

3.3. Synthesis of Brønsted acid catalysts 5a-j.

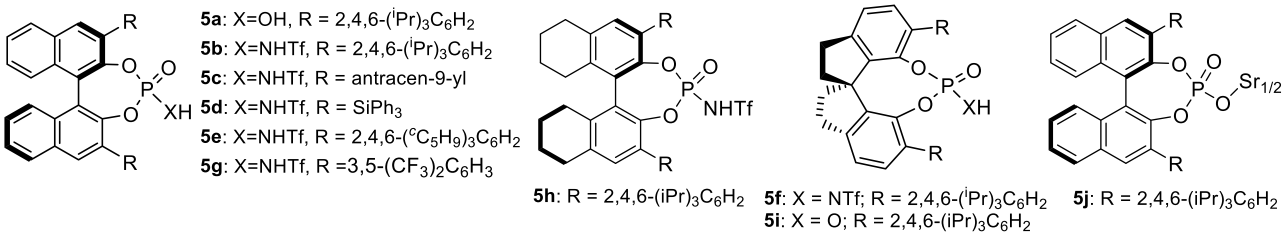

Figure SI-3. Brønsted acid catalysts **5a-j** used in this work.

The catalysts **5a**,[14] **5c**,[15] **5d**,[16] **5f**,[17] **5g**,[15] **5h**[18] and **5i**[19] were commercially available, **5a**, **5c**, **5g** and **5h** were purchased from Sigma-Aldrich, **5d**, **5f** and **5i** were purchased from BLDpharm and used as received without further purification.

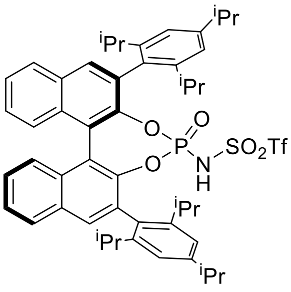

(*R*)-5b

Triflurormethylsulfonyl phosphoramide **5b** was prepared following a reported literature procedure.[20] The spectroscopic data was in agreement with those reported in the literature. **^{1}H NMR** (300 MHz, CDCl$_3$) δ 7.97 (d, *J* = 9.7 Hz, 4H), 7.56 (ddd, *J* = 8.0, 6.4, 1.3 Hz, 2H), 7.39 – 7.26 (m, 4H), 7.15 (dd, *J* = 9.9, 1.7 Hz, 3H), 7.06 (d, *J* = 1.7 Hz, 1H), 2.94 (ddq, *J* = 10.4, 6.9, 3.5 Hz, 2H), 2.85 – 2.48 (m, 4H), 1.29 (dd, *J* = 6.8, 4.7 Hz, 18H), 1.21 (dd, *J* = 6.8, 1.6 Hz, 6H), 1.14 (dd, *J* = 15.5, 7.0 Hz, 6H), 0.98 (dd, *J* = 13.4, 6.8 Hz, 6H).**^{19}F NMR** (282 MHz, CDCl$_3$) δ -77.3. **^{31}P NMR** (122 MHz, CDCl$_3$) δ -3.58.
δ

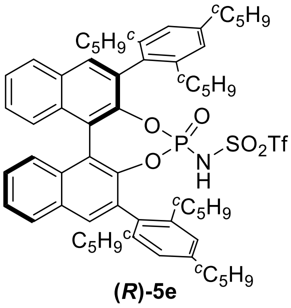

(*R*)-5e

Triflurormethylsulfonyl phosphoramide **5e** was prepared following a reported literature procedure.[21] **^{1}H NMR** (300 MHz, CDCl$_3$) δ 7.91 (d, *J* = 8.2 Hz, 2H), 7.80 (s, 2H), 7.48 (dq, *J* = 8.9, 5.0, 4.5 Hz, 2H), 7.31 (d, *J* = 3.9 Hz, 4H), 7.02 (d, *J* = 11.1 Hz, 4H), 6.21 (s, 1H), 3.09 – 2.89 (m, 2H), 2.82 – 2.55 (m, 4H), 2.19 – 1.88 (m, 8H), 1.80 – 1.55 (m, 8H), 1.87 – 1.40 (m, 32H), 1.39 – 1.14 (m, 8H). **^{19}F NMR** (282 MHz, CDCl$_3$) δ -77.2. **^{31}P NMR** (122 MHz, CDCl$_3$) δ 5.31.

---

[14] Seayad, J.; Seayad, A. M.; List, B. Catalytic Asymmetric Pictet–Spengler Reaction. *J. Am. Chem. Soc.* **2006**, *128*, 1086–1087.

[15] Rueping, M.; Lin, M.-Y. Catalytic Asymmetric Mannich-Ketalization Reaction: Highly Enantioselective Synthesis of Tetrahydroisoquinolines. *Chem. Eur. J.* **2010**, *16*, 4169–4172.

[16] Villar, L.; Uria, U.; Martínez, J. I.; Prieto, L.; Reyes, E.; Carrillo, L.; Vicario, J. L. Enantioselective Oxidative (4+3) Cycloadditions between Allenamides and Furans through Bifunctional Hydrogen-Bonding/Ion-Pairing Interactions *Angew. Chem. Int. Ed.* **2017**, *56*, 10535–10538.

[17] Hong, X.; Küçük, H. B.; Maji, M. S.; Yang, Y. F.; Rueping, M.; Houk, K. N. Mechanism and Selectivity of N-Triflylphosphoramide Catalyzed (3+ + 2) Cycloaddition between Hydrazones and Alkenes. *J. Am. Chem. Soc.* **2014**, *136*, 13769–13780.

[18] Gicquiaud, J.; Abadie, B.; Dhara, K.; Berlande, M.; Hermange, P.; Sotiropoulos, J. M.; Toullec, P. Y. Brønsted Acid-Catalyzed Enantioselective Cycloisomerization of Arylalkynes. *Chem. Eur. J.* **2020**, *26*, 16266–16271.

[19] Romanov-Michailidis, F.; Romanova-Michaelides, M.; Pupier, M.; Alexakis, A. Enantioselective Halogenative Semi-Pinacol Rearrangement: Extension of Substrate Scope and Mechanistic Investigations. *Chem. Eur. J.* **2015**, *21*, 5561–5583.

[20] Nakashima, D.; Yamamoto, H. Design of Chiral N-Triflyl Phosphoramide Catalysts for Enantioselective Reactions. *J. Am. Chem. Soc.* **2006**, *128*, 9626–9627.

[21] Jolit, A.; Dickinson, C. F.; Kitamura, K.; Walleser, P. M.; Yap, G. P. A.; Tius, M. A. Catalytic Enantioselective Nazarov Cyclization. *Eur. J. Org. Chem.* **2017**, 6067–6076.

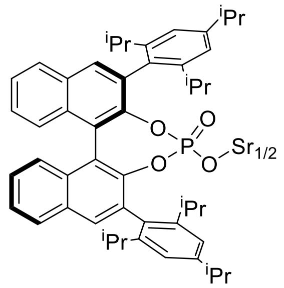

(*R*)-5j

Trifluroromethylsulfonyl phosphoramide **5j** was prepared following a reported literature procedure.[22] **^{1}H NMR** (300 MHz, DMSO-d6) δ 7.97 (d, *J* = 8.1 Hz, 2H), 7.77 (s, 2H), 7.39 (t, *J* = 7.5 Hz, 2H), 7.33 – 7.21 (m, 2H), 7.10 (d, *J* = 1.8 Hz, 2H), 7.07 – 6.97 (m, 4H), 2.92 (dt, *J* = 10.2, 6.6 Hz, 4H), 2.58 (q, *J* = 6.7 Hz, 2H), 1.27 (d, *J* = 6.9 Hz, 12H), 1.15 (dd, *J* = 6.8, 2.1 Hz, 12H), 1.07 (d, *J* = 6.9 Hz, 6H), 0.86 (d, *J* = 6.7 Hz, 6H). **^{31}P NMR** (122 MHz, $CDCl_3$) δ 3.42.

[22] Nimmagadda, S. K.; Zhang, Z.; Antilla, J. C. Asymmetric One-Pot Synthesis of 1,3-Oxazolidines and 1,3-Oxazinanes via Hemiaminal Intermediates. *Org. Lett.* **2014**, *16*, 4098–4101.

3.4. Preparation of lactam adducts 3a-s

General Procedure D

Into an oven-dried, screw-capped vial equipped with a magnetic stir bar, the corresponding ketone **1a-s** (0.10 mmol, 1.0 equiv), followed by the catalyst **(*R*)-5b** (0.01 mmol, 0.1 equiv) were weighed. Then, with the vial under positive argon pressure, a 1:1 mixture of dichloromethane and dichloroethane was added (0.5 mL) and the mixture was stirred at -78 ºC for 15 minutes. Then, arylsulfonylhydroxylamine **2d** (35.9 mg, 0.12 mmol, 1.2 equiv) was added under positive argon pressure in one portion. After 24 hours at -78 ºC, anhydrous Na_2CO_3 (31.8 mg, 0.3 mmol, 3.0 equiv) and the reaction was directly heated at 60 ºC using a Stuart™ Hotplate block insert while stirring for 16h. The reaction was quenched with HCl 1M at 60 ºC for 1 hour and then $NaHCO_3$ at room temperature. The aqueous layer was extracted with AcOEt (3 x 15 mL), dried over anhydrous Na_2SO_4 and evaporated. The crude reaction was dry loaded onto silica gel and subjected to column chromatography affording the corresponding adducts **3a-s**. Racemic samples for HPLC traces analysis were obtained following the above protocol at room temperature in the absence of a catalyst.

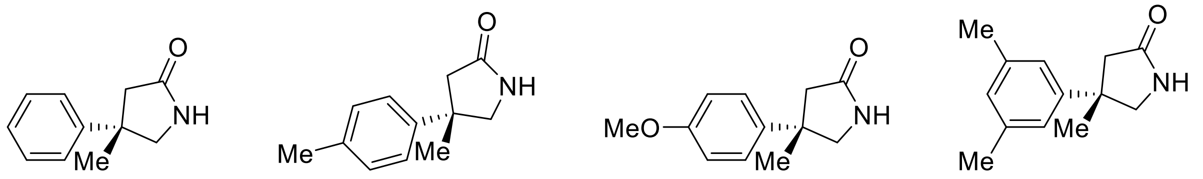

3a **3b** **3c** **3d**

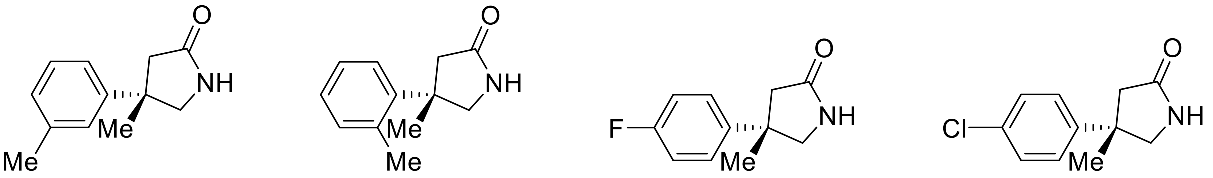

3e **3f** **3g** **3h**

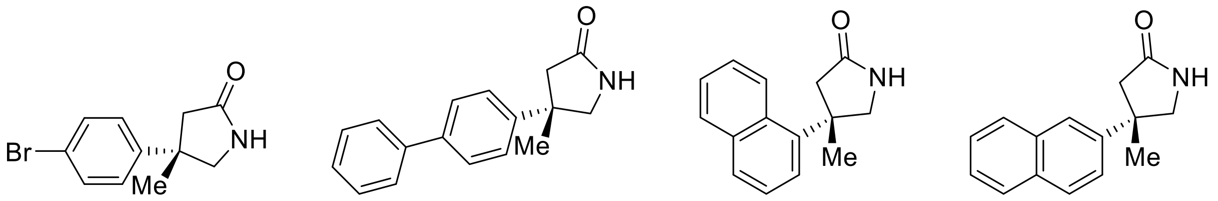

3i **3j** **3k** **3l**

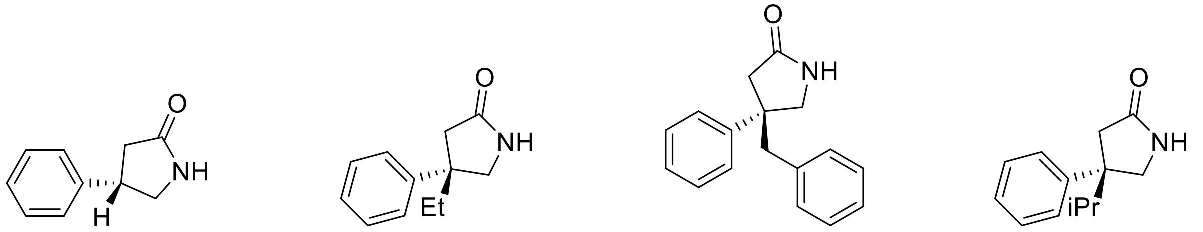

3m **3n** **3o** **3p**

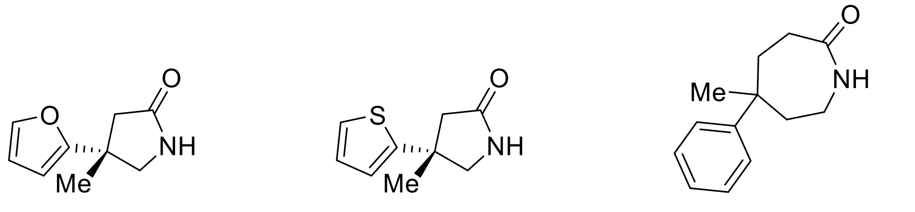

3q **3r** **3s**

Figure SI-4. Substituted lactam derivatives **3a-s** synthesized in this work.

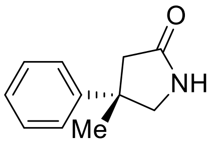

3a

(*S*)-4-methyl-4-phenylpyrrolidin-2-one (3a)

Following the *general procedure D* using 3-methyl-3-phenylcyclobutan-1-one **1a** (16.0 mg, 0.10 mmol), arylsulfonylhydroxylamine **2d** (35.9 mg, 0.12 mmol) and catalyst **(*R*)-5b** (8.8 mg, 0.01 mmol) in a 1:1 mixture of DCM/DCE (0.5 mL), **3a** (15.0 mg, 0.086 mmol) was isolated by flash chromatography (petroleum ether/EtOAc gradient 1:1 to 5% MeOH in EtOAc) as a white solid. Yield: 86%. e.r.: 91.5:8.5. For the 1 mmol scale reaction; using 3-methyl-3-phenylcyclobutan-1-one **1a** (160.0 mg, 1.0 mmol), arylsulfonylhydroxylamine **2d** (359.0 mg, 1.2 mmol) and catalyst **(*R*)-5b** (88.0 mg, 0.1 mmol) in a 1:1 mixture of DCM/DCE (5.0 mL), **3a** (114.1 mg, 0.651 mmol) was isolated by flash chromatography (petroleum ether/EtOAc gradient 1:1 to 5% MeOH in EtOAc) as a white solid. Yield: 65%. e.r.: 88:12. Rf (EtOAc): 0.56. **^{1}H NMR** (300 MHz, CDCl3) δ 7.40 – 7.32 (m, 2H), 7.29 – 7.19 (m, 3H), 5.99 (s, 1H), 3.68 (d, *J* = 9.3 Hz, 1H), 3.50 (dd, *J* = 9.3, 1.2 Hz, 1H), 2.79 (d, *J* = 16.3 Hz, 1H), 2.46 (d, *J* = 16.3 Hz, 1H), 1.51 (s, 3H). **^{13}C NMR** (75 MHz, $CDCl_3$) δ 177.1, 146.9, 128.8, 126.7, 125.3, 54.6, 44.2, 43.5, 29.8. **IR** (ATR, cm^{-1}): 3228, 3100, 3060, 3027, 2962, 2927, 2873, 1960, 764, 700. **HRMS** (ESI) m/z: $[M + H]^+$ Calcd for $C_{11}H_{14}NO$ 176.1070; Found 176.1073. **MP**: 102 – 104 °C. The e.r. was determined by HPLC (OD-3, *i*-PrOH/n-hexane = 10/90, flow rate = 1.0 mL/min, l = 254 nm) tR = 14.2 min (minor), 15.8 min (major). **$[\alpha]_D^{20}$** = +23.2 (*c* = 1.0, $CHCl_3$).

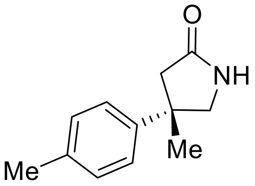

3b

(*S*)-4-methyl-4-(p-tolyl)pyrrolidin-2-one (3b)

Following the *general procedure D*, using 3-methyl-3-(p-tolyl)cyclobutan-1-one **1b** (17.4 mg, 0.10 mmol), arylsulfonylhydroxylamine **2d** (35.9 mg, 0.12 mmol) and catalyst **(*R*)-5b** (8.8 mg, 0.01 mmol) in a 1:1 mixture of DCM/DCE (0.5 mL), **3b** (10.2 mg, 0.054 mmol) was isolated by flash chromatography (petroleum ether/EtOAc gradient 1:1 to 5% MeOH in EtOAc) as a white solid. Yield: 54%. e.r.: 77.5:22.5. Rf (EtOAc): 0.54. **^{1}H NMR** (300 MHz, $CDCl_3$) δ 7.22 – 7.06 (m, 4H), 6.49 (s, 1H), 3.65 (d, *J* = 9.4 Hz, 1H), 3.47 (dd, *J* = 9.4, 1.1 Hz, 1H), 2.77 (d, *J* = 16.3 Hz, 1H), 2.43 (d, *J* = 16.3 Hz, 1H), 2.34 (s, 3H), 1.49 (s, 3H). **^{13}C NMR** (75 MHz, $CDCl_3$) δ 177.5, 144.0, 136.2, 129.4, 125.2, 54.9, 44.4, 43.1, 29.7, 20.9. **IR** (ATR, cm^{-1}): 3204, 3098, 3024, 2956, 2921, 2864, 1694, 815. **HRMS** (ESI) m/z: $[M + H]^+$ Calcd for $C_{12}H_{16}NO$ 190.1226; Found 190.1229. **MP**: 153 – 155 °C. The e.r. was determined by HPLC (OD-3, *i*-PrOH/n-hexane = 10/90, flow rate = 1.0 mL/min, l = 254 nm) tR = 11.2 min (minor), 13.5 min (major). **$[\alpha]_D^{20}$** = +14.7 (*c* = 1.0, $CHCl_3$).

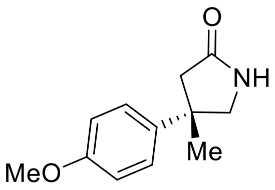

3c

(*S*)-4-(4-methoxyphenyl)-4-methylpyrrolidin-2-one (3c)

Following the *general procedure D*, using 3-(4-methoxyphenyl)-3-methylcyclobutan-1-one **1c** (19.0 mg, 0.10 mmol), arylsulfonylhydroxylamine **2d** (35.9 mg, 0.12 mmol) and catalyst **(*R*)-5b** (8.8 mg, 0.01 mmol) in a 1:1 mixture of DCM/DCE (0.5 mL), **3c** (16.4 mg, 0.08 mmol) was isolated by flash chromatography (petroleum ether/EtOAc gradient 1:1 to 5% MeOH in EtOAc) as a white solid. Yield: 80%. e.r.: 89:11. Rf (EtOAc): 0.39. **^{1}H NMR** (300 MHz, CDCl3) δ 7.19 – 7.08 (m, 2H), 6.93 – 6.82 (m, 2H), 6.57 (s, 1H), 3.80 (s, 3H), 3.63 (d, *J* = 9.4 Hz, 1H), 3.45 (dd, *J* = 9.4, 1.4 Hz, 1H), 2.75 (d, *J* = 16.3 Hz, 1H), 2.42 (d, *J* = 16.3 Hz, 1H), 1.48 (s, 3H). **^{13}C NMR** (75 MHz, $CDCl_3$) δ 177.6, 158.2, 139.0, 126.4, 114.0, 55.3, 55.0, 44.6, 42.8, 29.7. **IR** (ATR, cm^{-1}):3201, 3098, 3038, 2960, 2872, 2832, 1688, 1515, 1245, 1181, 1028, 827. **HRMS** (ESI) m/z: $[M + H]^+$ Calcd for $C_{12}H_{15}NO_2$ 206.1176; Found 206.1176. **MP**: 115 – 117 °C. The e.r. was determined by HPLC (ID-3, *i*-PrOH/n-hexane = 10/90, flow rate = 1.0 mL/min, l = 254 nm) tR = 21.6 min (major), 32.9 min (minor). **$[\alpha]_D^{20}$** = +27.0 (*c* = 1.0, $CHCl_3$).

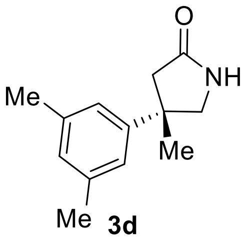

3d

(*S*)-4-(3,5-dimethylphenyl)-4-methylpyrrolidin-2-one (3d)

Following the *general procedure D*, using 3-(3,5-dimethyl)- 3-methylcyclobutan-1-one **1d** (18.8 mg, 0.10 mmol), arylsulfonylhydroxylamine **2d** (35.9 mg, 0.12 mmol) and catalyst **(*R*)-5b** (8.8 mg, 0.01 mmol) in a 1:1 mixture of DCM/DCE (0.5 mL), **3d** (10.5 mg, 0.052 mmol) was isolated by flash chromatography (petroleum ether/EtOAc gradient 1:1 to 5% MeOH in EtOAc) as a white solid. Yield: 52%. e.r.: 88.5:11.5. Rf (EtOAc): 0.57. **^{1}H NMR** (300 MHz, $CDCl_3$) δ 6.89 (s, 1H), 6.81 (s, 2H), 6.65 (s, 1H), 3.66 (d, *J* = 9.4 Hz, 1H), 3.46 (dd, *J* = 9.4, 1.4 Hz, 1H), 2.77 (d, *J* = 16.3 Hz, 1H), 2.42 (d, *J* = 16.3 Hz, 1H), 2.32 (s, 6H), 1.48 (s, 3H). **^{13}C NMR** (75 MHz, $CDCl_3$) δ 177.6, 147.0, 138.3, 128.2, 123.1, 54.8, 44.4, 43.3, 29.9, 21.4. **IR** (ATR, cm^{-1}): 3208, 3098, 3024, 2960, 2921, 2868, 1695. **HRMS** (ESI) m/z: $[M + H]^+$ Calcd for $C_{13}H_{18}NO$ 204.1383; Found 204.1386. **MP**: 150 – 152 ºC. The e.r. was determined by HPLC (ID-3, *i*-PrOH/n-hexane = 10/90, flow rate = 1.0 mL/min, l = 254 nm) tR = 10.3 min (major), 12.9 min (minor). **$[\alpha]_D^{20}$** = +11.8 (*c* = 1.0, $CHCl_3$).

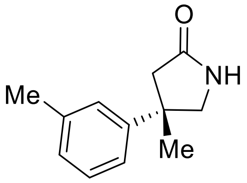

3e

(*S*)-4-methyl-4-(m-tolyl)pyrrolidin-2-one (3e)

Following the *general procedure D*, using 3-methyl-3-(m-tolyl)cyclobutan-1-one **1e** (17.4 mg, 0.10 mmol), arylsulfonylhydroxylamine **2d** (35.9 mg, 0.12 mmol) and catalyst **(*R*)-5b** (8.8 mg, 0.01 mmol) in a 1:1 mixture of DCM/DCE (0.5 mL), **3e** (13.8 mg, 0.073 mmol) was isolated by flash chromatography (petroleum ether/EtOAc gradient 1:1 to 5% MeOH in EtOAc) as a white solid. Yield: 73%. e.r.: 9.5:90.5. Rf (EtOAc): 0.62. **^{1}H NMR** (300 MHz, $CDCl_3$) δ 7.28 – 7.20 (m, 1H), 7.11 – 7.04 (m, 1H), 7.04 – 6.98 (m, 2H), 5.82 (s, 1H), 3.67 (d, *J* = 9.3 Hz, 1H), 3.48 (d, *J* = 9.3 Hz, 1H), 2.78 (d, *J* = 16.3 Hz, 1H), 2.44 (d, *J* = 16.3 Hz, 1H), 2.36 (s, 3H), 1.50 (s, 3H). **^{13}C NMR** (75 MHz, $CDCl_3$) δ 177.4, 146.9, 138.4, 128.6, 127.4, 126.1, 122.3, 54.7, 44.3, 43.4, 29.8, 21.6. **IR** (ATR, cm^{-1}): 3211, 3102, 3020, 2960, 2925, 2868, 1696, 1684, 786, 764, 751, 707. **HRMS** (ESI) m/z: $[M + H]^+$ Calcd for $C_{12}H_{16}NO$ 190.1226; Found 190.1229. **MP**: 114 – 116 ºC. The e.r. was determined by HPLC (OD-3, *i*-PrOH/n-hexane = 10/90, flow rate = 1.0 mL/min, l = 254 nm) tR = 11.6 min (minor), 12.7 min (major). **$[\alpha]_D^{20}$ =** +14.5 (*c* = 1.0, $CHCl_3$).

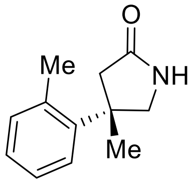

3f

(*S*)-4-methyl-4-(o-tolyl)pyrrolidin-2-one (3f)

Following the *general procedure D*, using 3-methyl-3-(m-tolyl)cyclobutan-1-one **1f** (17.4 mg, 0.10 mmol), arylsulfonylhydroxylamine **2d** (35.9 mg, 0.12 mmol) and catalyst **(*R*)-5b** (8.8 mg, 0.01 mmol) in a 1:1 mixture of DCM/DCE (0.5 mL), **3f** (2.6 mg, 0.014 mmol) was isolated by flash chromatography (petroleum ether/EtOAc gradient 1:1 to 5% MeOH in EtOAc) as a white solid. Yield: 14%. e.r.: 74.5:25.5. Rf (EtOAc): 0.59. **^{1}H NMR** (300 MHz, $CDCl_3$) δ 7.23 – 7.03 (m, 4H), 6.65 (s, 1H), 3.79 (d, *J* = 9.3 Hz, 1H), 3.64 (dd, *J* = 9.4, 1.6 Hz, 1H), 2.86 (d, *J* = 16.1 Hz, 1H), 2.59 (d, *J* = 16.1 Hz, 1H), 2.38 (s, 3H), 1.50 (s, 3H). **^{13}C NMR** ^{13}C NMR (75 MHz, $CDCl_3$) δ 177.2, 144.5, 135.2, 132.5, 126.8, 126.4, 126.3, 54.6, 45.1, 44.7, 27.8, 21.9. **IR** (ATR, cm^{-1}): 3225, 3109, 3062, 3020, 2964, 2928, 2872, 1695, 758, 727. **HRMS** (ESI) m/z: $[M + H]^+$ Calcd for $C_{12}H_{15}NO$ 190.1226; Found 190.1229. **MP**: 116 – 118 ºC. The e.r. was determined by HPLC (OD-3, *i*-PrOH/n-hexane = 10/90, flow rate = 1.0 mL/min, l = 254 nm) tR = 21.1 min (major), 27.9 min (minor). **$[\alpha]_D^{20}$** = +5.4 (*c* = 1.0, $CHCl_3$).

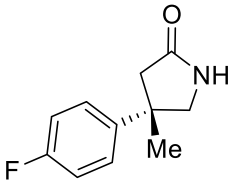

3g

(*S*)-4-(4-fluorophenyl)-4-methylpyrrolidin-2-one (3g)

Following the *general procedure D*, using 3-(4-fluorophenyl)-3-methylcyclobutan-1-one **1g** (17.8 mg, 0.10 mmol), arylsulfonylhydroxylamine **2d** (35.9 mg, 0.12 mmol) and catalyst **(*R*)-5b** (8.8 mg, 0.01 mmol) in a 1:1 mixture of DCM/DCE (0.5 mL), **3g** (13.7 mg, 0.071 mmol) was isolated by flash chromatography (petroleum ether/EtOAc gradient 1:1 to 5% MeOH in EtOAc) as a white solid. Yield: 71%. e.r.: 87:13. Rf

(EtOAc): 0.46. **^{1}H NMR** (300 MHz, $CDCl_3$) δ 7.18 (dd, *J* = 8.6, 5.4 Hz, 2H), 7.03 (dd, *J* = 8.6, 8.6 Hz, 2H), 6.55 (s, 1H), 3.63 (d, *J* = 9.4 Hz, 1H), 3.48 (d, *J* = 9.4 Hz, 1H), 2.74 (d, *J* = 16.3 Hz, 1H), 2.44 (d, *J* = 16.3 Hz, 1H), 1.49 (s, 3H). **^{13}C NMR** (75 MHz, $CDCl_3$) δ 177.2, 163.1, 159.8, 142.7, 142.6, 127.0, 126.8, 115.7, 115.4, 77.5, 77.3, 77.0, 76.6, 54.8, 44.5, 43.0, 29.8. **IR** (ATR, cm^{-1}): 3233, 3105, 3048, 2967, 2925, 2875, 1689, 1512, 834. **^{19}F NMR** (282 MHz, $CDCl_3$) δ -116.2. **HRMS** (ESI) m/z: $[M + H]^+$ Calcd for $C_{11}H_{13}FNO$ 194.0976; Found 194.0979. **MP**: 108 – 110 ºC. The e.r. was determined by HPLC (ID-3, *i*-PrOH/n-hexane = 10/90, flow rate = 1.0 mL/min, l = 254 nm) tR = 14.8 min (major), 20.4 min (minor). **$[\alpha]_D^{20}$ =** +31.8 (*c* = 1.0, $CHCl_3$).

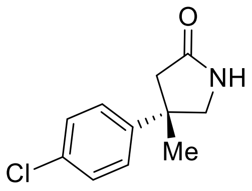

3h

(*S*)-4-(4-chlorophenyl)-4-methylpyrrolidin-2-one (3h)

Following the *general procedure D*, using 3-(4-chlorophenyl)-3-methylcyclobutan-1-one **1h** (19.5 mg, 0.10 mmol), arylsulfonylhydroxylamine **2d** (35.9 mg, 0.12 mmol) and catalyst **(*R*)-5b** (8.8 mg, 0.01 mmol) in a 1:1 mixture of DCM/DCE (0.5 mL), **3h** (12.8 mg, 0.061 mmol) was isolated by flash chromatography (petroleum ether/EtOAc gradient 1:1 to 5% MeOH in EtOAc) as a white solid. Yield: 61%. e.r.: 88.5:11.5. Rf (EtOAc): 0.52. **^{1}H NMR** (300 MHz, $CDCl_3$) δ 7.35 – 7.27 (m, 2H), 7.18 – 7.10 (m, 2H), 6.72 (s, 1H), 3.63 (d, *J* = 9.5 Hz, 1H), 3.47 (d, *J* = 9.5 Hz, 1H), 2.73 (d, *J* = 16.3 Hz, 1H), 2.44 (d, *J* = 16.3 Hz, 1H), 1.48 (s, 3H). **^{13}C NMR** (75 MHz, $CDCl_3$) δ 177.2, 145.4, 132.5, 128.9, 126.8, 54.7, 44.3, 43.1, 29.6. **IR** (ATR, cm^{-1}): 3222, 3098, 2964, 2925, 2872, 1689, 1492, 1092, 750. **HRMS** (ESI) m/z: $[M + H]^+$ Calcd for $C_{11}H_{13}ClNO$ 210.0680; Found 210.0685. **MP**: 111 – 113 ºC. The e.r. was determined by HPLC (ID-3, *i*-PrOH/n-hexane = 10/90, flow rate = 1.0 mL/min, l = 254 nm) tR = 14.6 min (major), 21.0 min (minor). **$[\alpha]_D^{20}$ =** +27.8 (*c* = 1.0, $CHCl_3$).

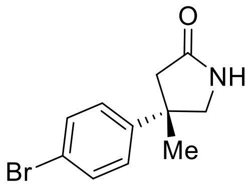

3i

(*S*)-4-(4-bromophenyl)-4-methylpyrrolidin-2-one (3i)

Following the *general procedure D*, using 3-(4-bromophenyl)-3-methylcyclobutan-1-one **1i** (23.9 mg, 0.10 mmol), arylsulfonylhydroxylamine **2d** (35.9 mg, 0.12 mmol) and catalyst **(*R*)-5b** (8.8 mg, 0.01 mmol) in a 1:1 mixture of DCM/DCE (0.5 mL), **3i** (21.3 mg, 0.084 mmol) was isolated by flash chromatography (petroleum ether/EtOAc gradient 1:1 to 5% MeOH in EtOAc) as a white solid. Yield: 84%. e.r.: 87.5:12.5. Rf (EtOAc): 0.55. **^{1}H NMR** (300 MHz, $CDCl_3$) δ 7.49 – 7.44 (m, 2H), 7.11 – 7.07 (m, 2H), 6.50 (s, 1H), 3.63 (d, *J* = 9.4 Hz, 1H), 3.47 (d, *J* = 9.4 Hz, 1H), 2.73 (d, *J* = 16.3 Hz, 1H), 2.44 (d, *J* = 16.3 Hz, 1H), 1.48 (s, 3H). **^{13}C NMR** (75 MHz, $CDCl_3$) δ 177.1, 145.9, 131.8, 127.1, 120.5, 54.6, 44.2, 43.2, 29.6. **IR** (ATR, cm^{-1}): 3225, 2964, 2925, 2875, 1689, 1684, 1008, 821, 764, 750. **HRMS** (ESI) m/z: $[M + H]^+$ Calcd for $C_{11}H_{13}BrNO$ 254.0175; Found 254.0180. **MP**: 116 – 118 ºC. The e.r. was determined by HPLC (OZ-3, *i*-PrOH/n-hexane = 10/90, flow rate = 1.0 mL/min, l = 254 nm) tR = 29.8 min (minor), 42.3 min (major). **$[\alpha]_D^{20}$ =** +24.7 (*c* = 1.0, $CHCl_3$).

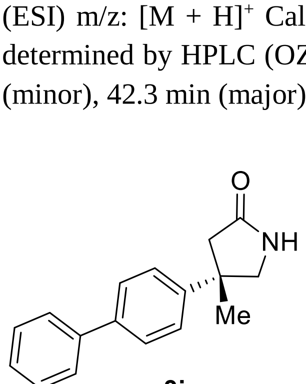

3j

(*S*)-4-([1,1'-biphenyl]-4-yl)-4-methylpyrrolidin-2-one (3j)

Following the *general procedure D*, using 3-([1,1'-biphenyl]-4-yl)-3-methylcyclobutan-1-one **1j** (23.6 mg, 0.10 mmol), arylsulfonylhydroxylamine **2d** (35.9 mg, 0.12 mmol) and catalyst **(*R*)-5b** (8.8 mg, 0.01 mmol) in a 1:1 mixture of DCM/DCE (0.5 mL), **3j** (19.4 mg, 0.077 mmol) was isolated by flash chromatography (petroleum ether/EtOAc gradient 1:1 to 5% MeOH in EtOAc) as a white solid. Yield: 77%. e.r.: 93.5:6.5. For the 1 mmol scale reaction; using 3-([1,1'-biphenyl]-4-yl)-3-methylcyclobutan-1-one **1j** (236.0 mg, 1.0 mmol), arylsulfonylhydroxylamine **2d** (359.0 mg, 1.2 mmol) and catalyst **(*R*)-5b** (88.0 mg, 0.1 mmol) in a 1:1 mixture of DCM/DCE (5.0 mL), **3j** (210.9 mg, 0.839 mmol) was isolated by flash chromatography (petroleum ether/EtOAc gradient 1:1 to 5% MeOH in EtOAc) as a white solid. Yield: 84%. e.r.: 93:7. Rf (EtOAc): 0.54. **^{1}H NMR** (300 MHz, $CDCl_3$) δ 7.65 – 7.53 (m, 4H), 7.49 – 7.40 (m, 2H), 7.38 – 7.34 (m, 1H), 7.33 – 7.27 (m, 2H), 6.23 (s, 1H), 3.72 (d, *J* = 9.2 Hz, 1H), 3.53 (d, *J* = 8.1 Hz, 1H), 2.83 (d, *J* = 16.0 Hz, 1H), 2.50 (d, *J* = 16.0 Hz, 1H), 1.55 (s, 3H). **^{13}C**

NMR (75 MHz, $CDCl_3$) δ 177.2, 145.9, 140.5, 139.7, 128.8, 127.5, 127.4, 127.0, 125.8, 54.7, 44.3, 43.3, 29.7. **IR** (ATR, cm^{-1}): 3218, 2969, 2964, 2921, 2872, 2854, 1700, 1654, 764, 700. **HRMS** (ESI) m/z: $[M + H]^+$ Calcd for $C_{17}H_{18}NO$ 252.1383; Found 252.1389. **MP**: 191 – 193 ºC. The e.r. was determined by HPLC (OD-3, *i*-PrOH/n-hexane = 10/90, flow rate = 1.0 mL/min, l = 254 nm) tR = 23.4 min (minor), 26.1 min (major). $[\alpha]_D^{20}$ = +49.3 (*c* = 1.0, $CHCl_3$).

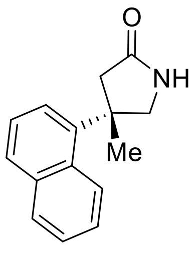

3k

(*S*)-4-methyl-4-(naphthalen-1-yl)pyrrolidin-2-one (3k)

Following the *general procedure A*, using 3-methyl-3-(naphthalen-1-yl)cyclobutan-1-one **1k** (21.0 mg, 0.10 mmol), arylsulfonylhydroxylamine **2d** (35.9 mg, 0.12 mmol) and catalyst **(*R*)-5b** (8.8 mg, 0.01 mmol) in a 1:1 mixture of DCM/DCE (0.5 mL), **3k** (13.3 mg, 0.059 mmol) was isolated by flash chromatography (petroleum ether/EtOAc gradient 1:1 to 5% MeOH in EtOAc) as an orange oil. Yield: 59%. e.r.: 80.5:19.5. Rf (EtOAc): 0.59. **^{1}H NMR** (300 MHz, $CDCl_3$) δ 8.02 – 7.85 (m, 2H), 7.78 (d, *J* = 8.0 Hz, 1H), 7.56 – 7.47 (m, 2H), 7.44 (dd, *J* = 7.7, 7.7 Hz, 1H), 7.36 (dd, *J* = 7.4, 1.4 Hz, 1H), 6.63 (s, 1H), 4.02 (d, *J* = 9.6 Hz, 1H), 3.79 (dd, *J* = 9.6, 1.5 Hz, 1H), 3.14 (d, *J* = 16.6 Hz, 1H), 2.70 (d, *J* = 16.6 Hz, 1H), 1.77 (s, 3H). **^{13}C NMR** (75 MHz, $CDCl_3$) δ 177.0, 142.0, 134.8, 130.9, 129.9, 128.3, 125.9, 125.4, 125.2, 124.7, 124.1, 55.3, 45.8, 44.1, 29.3. **IR** (ATR, cm^{-1}): 3218, 3102, 3052, 2964, 2928, 2872, 1690, 776, 731. **HRMS** (ESI) m/z: $[M + H]^+$ Calcd for $C_{15}H_{16}NO$ 226.1226; Found 226.1132. The e.r. was determined by HPLC (ID-3, *i*-PrOH/n-hexane = 10/90, flow rate = 1.0 mL/min, l = 254 nm) tR = 20.3 min (major), 38.6 min (minor). $[\alpha]_D^{20}$ = +8.4 (*c* = 1.0, $CHCl_3$).

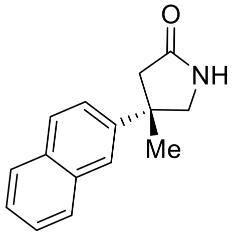

3l

(*S*)-4-methyl-4-(naphthalen-2-yl)pyrrolidin-2-one (3l)

Following the *general procedure D*, using 3-methyl-3-(naphthalen-2-yl)cyclobutan-1-one **1l** (21.0 mg, 0.10 mmol), arylsulfonylhydroxylamine **2d** (35.9 mg, 0.12 mmol) and catalyst **(*R*)-5b** (8.8 mg, 0.01 mmol) in a 1:1 mixture of DCM/DCE (0.5 mL), **3l** (13.3 mg, 0.06 mmol) was isolated by flash chromatography (petroleum ether/EtOAc gradient 1:1 to 5% MeOH in EtOAc) as a white solid. Yield: 60%. e.r.: 19.5:80.5. Rf (EtOAc): 0.53. **^{1}H NMR** (300 MHz, $CDCl_3$) δ 7.89 – 7.76 (m, 3H), 7.63 (d, *J* = 2.0 Hz, 1H), 7.55 – 7.42 (m, 2H), 7.36 (dd, *J* = 8.6, 2.0 Hz, 1H), 6.46 (s, 1H), 3.79 (d, *J* = 9.4 Hz, 1H), 3.58 (dd, *J* = 9.4, 1.0 Hz, 1H), 2.91 (d, *J* = 16.3 Hz, 1H), 2.56 (d, *J* = 16.3 Hz, 1H), 1.60 (s, 3H). **^{13}C NMR** (75 MHz, $CDCl_3$) δ 177.3, 144.1, 133.3, 132.1, 128.7, 127.9, 127.5, 126.4, 126.0, 124.0, 123.5, 54.8, 44.4, 43.6, 29.6. **IR** (ATR, cm^{-1}): 3222, 3098, 3056, 2960, 2925, 2872, 1690, 764, 749. **HRMS** (ESI) m/z: $[M + H]^+$ Calcd for $C_{15}H_{16}NO$ 226.1226; Found 226.1229. **MP**: 170 – 172 ºC. The e.r. was determined by HPLC (OZ-3, *i*-PrOH/n-hexane = 10/90, flow rate = 1.0 mL/min, l = 254 nm) tR = 35.0 min (minor), 39.9 min (major). $[\alpha]_D^{20}$ = +26.4 (*c* = 1.0, $CHCl_3$).

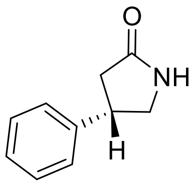

3m

(*S*)-4-phenylpyrrolidin-2-one (3m)

Following the *general procedure D*, using 3-phenylcyclobutan-1-one **1m** (14.0 mg, 0.1 mmol), arylsulfonylhydroxylamine **2d** (35.9 mg, 0.12 mmol) and catalyst **(*R*)-5b** (8.8 mg, 0.01 mmol) in a 1:1 mixture of DCM/DCE (0.5 mL), **3m** (12.0 mg, 0.074 mmol) was isolated by flash chromatography (petroleum ether/EtOAc gradient 1:1 to 5% MeOH in EtOAc) as a white solid. Yield: 74%. e.r.: 52.5:47.5. Rf (EtOAc): 0.53. **^{1}H NMR** (300 MHz, $CDCl_3$) δ 7.40 – 7.21 (m, 5H), 6.46 (s, 1H), 3.79 (t, *J* = 8.6 Hz, 1H), 3.75 – 3.63 (m, 1H), 3.43 (dd, *J* = 8.9, 6.9 Hz, 1H), 2.74 (dd, *J* = 16.9, 8.7 Hz, 1H), 2.51 (dd, *J* = 16.9, 8.6 Hz, 1H).**^{13}C NMR** (75 MHz, $CDCl_3$) δ 177.7, 142.1, 128.9, 127.1, 126.8, 49.5, 40.3, 37.9. **IR** (ATR, cm^{-1}): 3233, 3094, 3059, 3027, 2932, 2875, 1684, 751, 699. **HRMS** (ESI) m/z: $[M + H]^+$ Calcd for $C_{10}H_{12}NO$ 162.0297; Found 162.0913. Yield: 84% **MP**: 99 – 101 ºC. The e.r. was determined by HPLC (IF-3, *i*-PrOH/n-hexane = 10/90, flow rate = 1.0 mL/min, l = 254 nm) tR = 17.9 min (minor), 18.9 min (major). $[\alpha]_D^{20}$ = +2.2 (*c* = 1.0, $CHCl_3$).

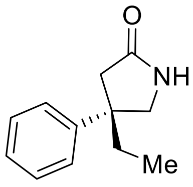

3n

(*S*)-4-ethyl-4-phenylpyrrolidin-2-one (3n)

Following the *general procedure D*, using 3-ethyl-3-phenylcyclobutan-1-one **1n** (17.4 mg, 0.1 mmol), arylsulfonylhydroxylamine **2d** (35.9 mg, 0.12 mmol) and catalyst **(*R*)-5b** (8.8 mg, 0.01 mmol) in a 1:1 mixture of DCM/DCE (0.5 mL), **3n** (10.5 mg, 0.056 mmol) was isolated by flash chromatography (petroleum ether/EtOAc gradient 1:1 to 5% MeOH in EtOAc) as a colourless oil. Yield: 56%. e.r.: 85:15. Rf (EtOAc): 0.58. **^{1}H NMR** (300 MHz, $CDCl_3$) δ 7.38 – 7.30 (m, 2H), 7.24 (dd, *J* = 7.3, 2.3 Hz, 1H), 7.17 – 7.07 (m, 2H), 6.32 (s, 1H), 3.66 (d, *J* = 9.5 Hz, 1H), 3.59 (d, *J* = 9.5 Hz, 1H), 2.75 (d, *J* = 16.4 Hz, 1H), 2.56 (d, *J* = 16.4 Hz, 1H), 1.82 (q, *J* = 7.4 Hz, 2H), 0.72 (t, *J* = 7.4 Hz, 3H). **^{13}C NMR** (75 MHz, $CDCl_3$) δ 177.4, 145.1, 128.5, 126.6, 126.1, 52.7, 47.5, 41.5, 34.5, 8.9. **IR** (ATR, cm^{-1}): 3235, 3088, 3060, 3031, 2970, 2934, 2880, 2765, 1683, 761, 700. **HRMS** (ESI) m/z: $[M + H]^+$ Calcd for $C_{12}H_{16}NO$ 190.1226; Found 190.1231. The e.r. was determined by HPLC (IF-3, *i*-PrOH/n-hexane = 10/90, flow rate = 1.0 mL/min, l = 254 nm) tR = 15.0 min (major), 16.3 min (minor). **$[\alpha]_D^{20}$ =** +19.9 (*c* = 1.0, $CHCl_3$).

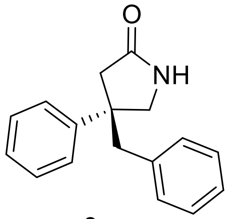

3o

(*S*)-4-benzyl-4-phenylpyrrolidin-2-one (3o)

Following the *general procedure D*, using 3-benzyl-3-phenylcyclobutan-1-one **1o** (23.6 mg, 0.1 mmol), arylsulfonylhydroxylamine **2d** (35.9 mg, 0.12 mmol) and catalyst **(*R*)-5b** (8.8 mg, 0.01 mmol) in a 1:1 mixture of DCM/DCE (0.5 mL), **3o** (18.5 mg, 0.074 mmol) was isolated by flash chromatography (petroleum ether/EtOAc gradient 1:1 to 5% MeOH in EtOAc) as a white solid. Yield: 74%. e.r.: 86:14. Rf (EtOAc): 0.68. **^{1}H NMR** (300 MHz, $CDCl_3$) δ 7.35 – 7.24 (m, 3H), 7.22 – 7.08 (m, 3H), 6.96 – 6.81 (m, 2H), 6.68 (s, 1H), 6.66 – 6.60 (m, 2H), 3.74 (dd, *J* = 9.7, 1.5 Hz, 1H), 3.64 (d, *J* = 9.7 Hz, 1H), 3.07 (d, *J* = 13.2 Hz, 1H), 3.02 (d, *J* = 13.2 Hz, 1H), 2.70 (s, 2H). **^{13}C NMR** (75 MHz, $CDCl_3$) δ 177.5, 144.4, 136.4, 130.6, 128.3, 127.7, 126.8, 126.7, 126.6, 51.8, 48.3, 47.5, 41.6. **IR** (ATR, cm^{-1}): 3232, 3106, 3085, 3060, 3027, 2923, 2873, 1694, 700. **HRMS** (ESI) m/z: $[M + H]^+$ Calcd for $C_{17}H_{18}NO$ 252.1383; Found 252.1388. **MP**: 143 – 145 ºC. The e.r. was determined by HPLC (ID-3, *i*-PrOH/n-hexane = 10/90, flow rate = 1.0 mL/min, l = 254 nm) tR = 17.3 min (major), 22.6 min (minor). **$[\alpha]_D^{20}$ =** -22.9 (*c* = 1.0, $CHCl_3$).

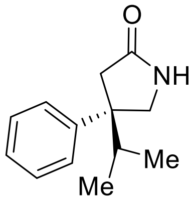

3p

(*R*)-4-isopropyl-4-phenylpyrrolidin-2-one (3p)

Following the *general procedure D*, using 3-isopropyl-3-phenylcyclobutan-1-one **1p** (18.8 mg, 0.1 mmol), arylsulfonylhydroxylamine **2d** (35.9 mg, 0.12 mmol) and catalyst **(*R*)-5b** (8.8 mg, 0.01 mmol) in a 1:1 mixture of DCM/DCE (0.5 mL), **3p** (12.8 mg, 0.063 mmol) was isolated by flash chromatography (petroleum ether/EtOAc gradient 1:1 to 5% MeOH in EtOAc) as a white solid. Yield: 63%. e.r.: 50.5:49.5. Rf (EtOAc): 0.64. **^{1}H NMR** (300 MHz, $CDCl_3$) δ 7.32 (dd, *J* = 8.3, 6.7 Hz, 2H), 7.28 – 7.18 (m, 1H), 7.10 (dd, *J* = 7.2, 1.8 Hz, 2H), 6.36 (s, 1H), 3.74 (d, *J* = 10.2 Hz, 1H), 3.69 (d, *J* = 9.8 Hz, 1H), 2.76 (d, *J* = 16.7 Hz, 1H), 2.65 (d, *J* = 16.7 Hz, 1H), 1.99 (p, *J* = 7.0 Hz, 1H), 0.86 (d, *J* = 6.8 Hz, 3H), 0.78 (d, *J* = 6.8 Hz, 3H).**^{13}C NMR** (75 MHz, $CDCl_3$) δ 177.6, 145.1, 128.1, 126.9, 126.4, 51.2, 50.4, 39.5, 37.4, 18.0. **IR** (ATR, cm^{-1}): 3218, 3098, 3059, 3031, 2964, 2935, 2875, 1689, 766, 749, 734, 701. **HRMS** (ESI) m/z: $[M + H]^+$ Calcd for $C_{13}H_{18}NO$ 204.1383; Found 204.1387. **MP**: 99 – 101 ºC. The e.r. was determined by HPLC (ID-3, *i*-PrOH/n-hexane = 10/90, flow rate = 1.0 mL/min, l = 254 nm) tR = 16.3 min (major), 19.2 min (minor). **$[\alpha]_D^{20}$ =** +38.9 (*c* = 1.0, $CHCl_3$).

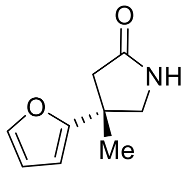

3q

(*R*)-4-(furan-2-yl)-4-methylpyrrolidin-2-one (3q)

Following the *general procedure D*, using 3-(furan-2-yl)-3-methylcyclobutan-1-one **1q** (15.0 mg, 0.1 mmol), arylsulfonylhydroxylamine **2d** (35.9 mg, 0.12 mmol) and catalyst **(*R*)-5b** (8.8 mg, 0.01 mmol) in a 1:1 mixture of DCM/DCE (0.5 mL), **3q** (13.5 mg, 0.082 mmol) was isolated by flash chromatography (petroleum ether/EtOAc gradient 1:1 to 5% MeOH in EtOAc) as an orange oil. Yield: 82%. e.r.: 78:22. Rf (EtOAc): 0.44. **^{1}H NMR** (300 MHz, $CDCl_3$) δ 7.35 (dd, *J* = 1.9, 0.9 Hz, 1H), 6.30 (dd, *J* = 3.2, 1.8 Hz, 1H), 6.11 (dd, *J* = 3.2, 0.8 Hz, 1H), 6.00 (s, 1H), 3.68 (d, *J* = 9.5 Hz, 1H), 3.27 (d, *J* = 9.5 Hz, 1H), 2.81 (d, *J* = 16.6 Hz, 1H), 2.30 (d, *J* = 16.7 Hz, 1H), 1.53 (s, 3H). **^{13}C NMR** (75 MHz, $CDCl_3$) δ 176.6, 158.4, 141.8, 110.1, 104.5, 53.3, 43.0, 39.9, 25.2. **IR** (ATR, cm^{-1}): 3233, 3115, 2971, 2932, 2879, 1684, 734, 680. **HRMS** (ESI) m/z: $[M + H]^+$ Calcd for $C_9H_{12}NO_2$ 166.0863; Found 166.0863. The e.r. was determined by HPLC (ID-3, *i*-PrOH/n-hexane = 10/90, flow rate = 1.0 mL/min, l = 254 nm) tR = 16.7 min (major), 19.9 min (minor). **$[\alpha]_D^{20}$ =** +20.9 (*c* = 1.0, $CHCl_3$).

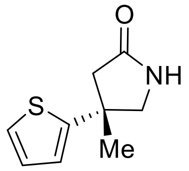

3r

(*R*)-4-(tiophen-2-yl)-4-methylpyrrolidin-2-one (3r)

Following the *general procedure D*, using 3-(tiophen-2-yl)-3-methylcyclobutan-1-one **1r** (15.0 mg, 0.1 mmol), arylsulfonylhydroxylamine **2d** (35.9 mg, 0.12 mmol) and catalyst **(*R*)-5b** (8.8 mg, 0.01 mmol) in a 1:1 mixture of DCM/DCE (0.5 mL), **3r** (9.6 mg, 0.053 mmol) was isolated by flash chromatography (petroleum ether/EtOAc gradient 1:1 to 5% MeOH in EtOAc) as a white solid. Yield: 53%. e.r.: 72.5:27.5. Rf (EtOAc): 0.59. **^{1}H NMR** (300 MHz, $CDCl_3$) δ 7.19 (dd, *J* = 5.1, 1.2 Hz, 1H), 6.95 (dd, *J* = 5.1, 3.6 Hz, 1H), 6.90 (dd, *J* = 3.6, 1.2 Hz, 1H), 6.37 (s, 1H), 3.66 (d, *J* = 9.6 Hz, 1H), 3.43 (d, *J* = 9.6 Hz, 1H), 2.79 (d, *J* = 16.5 Hz, 1H), 2.46 (d, *J* = 16.5 Hz, 1H), 1.62 (s, 3H). **^{13}C NMR** (75 MHz, $CDCl_3$) δ 176.7, 151.3, 126.9, 123.6, 122.8, 56.4, 46.4, 41.6, 28.7. **IR** (ATR, cm^{-1}): 3186, 3088, 2967, 2928, 2868, 1684, 764, 750, 698. **HRMS** (ESI) m/z: $[M + H]^+$ Calcd for $C_9H_{12}NOS$ 182.0634; Found 182.0635. **MP**: 109 – 111 ºC. The e.r. was determined by HPLC (IF-3, *i*-PrOH/n-hexane = 10/90, flow rate = 1.0 mL/min, l = 254 nm) tR = 17.8 min (minor), 21.2 min (major). **$[\alpha]_D^{20}$ =** +28.4 (*c* = 1.0, $CHCl_3$).

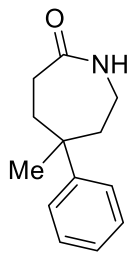

3s

5-methyl-5-phenylazepan-2-one (3s)

Following the *general procedure D*, using 4-methyl-4-phenylcyclohexan-1-one **1s** (18.8 mg, 0.1 mmol), arylsulfonylhydroxylamine **2d** (35.9 mg, 0.12 mmol) and catalyst **(*R*)-5b** (8.8 mg, 0.01 mmol) in a 1:1 mixture of DCM/DCE (0.5 mL), **3s** (11.2 mg, 0.055 mmol) was isolated by flash chromatography (petroleum ether/EtOAc gradient 1:1 to 5% MeOH in EtOAc) as a white solid. Yield: 55%. e.r.: 50:50. Rf (EtOAc): 0.31. **^{1}H NMR** (300 MHz, $CDCl_3$) δ 7.41 – 7.28 (m, 4H), 7.26 – 7.18 (m, 1H), 6.37 (s, 1H), 3.20 (td, J = 5.8, 3.6 Hz, 2H), 2.48 – 2.31 (m, 4H), 1.88 – 1.63 (m, 2H), 1.25 (s, 3H).**^{13}C NMR** (75 MHz, $CDCl_3$) δ 178.8, 146.8, 128.9, 126.2, 126.2, 41.4, 40.8, 38.7, 34.1, 32.4, 32.0. Physical and spectroscopic data are consistent with those reported in the literature.[23] The e.r. was determined by HPLC (ID-3, *i*-PrOH/n-hexane = 10/90, flow rate = 1.0 mL/min, l = 254 nm) tR = 25.8 min (major), 30.9 min (minor).

[23] Aube, J.; Wang, Y.; Hammond, M.; Tanol, M.; Takusagawa, F.; Vander Velde, D. Synthetic Aspects of an Asymmetric Nitrogen-Insertion Process: Preparation of Chiral, Non-Racemic Caprolactams and Valerolactams; Total Synthesis of (−)-Alloyohimbane. *J. Am. Chem. Soc.* **1990**, *112*, 4879–4891.

3.5. Derivatization of 3-substituted-γ-lactam adducts 3a and 3j. Compounds 6a-9j''.

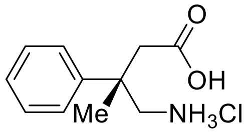

6a

(*S*)-4-amino-3-methyl-3-phenylbutanoic acid hydrochloride (6a)

A suspension of lactam **3a** (35 mg, 0.20 mmol) in aqueous 6 M HCl (4 mL) was heated to 100 °C for 24 h. The homogeneous mixture was cooled to room temperature, diluted with 10 mL of H_2O and washed with 2 mL of CH_2Cl_2. The aqueous layer was concentrated by azeotropic distillation with toluene obtaining, without further purification, the product as a white solid (45.1 mg, 0.196 mmol). Yield: 98%. Rf (EtOAc/MeOH 9:1): 0.18. **^{1}H NMR** (300 MHz, Deuterium Oxide) δ 7.64 – 7.27 (m, 4H), 3.52 (d, *J* = 13.2 Hz, 1H), 3.37 (d, *J* = 13.0 Hz, 1H), 2.94 (d, *J* = 15.1 Hz, 1H), 2.84 (d, *J* = 15.1 Hz, 1H), 1.62 (s, 3H). **^{13}C NMR** (75 MHz, Deuterium Oxide) δ 175.7, 141.4, 129.2, 127.7, 126.3, 49.1, 45.2, 22.3. **IR** (ATR, cm^{-1}): 3135, 3024, 2963, 2933, 1703, 1598, 1048, 756, 700. **HRMS** (ESI) m/z: $[M - Cl]^+$ Calcd for $C_{11}H_{16}NO_2$ 194.1176; Found 194.1182. **MP**: 185 – 187 °C. $[\alpha]_D^{20}$ = -78.2 (*c* = 1.0, H_2O).

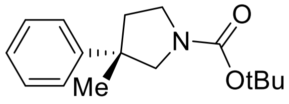

7a

***tert*-Butyl (*S*)-3-methyl-3-phenylpyrrolidine-1-carboxylate (7a)**

A solution of $LiAlH_4$ 23 mg, 0.60 mmol, 3 equiv.) in THF (2 mL, 0.3 M for $LiAlH_4$) was added at room temperature over a solution of lactam **3a** (35 mg, 0.20 mmol) in THF (1 mL, 0.2 M for lactam **3a**). The mixture was heated to 70 °C for 16 h. After cooling to room temperature, 1 mL of NaOH 2 M followed by 1 mL of H_2O, were added to the reaction mixture, and was stirred for 30 min. Then di-*tert*-butyl dicarbonate (130 mg, 0.60 mmol, 3 equiv.) were added and the mixture was stirred at room temperature for 16 h. The mixture was diluted with EtOAc, the layers were separated and the aqueous layer was extracted with EtOAc (3 x 10 mL). The combined organic layers were dried over anhydrous Na_2SO_4, filtered and evaporated. The product was isolated by flash column chromatography (petroleum ether/EtOAc 19:1) as a colourless oil, (45.3 mg, 0.173 mmol). Yield: 87%. e.r.: 85.5:14.5. Rf (PE/EtOAc 19:1): 0.65. **^{1}H NMR** (300 MHz, $CDCl_3$) (* indicates signals of rotamer) δ 7.33 (dd, *J* = 8.4, 7.0 Hz, 2H), 7.27 – 7.18 (m, 3H), 3.70 – 3.37 (m, 4H), 2.27 – 1.94 (m, 2H), 1.49 (s, 9H), 1.48* (s, 9H), 1.36 (s, 3H), 1.35* (s, 3H). **^{13}C NMR** (75 MHz, $CDCl_3$) δ 155.0, 147.3, 128.6, 126.4, 126.4, 125.8, 125.7, 79.4, 79.3, 57.7, 56.9, 45.9, 45.1, 45.0, 44.7, 38.0, 37.1, 28.7, 27.9, 27.8. **IR** (ATR, cm^{-1}): 2966, 2934, 2876, 1693, 1395, 1364, 1157, 1104, 764, 699. **HRMS** (ESI) m/z: $[M + Na]^+$ Calcd for $C_{16}H_{23}NO_2Na$ 284.1621; Found 284.1631. The e.r. was determined by HPLC (OD-3, *i*-PrOH/n-hexane = 10/90, flow rate = 1.0 mL/min, l = 254 nm) tR = 4.2 min (minor), 5.0 min (major). **$[\alpha]_D^{20}$ =** -37.5 (*c* = 1.0, $CHCl_3$).

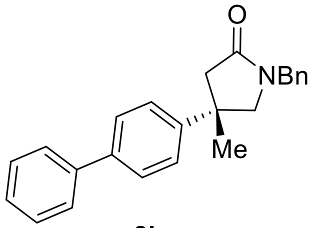

8j

(*S*)-4-([1,1'-biphenyl]-4-yl)-1-benzyl-4-methylpyrrolidin-2-one (8j)

To a solution of NaH (60%, dispersion in mineral oil) (7.2 mg, 0.18 mmol, 1.2 equiv.) in anhydrous THF (0.5 M for NaH) at 0 °C, a solution of lactam **3j** (38 mg, 0.15 mmol) in THF (0.15 M in lactam **3j**) was added. The mixture was stirred at room temperature for 2 h, then benzyl bromide (20 µL, 0.165, 1.1 equiv.) was added. The mixture was stirred at room temperature for additional 2 h while monitoring by TLC The mixture was diluted with H_2O (5 mL), extracted with EtOAc (3 x 10 mL). The combined organic layers were washed with brine (5 mL), dried over anhydrous Na_2SO_4, filtered and evaporated. The product was isolated by flash chromatography (petroleum ether/EtOAc gradient 7:3 to 1:1) as a yellow oil (46.4 mg, 0.136 mmol). Yield: 91%. e.r.: 90.5:9.5. Rf (PE/EtOAc 1:1): 0.75. **^{1}H NMR** (300 MHz, $CDCl_3$) δ 7.61 – 7.53 (m, 4H), 7.49 – 7.42 (m, 2H), 7.40 – 7.21 (m, 8H), 4.63 (d, *J* = 14.7 Hz, 1H), 4.49 (d, *J* = 14.7 Hz, 1H), 3.60 (d, *J* = 9.5 Hz, 1H), 3.36 (d, *J* = 9.5 Hz, 1H), 2.96 (d, *J* = 16.2 Hz, 1H), 2.66 (d, *J* = 16.2 Hz, 1H), 1.46 (s, 3H). **^{13}C NMR** (75 MHz, $CDCl_3$) δ 173.5, 146.0, 140.6, 139.7, 136.4, 128.9, 128.8, 128.3, 127.8, 127.5, 127.4, 127.1, 125.9, 59.1, 46.7, 45.3, 40.3, 30.0. **IR** (ATR, cm^{-1}): 3060, 3027, 2959, 2915, 2861, 1688, 1488, 1426, 1253, 768, 734, 734, 699. **HRMS** (ESI) m/z: $[M + H]^+$ Calcd for $C_{24}H_{24}NO$ 342.1852; Found 342.1846. The e.r. was determined by HPLC (IA,

i-PrOH/n-hexane = 10/90, flow rate = 1.0 mL/min, l = 254 nm) tR = 9.2 min (major), 11.5 min (minor). **[α]**$_D^{20}$ = -51.8 (*c* = 1.0, $CHCl_3$).

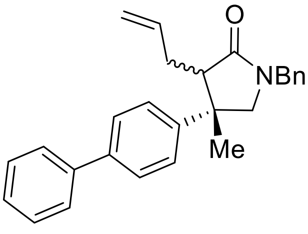

9j

(4*S*)-4-([1,1'-biphenyl]-4-yl)-3-allyl-1-benzyl-4-methylpyrrolidin-2-one (9j)
In a flame-dried round bottom flask, *N*,*N*-diisopropylamine (17 µL, 0.12 mmol, 1.2 equiv.) was dissolved in THF (148 µL, 1 M in *N*,*N*-diisopropylamine). *n*-Buthyllithium (1.6 M in hexanes) (75 µL, 0.119 mmol, 1.19 equiv.) was added and the mixture was stirred for 20 min at 0 ºC. The previous LDA solution was added over a solution of *N*-benzyl lactam **4d** (34 mg, 0.10 mmol) in THF (1 mL, 0.1 M in **4d**) at -78 ºC. The mixture was stirred for 1 h at – 78 ºC, followed by the addition of allyl bromide (12 µL, 0.13 mmol, 1.3 equiv.). The reaction temperature was raised to room temperature and stirred for 16 h. The reaction was quenched with saturated aqueous NH_4Cl solution (2mL) followed by extraction with CH_2Cl_2 (10 mL x 3). The combined organic layers were washed with brine (5 mL), dried over anhydrous Na_2SO_4, filtered and evaporated. The product appeared as a as a mixture of diastereomers which were separately isolated by flash chromatography (petroleum ether/EtOAc 9:1). dr: 1:2.6.

Diastereomer 1: **9j'** (11 mg, 0.029 mmol). White solid. Yield: 29%. e.r.: 90.5:9.5. Rf (PE/EtOAc 9:1): 0.70. **^{1}H NMR** (300 MHz, $CDCl_3$) δ 7.61 – 7.50 (m, 4H), 7.46 – 7.40 (m, 2H), 7.37 – 7.25 (m, 8H), 5.93 (dddd, *J* = 17.1, 10.1, 7.9, 6.0 Hz, 1H), 5.11 (dq, *J* = 17.1, 1.6 Hz, 1H), 5.02 – 4.89 (m, 1H), 4.59 (d, *J* = 14.6 Hz, 1H), 4.46 (d, *J* = 14.6 Hz, 1H), 3.42 (d, *J* = 9.8 Hz, 1H), 3.12 (d, *J* = 9.8 Hz, 1H), 3.01 (t, *J* = 7.0 Hz, 1H), 2.74 – 2.60 (m, 1H), 2.32 (ddd, *J* = 13.5, 7.9, 6.6 Hz, 1H), 1.38 (s, 3H). **^{13}C NMR** (75 MHz, $CDCl_3$) δ 174.9, 144.6, 140.6, 139.7, 137.0, 136.5, 128.9, 128.9, 128.5, 127.8, 127.5, 127.3, 127.1, 126.5, 116.1, 59.6, 51.8, 46.9, 44.0, 30.9, 22.0. **IR** (ATR, cm^{-1}): 3063, 3027, 2970, 2926, 1690, 1487, 1437, 767, 734, 699. **HRMS** (ESI) m/z: $[M + H]^+$ Calcd for $C_{27}H_{28}NO$ 382.2165; Found 382.2166. **MP**: 105 – 107 ºC. The e.r. was determined by HPLC (ASH, *i*-PrOH/n-hexane = 10/90, flow rate = 1.0 mL/min, l = 254 nm) tR = 15.5 min (major), 20.3 min (minor). **[α]**$_D^{20}$ = +5.4 (*c* = 0.3, $CHCl_3$).

Diastereomer 2: **9j''** (23 mg, 0.060 mmol). White solid. Yield: 60%. e.r.: 90.5:9.5. Rf (PE/EtOAc 9:1): 0.50. **^{1}H NMR** (300 MHz, $CDCl_3$) δ 7.62 – 7.54 (m, 4H), 7.53 – 7.48 (m, 2H), 7.47 – 7.40 (m, 2H), 7.39 – 7.30 (m, 6H, C_{13}-H), 7.20 – 7.12 (m, 2H), 5.74 (dddd, *J* = 16.8, 10.1, 7.7, 6.6 Hz, 1H), 4.90 (ddt, *J* = 10.2, 2.1, 1.1 Hz, 1H), 4.82 (dq, *J* = 17.0, 1.6 Hz, 1H), 4.50 (d, *J* = 14.7 Hz, 1H), 4.55 (d, *J* = 14.7 Hz, 1H), 3.69 (d, *J* = 9.7 Hz, 1H), 3.22 (d, *J* = 9.7 Hz, 1H), 2.63 (t, *J* = 6.7 Hz, 1H), 2.21 (dtt, *J* = 14.9, 6.8, 1.4 Hz 1H), 2.11 – 1.97 (m,1H), 1.49 (s, 3H). **^{13}C NMR** (75 MHz, $CDCl_3$) δ 175.6, 142.8, 140.6, 139.5, 136.4, 135.7, 128.9, 128.9, 128.8, 127.9, 127.5, 127.1, 127.1, 126.9, 116.8, 57.2, 54.0, 47.0, 43.7, 32.7, 29.6. **IR** (ATR, cm^{-1}): 3060, 3027, 2966, 2926, 2869, 1686, 1488, 1439, 767, 736, 699 **HRMS** (ESI) m/z: $[M + H]^+$ Calcd for $C_{27}H_{28}NO$ 382.2165; Found 382.2166. **MP**: 105 – 107 ºC. The e.r. was determined by HPLC (OD-3, *i*-PrOH/n-hexane = 10/90, flow rate = 1.0 mL/min, l = 254 nm) tR = 10.1 min (major), 13.9 min (minor). **[α]**$_D^{20}$ = -73.5 (*c* = 1.0, $CHCl_3$).

4. X-Ray Analysis of Compound 3e

X-ray Crystallography Data for **3e** (CCDC 2502384): A suitable crystal for X-ray crystallography was obtained by taking compound **3e** in 1:10 MeOH/hexane (1 mL) and the solution was kept for 7 days at room temperature under air. The solvent was slowly evaporated and crystals were formed. Crystal Data for $C_{12}H_{15}NO$ (M =189.259 g/mol): monoclinic, space group $P2_1$ (no. 4), a = 7.9739(3) Å, b = 6.7390(2) Å, c = 10.2131(4) Å, β = 106.718(4)°, V = 525.62(3) Å^3, Z = 2, T = 170.00(10) K, μ(Cu Kα) = 0.597 mm^{-1}, $Dcalc$ = 1.196 g/cm^3, 5943 reflections measured (9.04° ≤ 2Θ ≤ 147.2°), 1969 unique (R_{int} = 0.0708, R_{sigma} = 0.0655) which were used in all calculations. The final R_1 was 0.0526 (I>=2u(I)) and wR_2 was 0.1447 (all data).

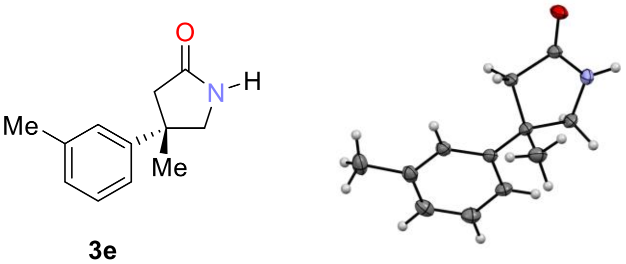

3e

Figure SI-5. Structure and ORTEP of **3e** (ellipsoid contours of probability levels are 50%).

Table SI-2. Crystal data and structure refinement for **3e.**

Identification code	b20240180_AN572DDUCu
Empirical formula	$C_{12}H_{15}NO$
Formula weight	189.259
Temperature/K	170.00(10)
Crystal system	monoclinic
Space group	$P2_1$
a/Å	7.9739(3)
b/Å	6.7390(2)
c/Å	10.2131(4)
α/°	90
β/°	106.718(4)
γ/°	90
Volume/Å^3	525.62(3)
Z	2
ρ_{calc}g/cm^3	1.196
μ/mm^{-1}	0.597
F(000)	204.6
Crystal size/mm^3	0.375 × 0.124 × 0.047
Radiation	Cu Kα (λ = 1.54184)
2Θ range for data collection/°	9.04 to 147.2
Index ranges	$-9 \le h \le 9$, $-7 \le k \le 8$, $-12 \le l \le 12$
Reflections collected	5943
Independent reflections	1969 [R_{int} = 0.0708, R_{sigma} = 0.0655]
Data/restraints/parameters	1969/1/129
Goodness-of-fit on F^2	1.071
Final R indexes [I>=2σ (I)]	R_1 = 0.0526, wR_2 = 0.1400
Final R indexes [all data]	R_1 = 0.0563, wR_2 = 0.1447
Largest diff. peak/hole / e Å^{-3}	0.17/-0.16
Flack parameter	1.7(3)

5. NMR Spectra

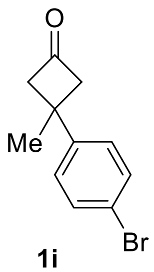

1i

^{1}H-NMR (300 MHz, $CDCl_3$)

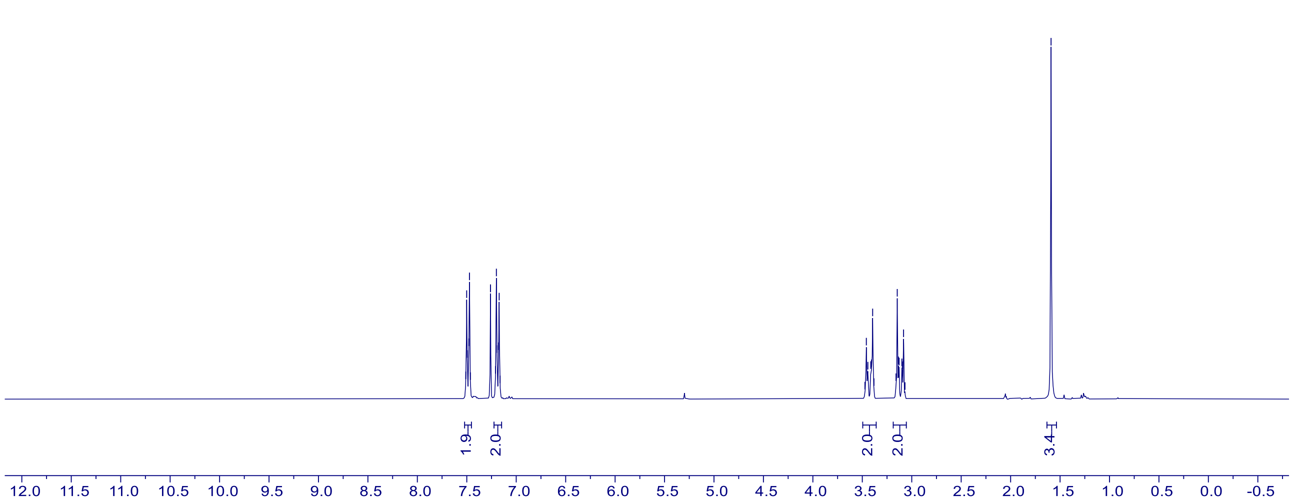

^{13}C-NMR (75 MHz, $CDCl_3$)

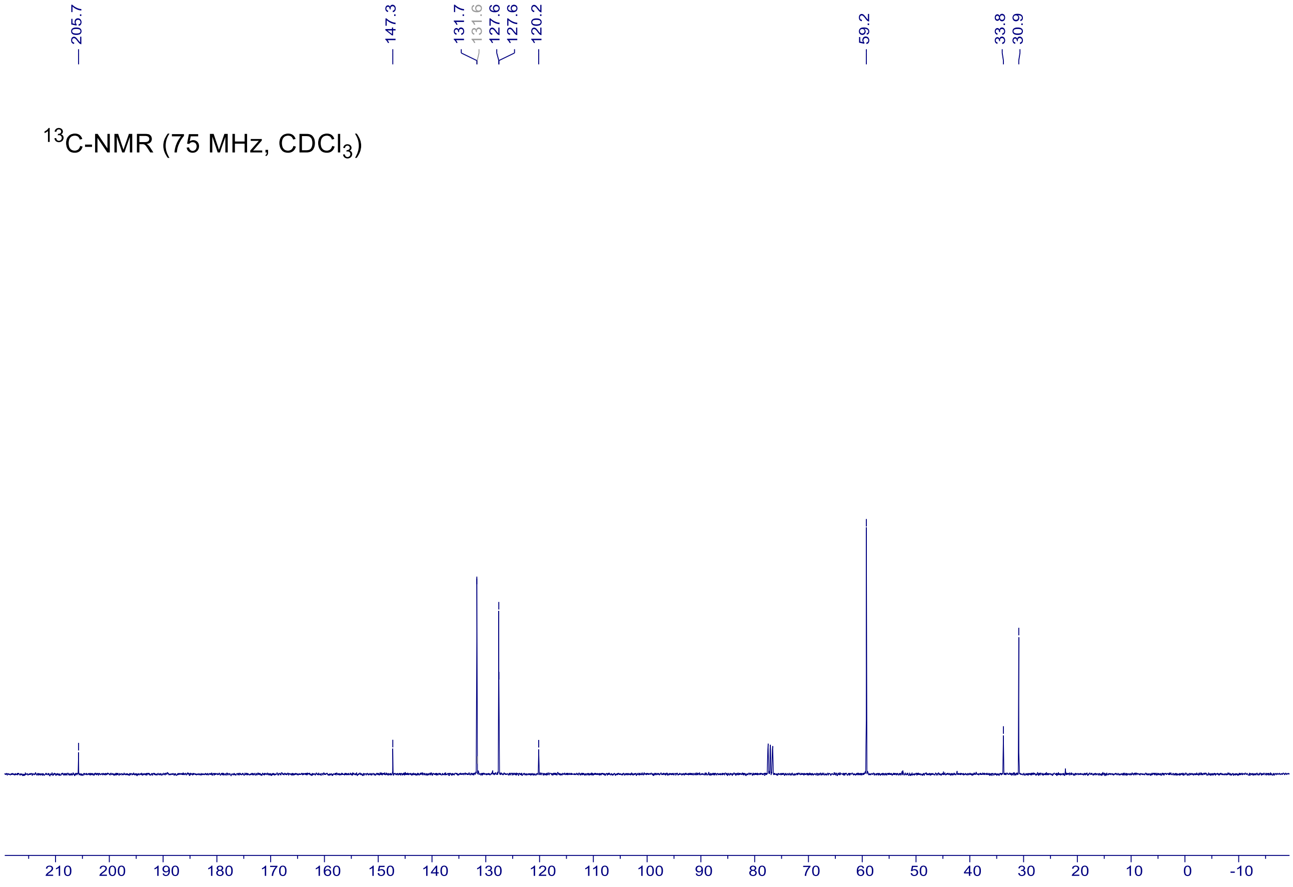

Figure SI-6. NMR spectra of compound **1i.**

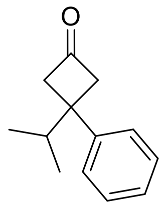

1p

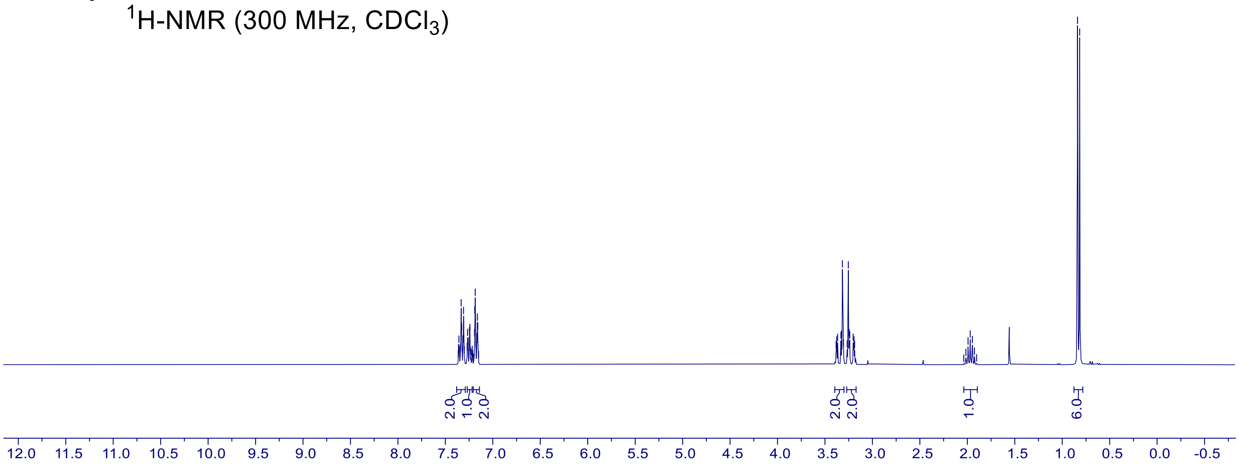

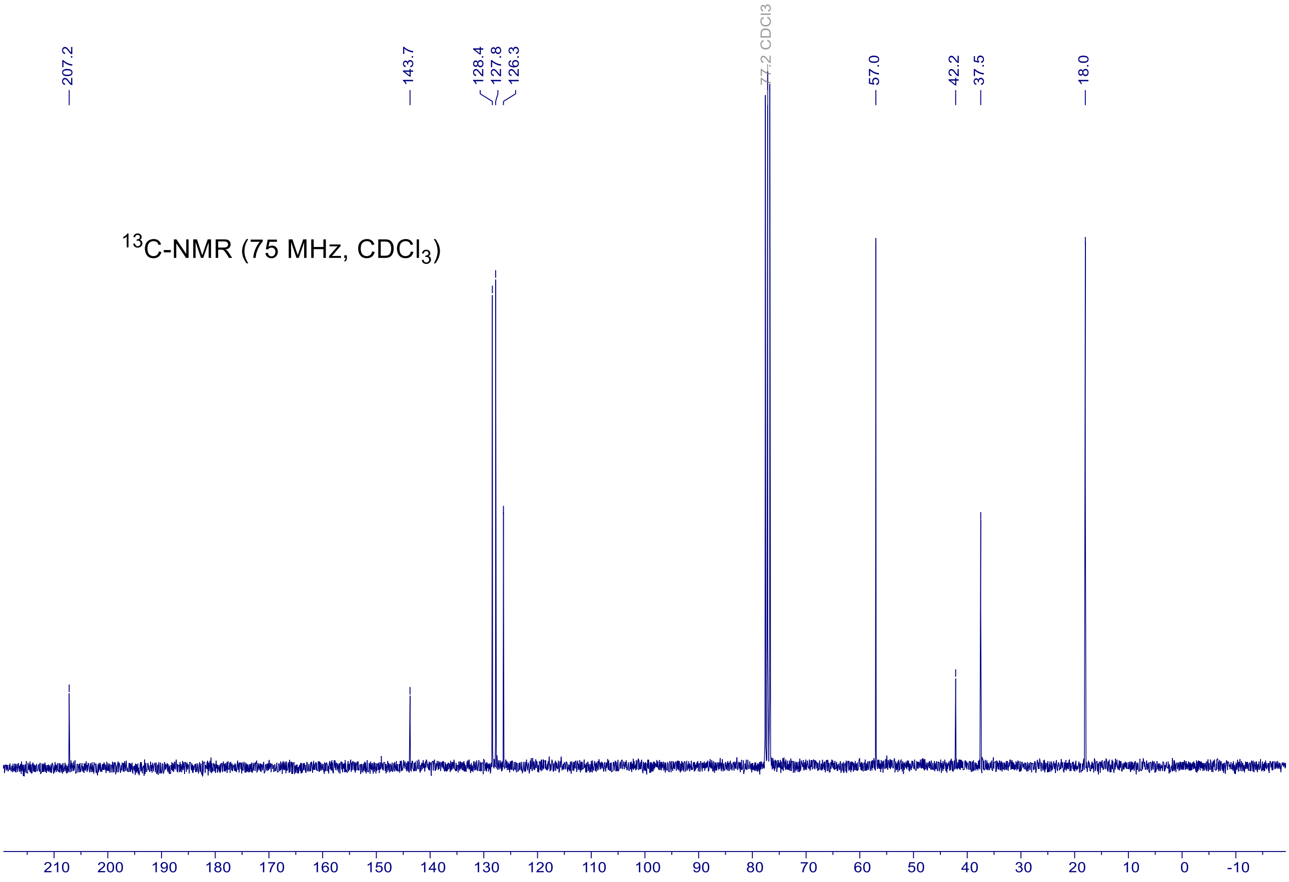

Figure SI-7. NMR spectra of compound **1p.**

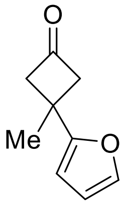

1q

^{1}H-NMR (300 MHz, $CDCl_3$)

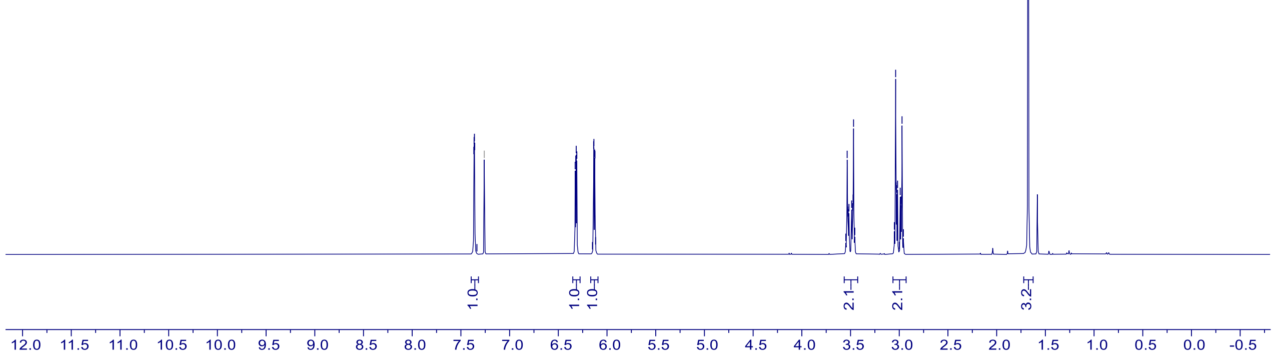

^{13}C-NMR (75 MHz, $CDCl_3$)

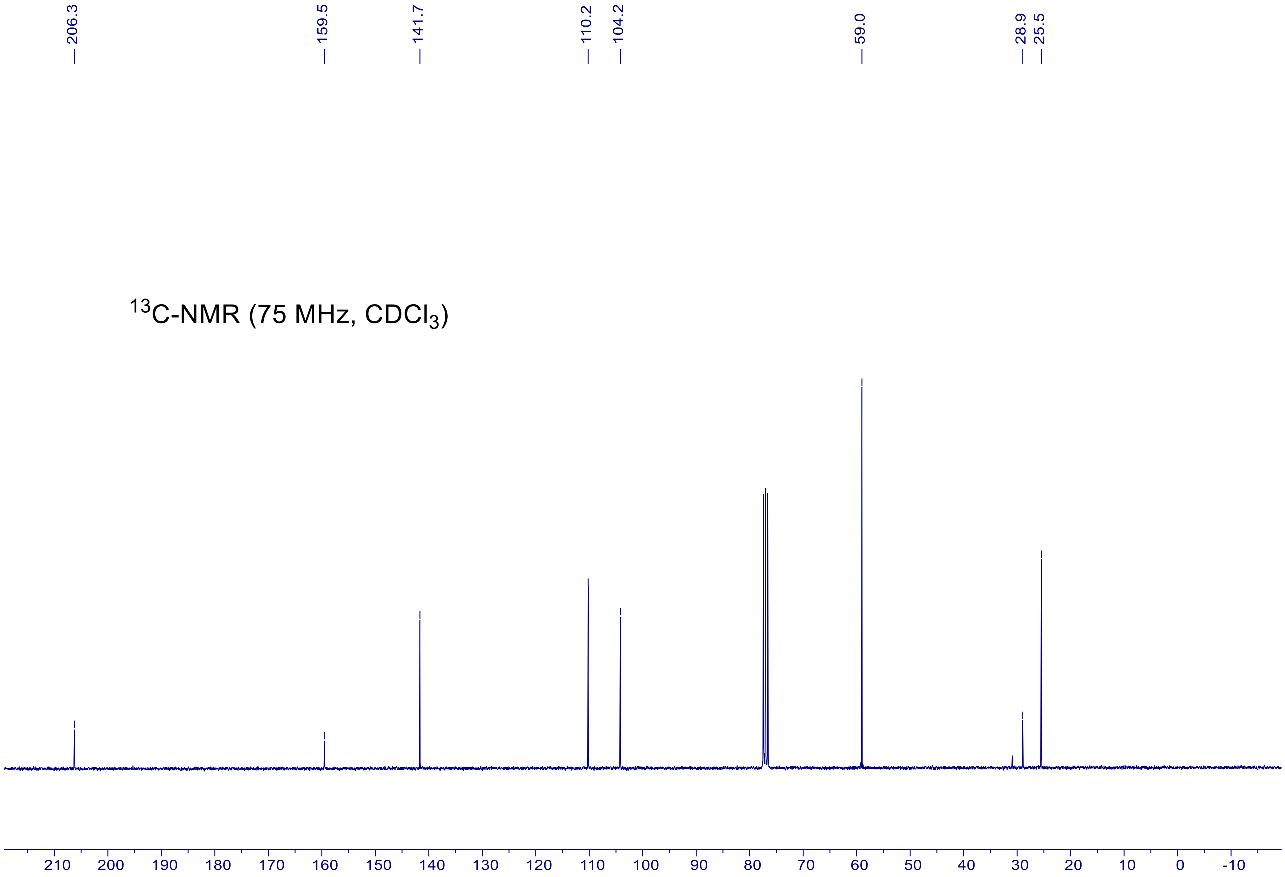

Figure SI-8. NMR spectra of compound **1q**.

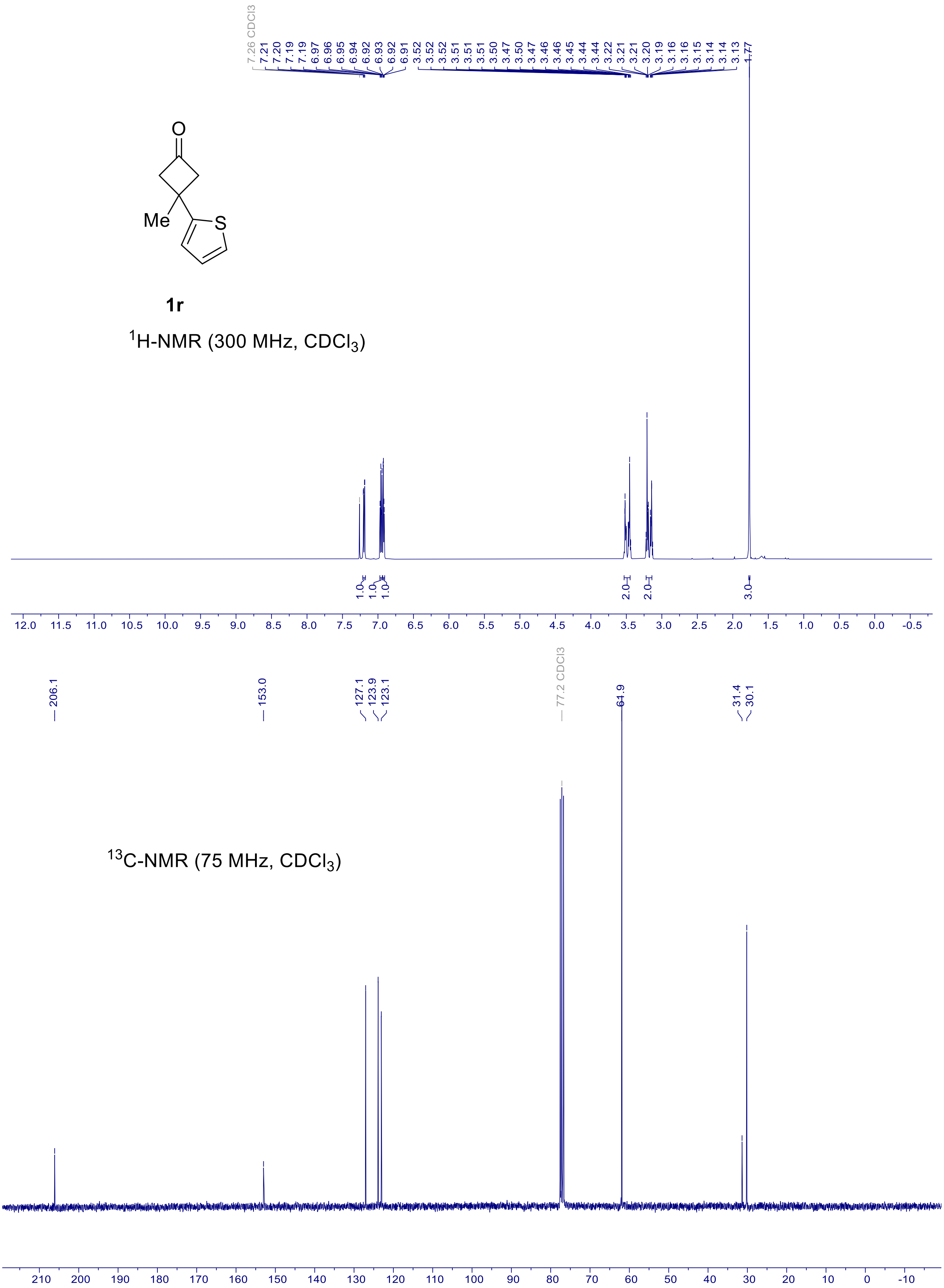

Figure SI-9. NMR spectra of compound **1r**.

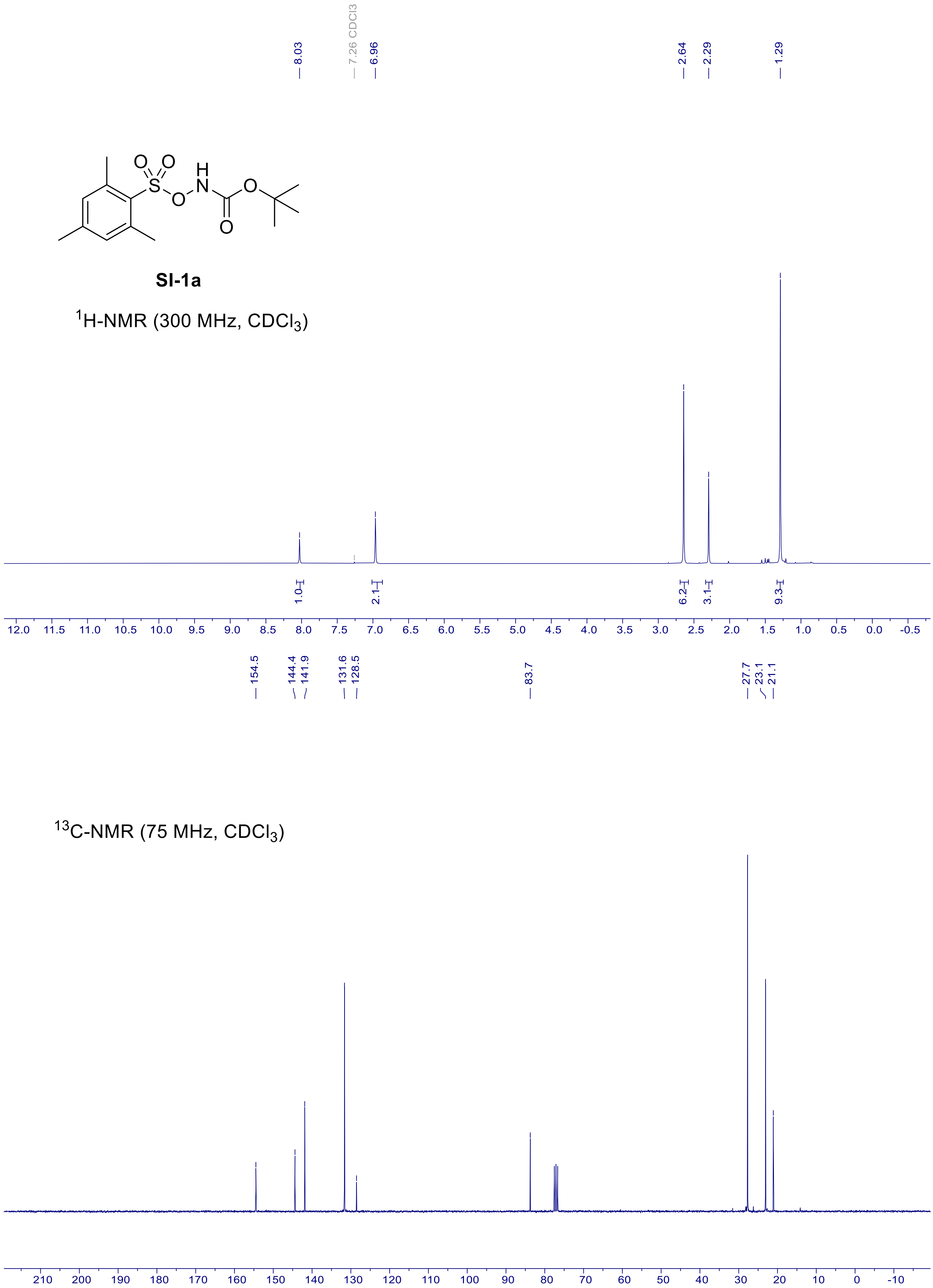

Figure SI-10. NMR spectra of compound **SI-1a**.

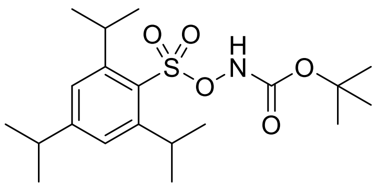

SI-1b

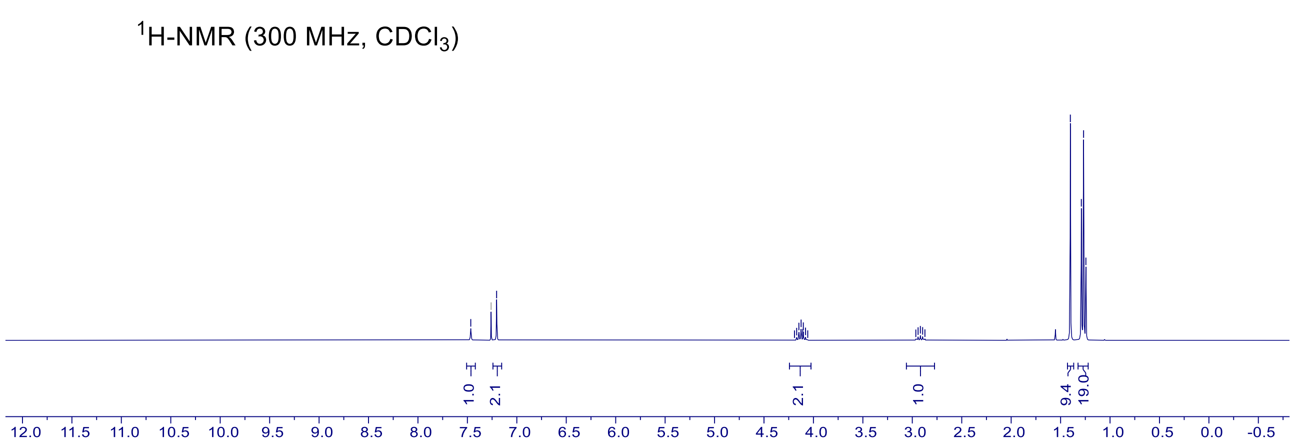

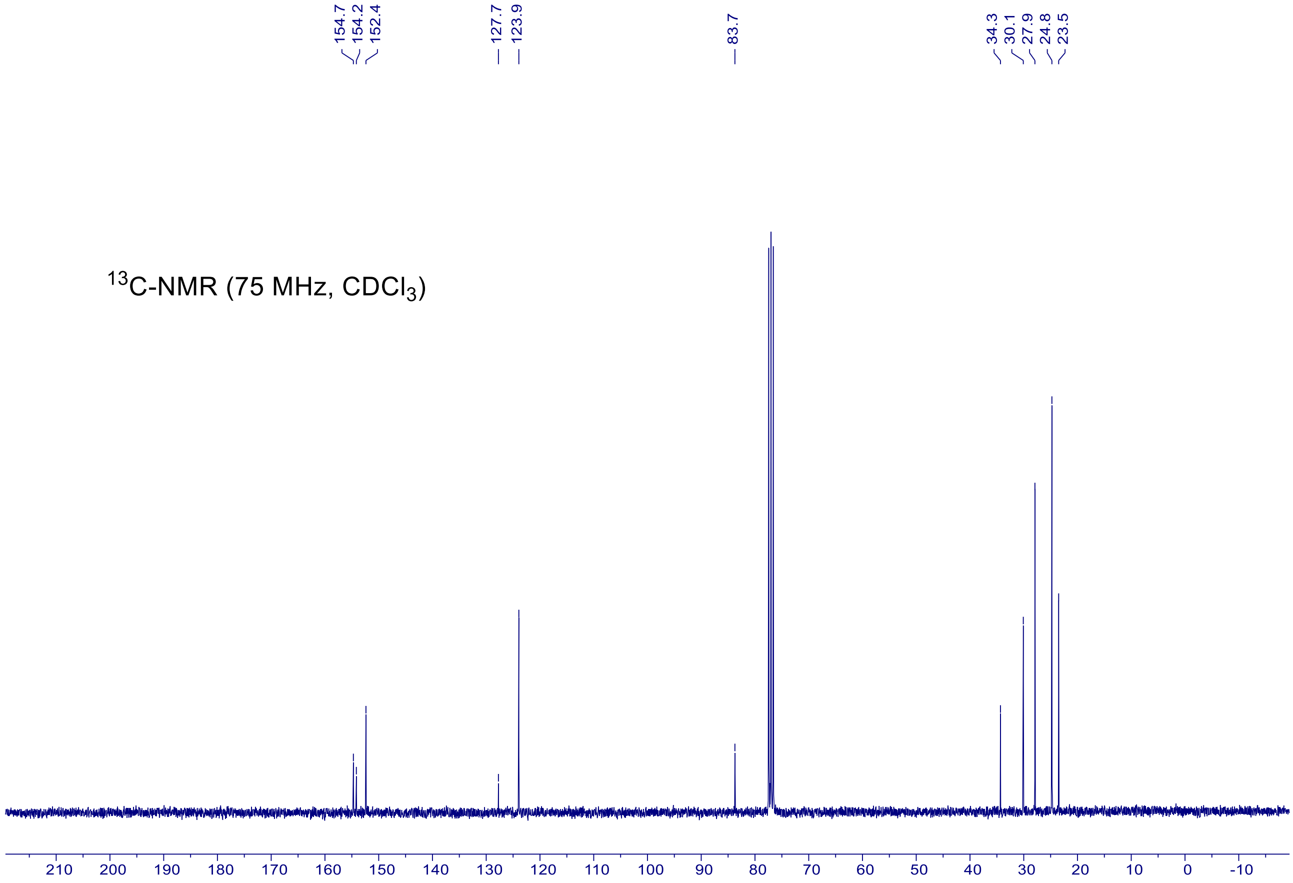

Figure SI-11. NMR spectra of compound **SI-1b**.

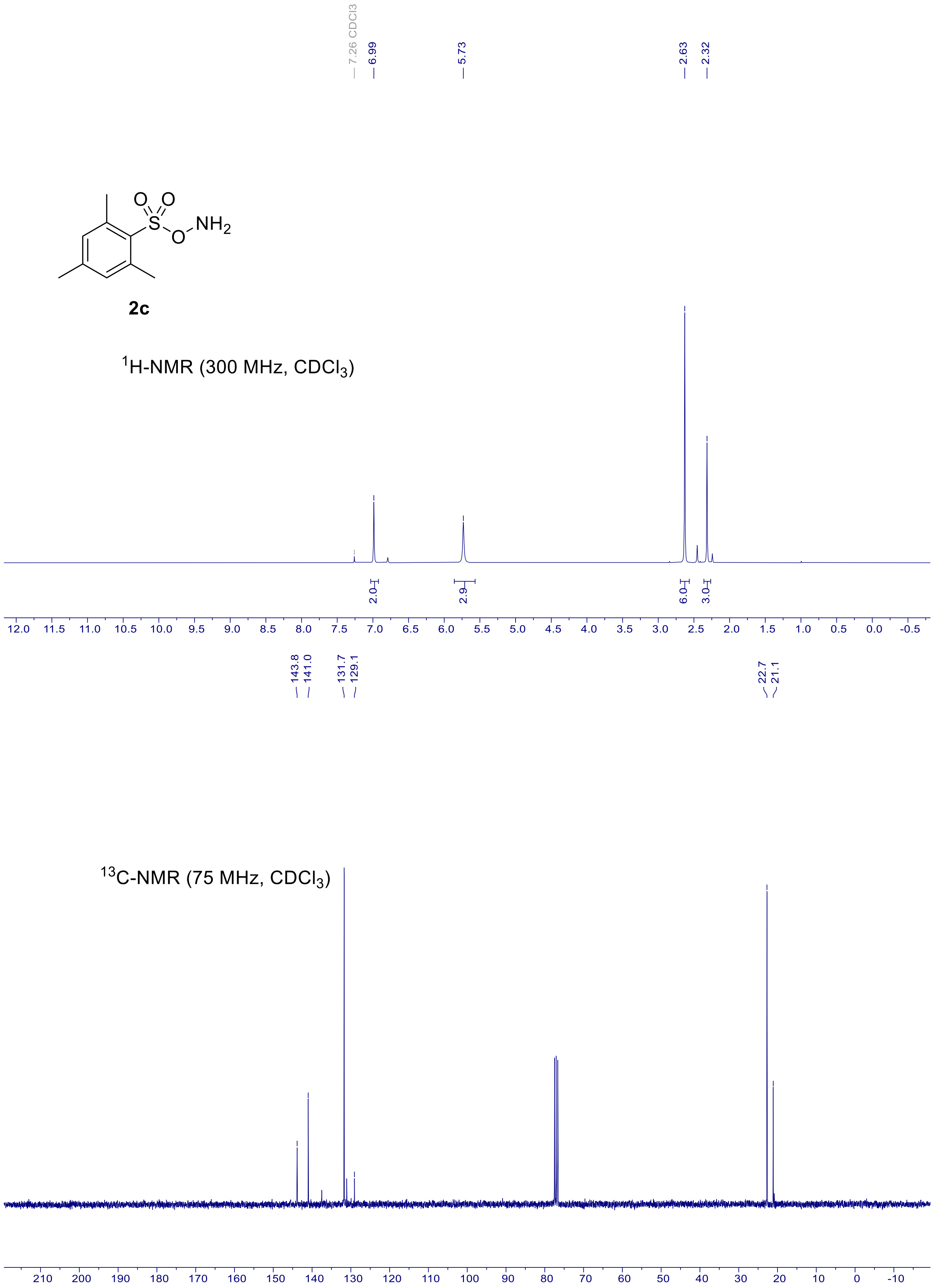

Figure SI-12. NMR spectra of compound **2c.**

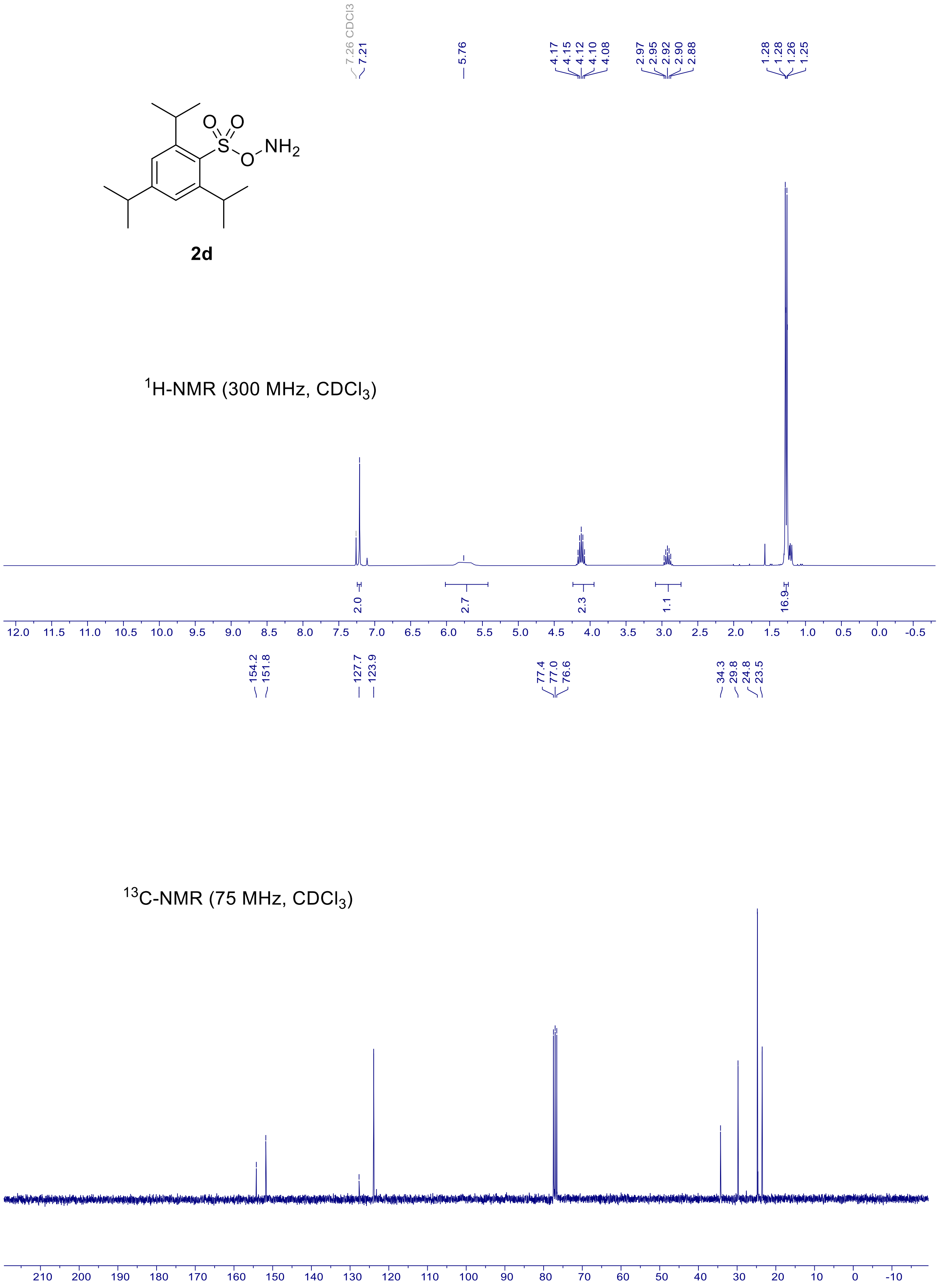

Figure SI-13. NMR spectra of compound **2d**.

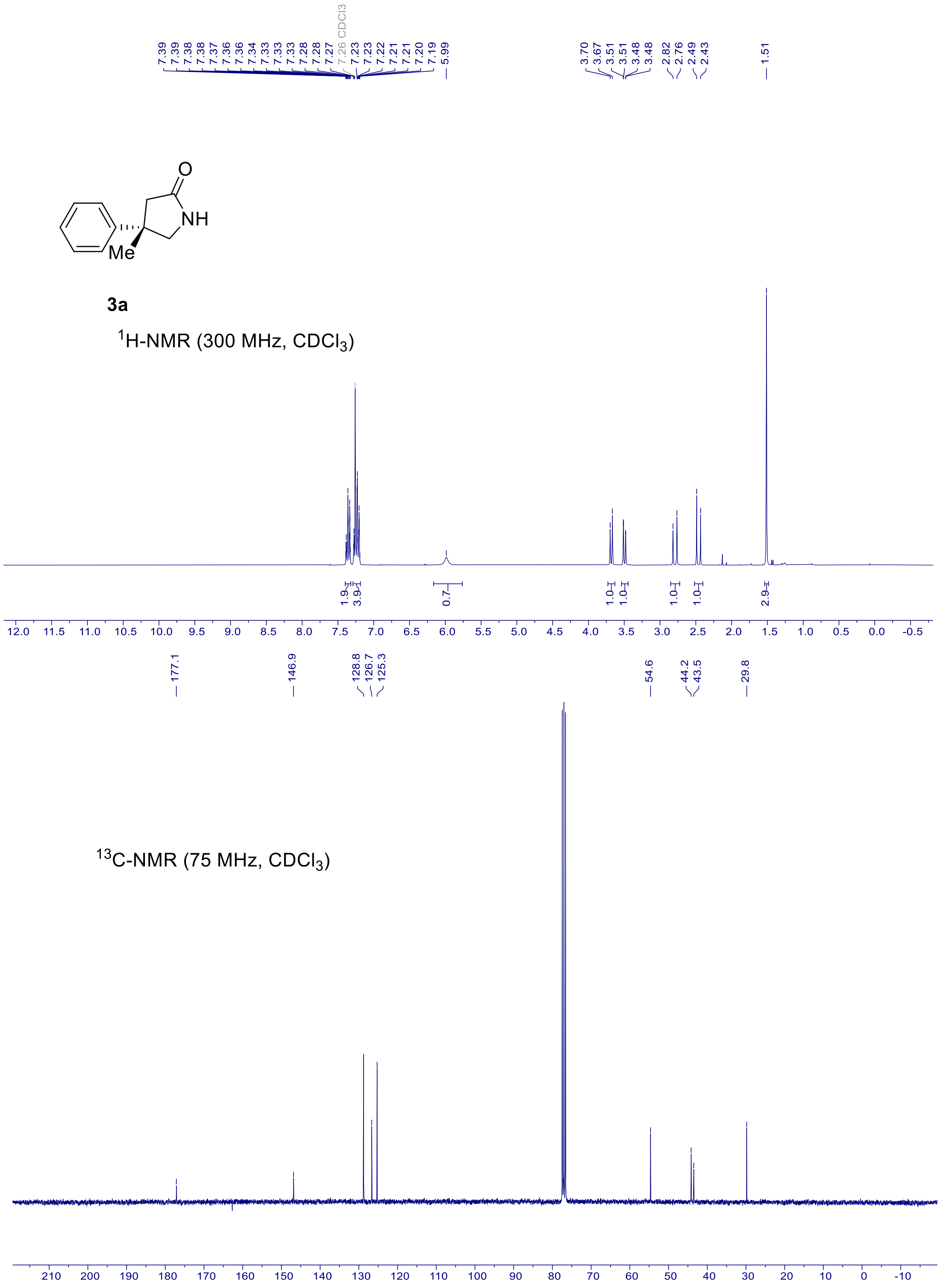

Figure SI-14. NMR spectra of compound **3a**.

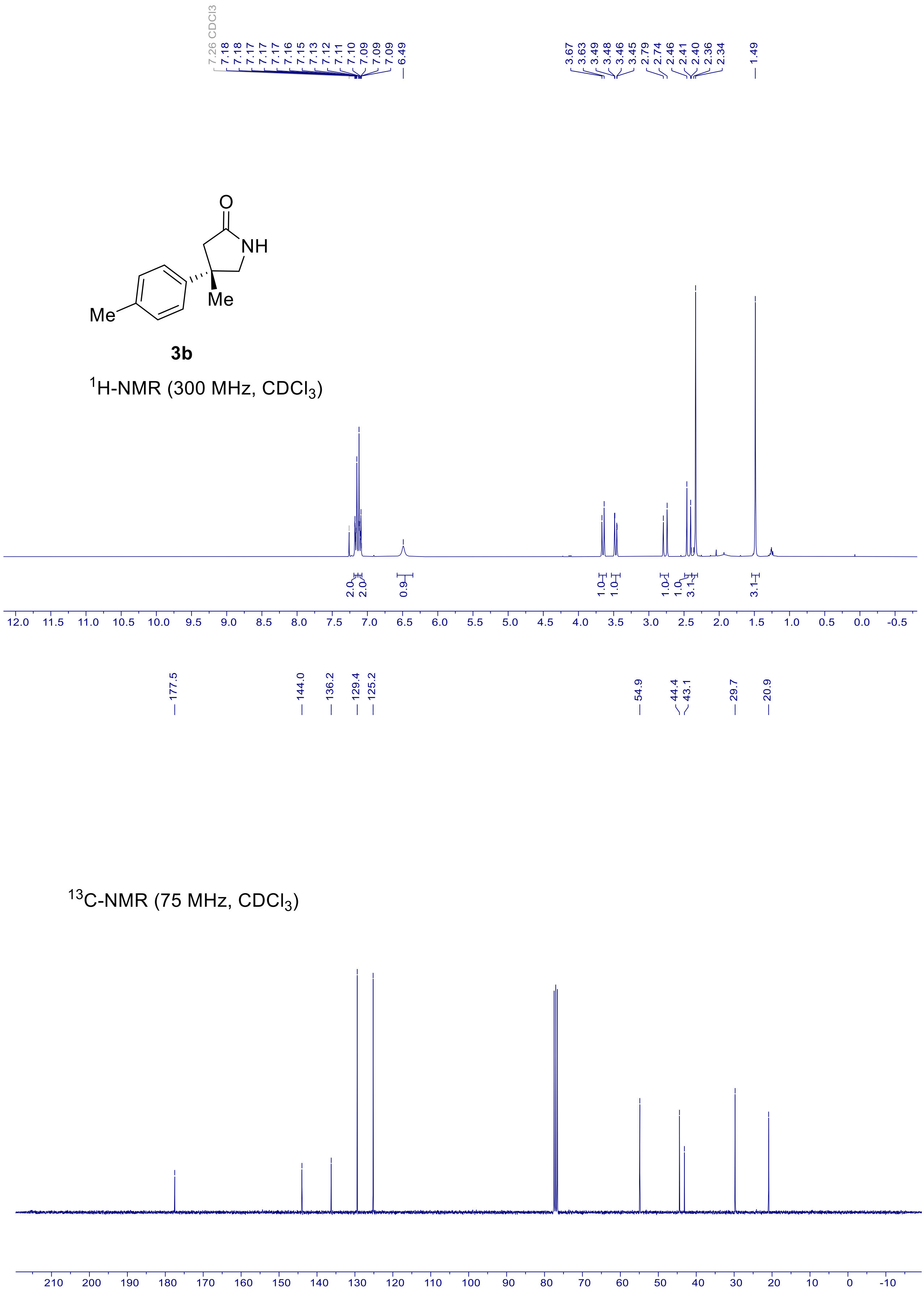

Figure SI-15. NMR spectra of compound **3b**.

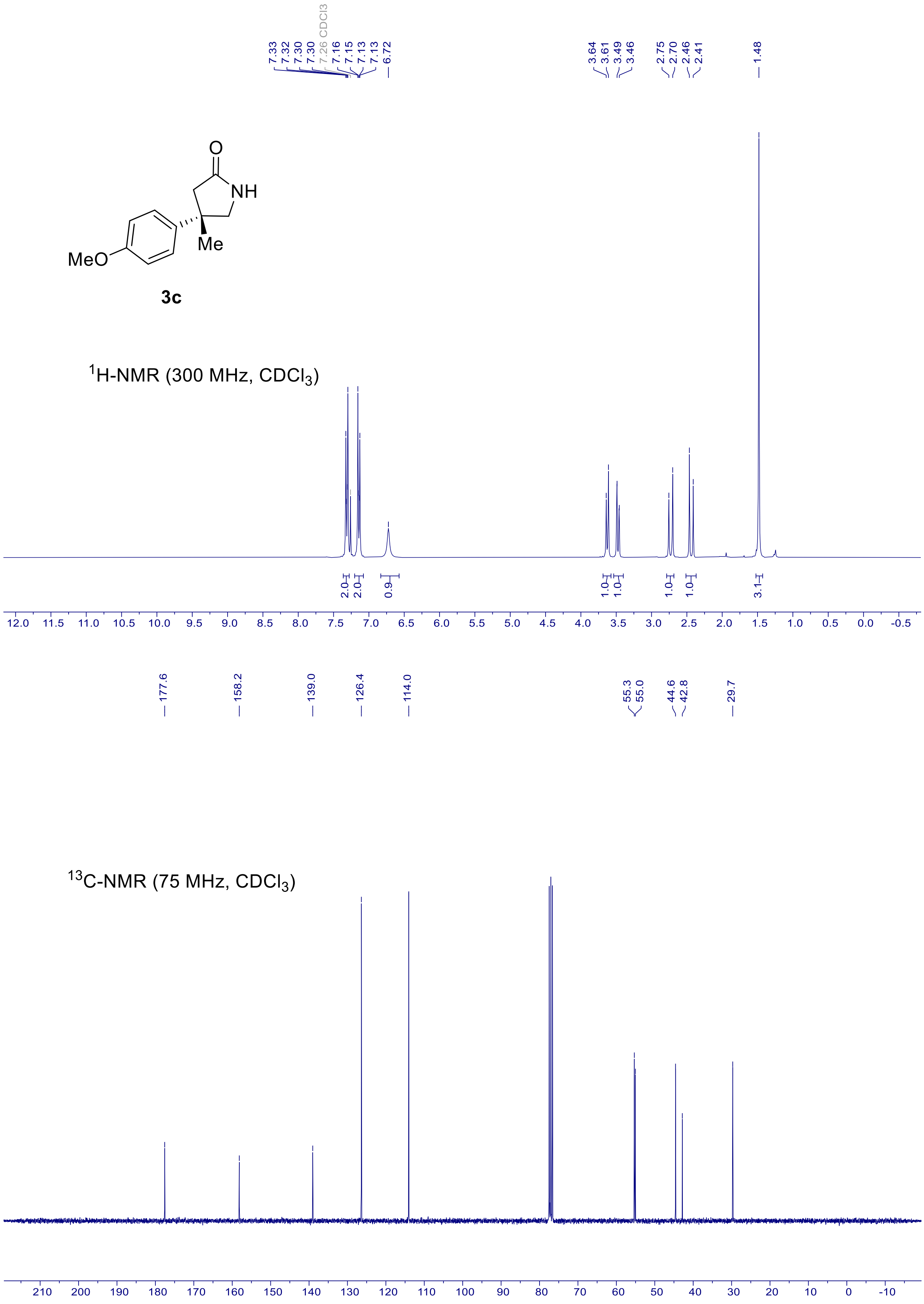

Figure SI-16. NMR spectra of compound **3c**.

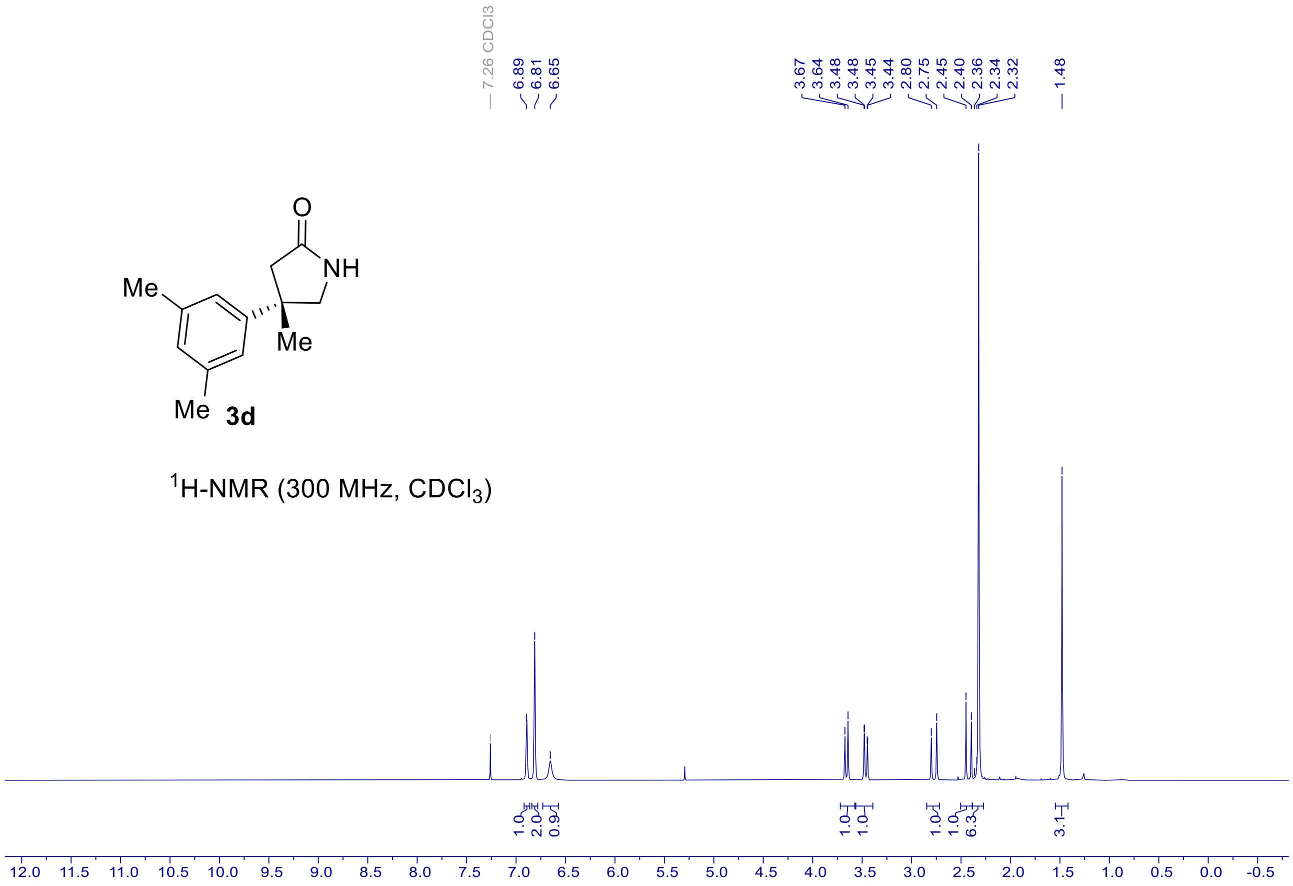

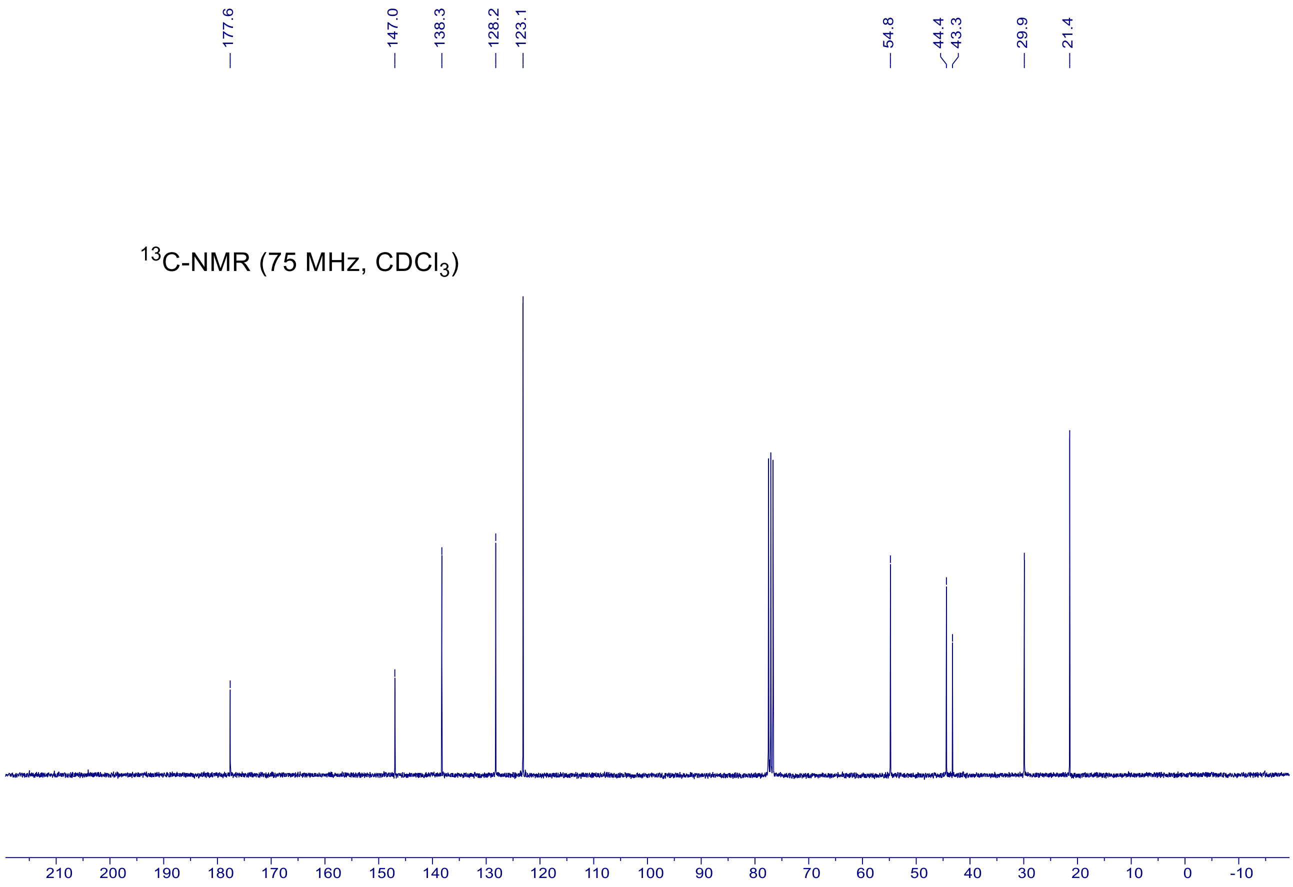

Figure SI-17. NMR spectra of compound **3d**.

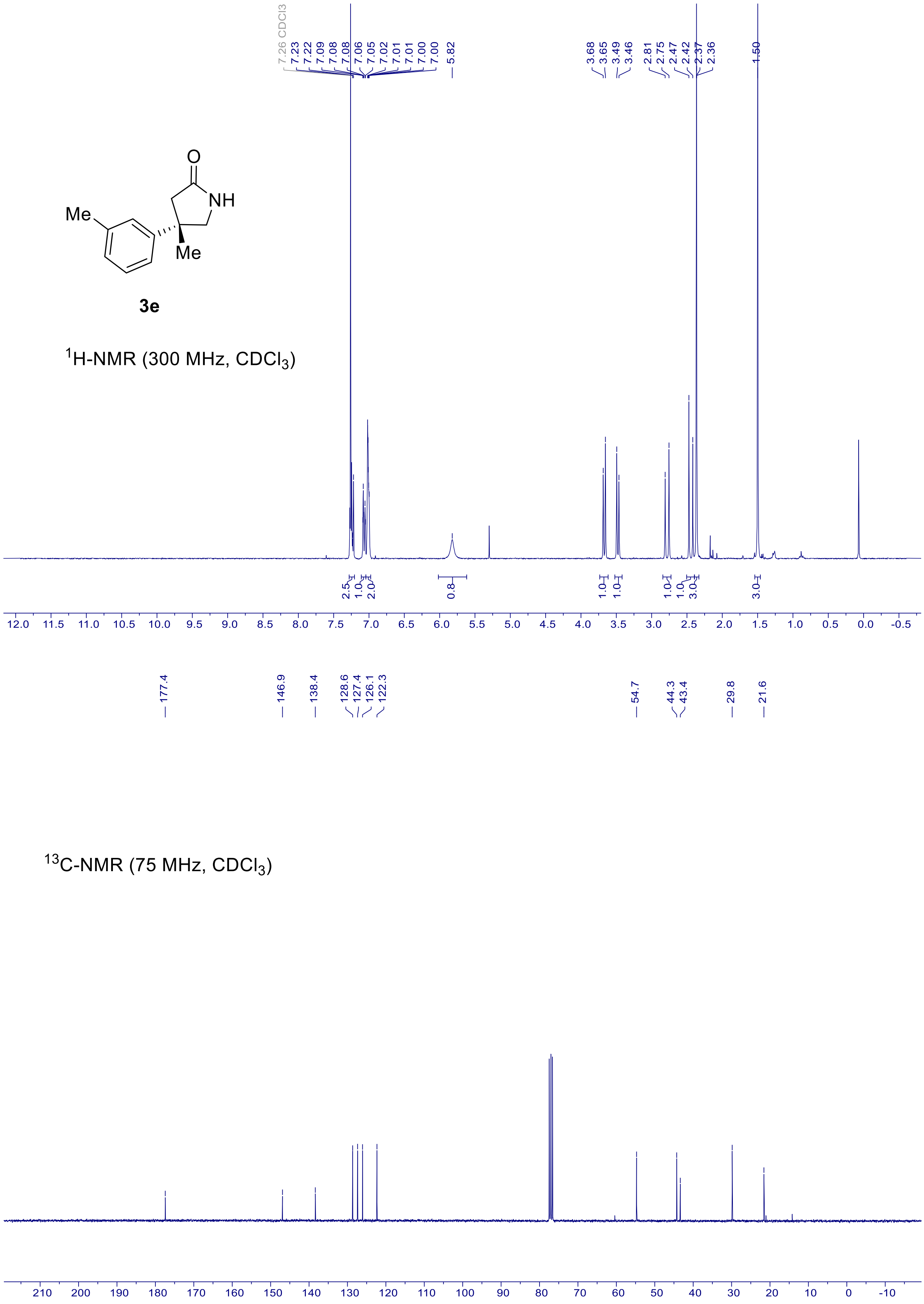

Figure SI-18. NMR spectra of compound **3e**.

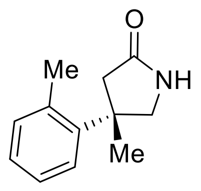

3f

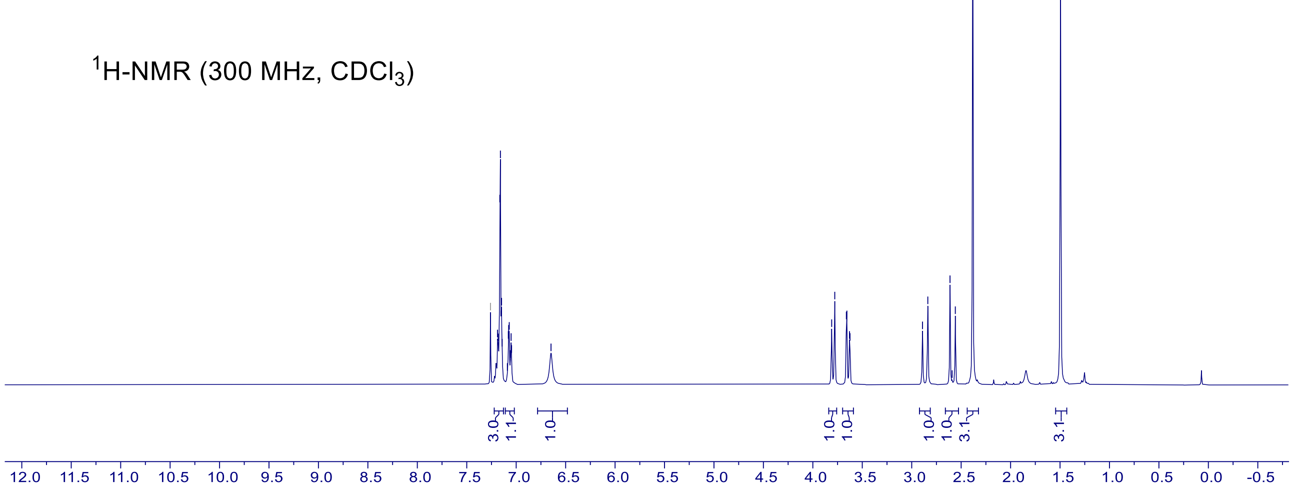

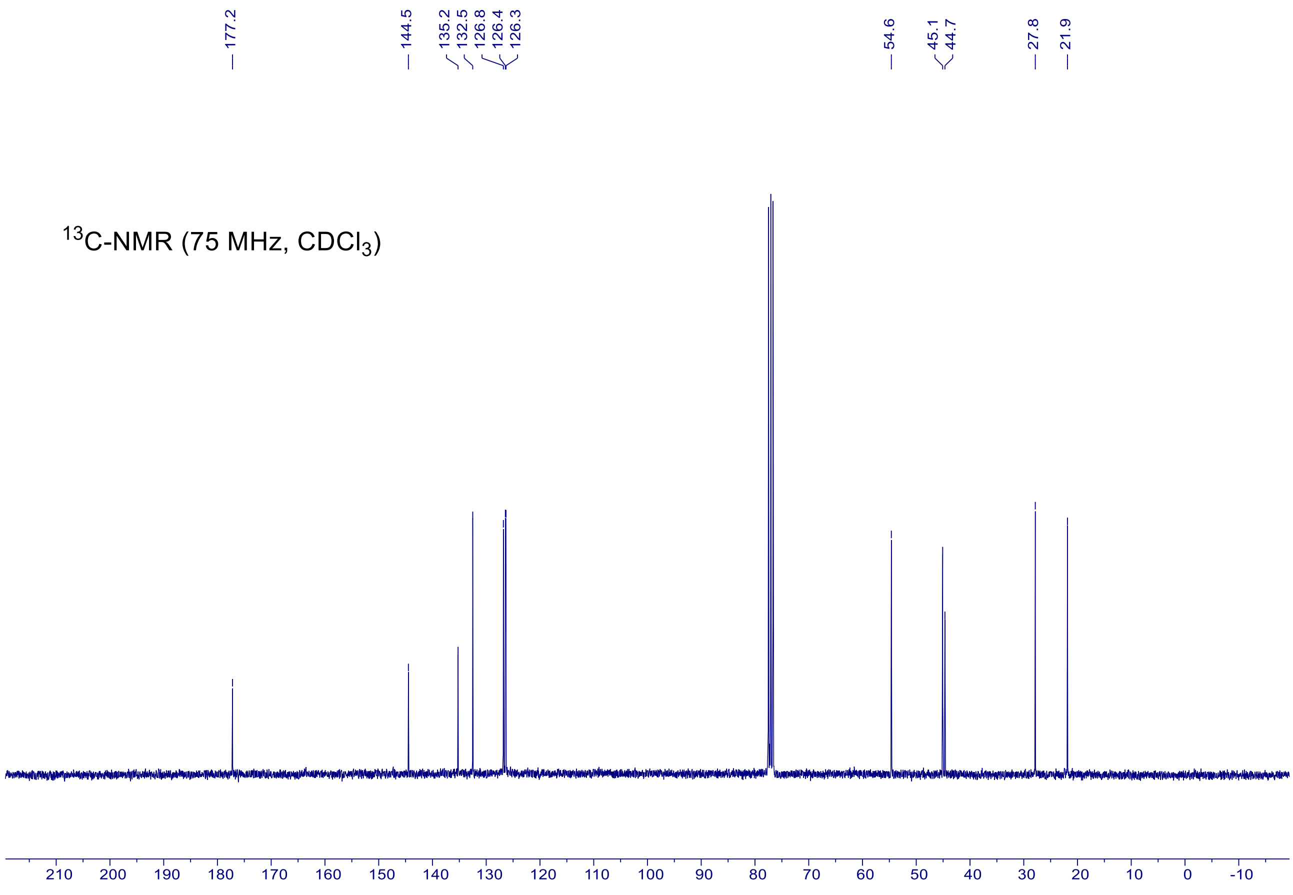

Figure SI-19. NMR spectra of compound **3f**.

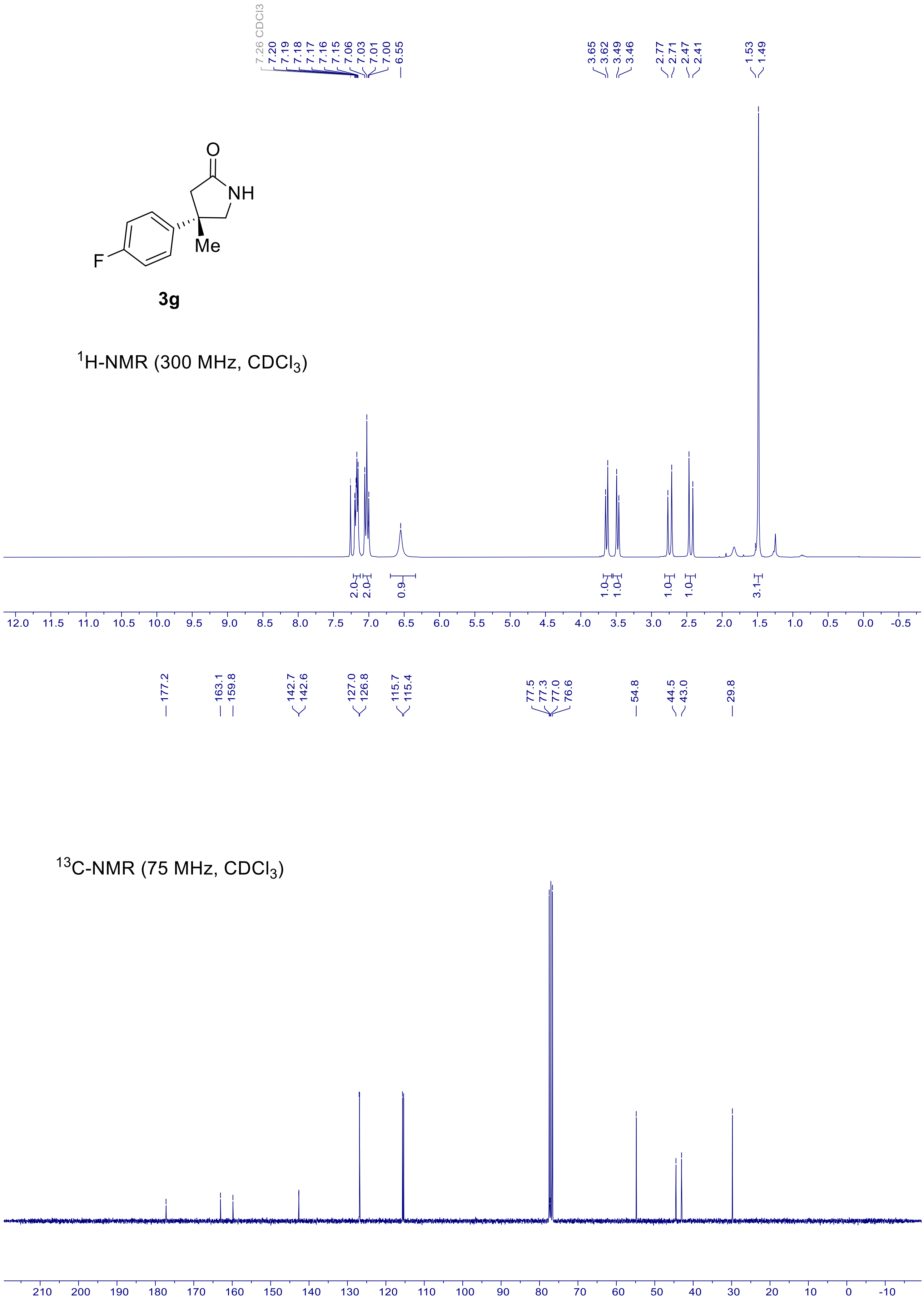

Figure SI-20. NMR spectra of compound **3g**.

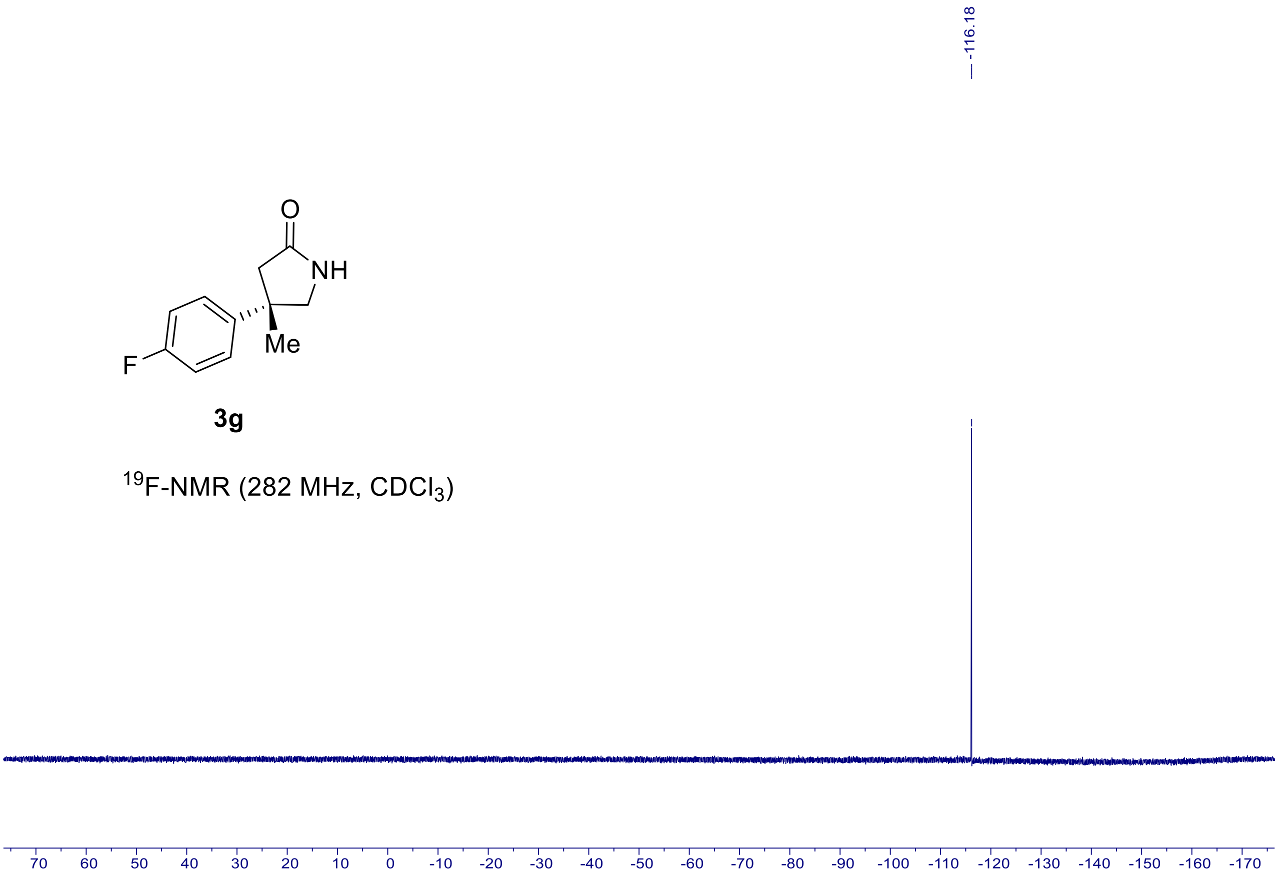

Figure SI-20. NMR spectra of compound **3g** *(continued)*.

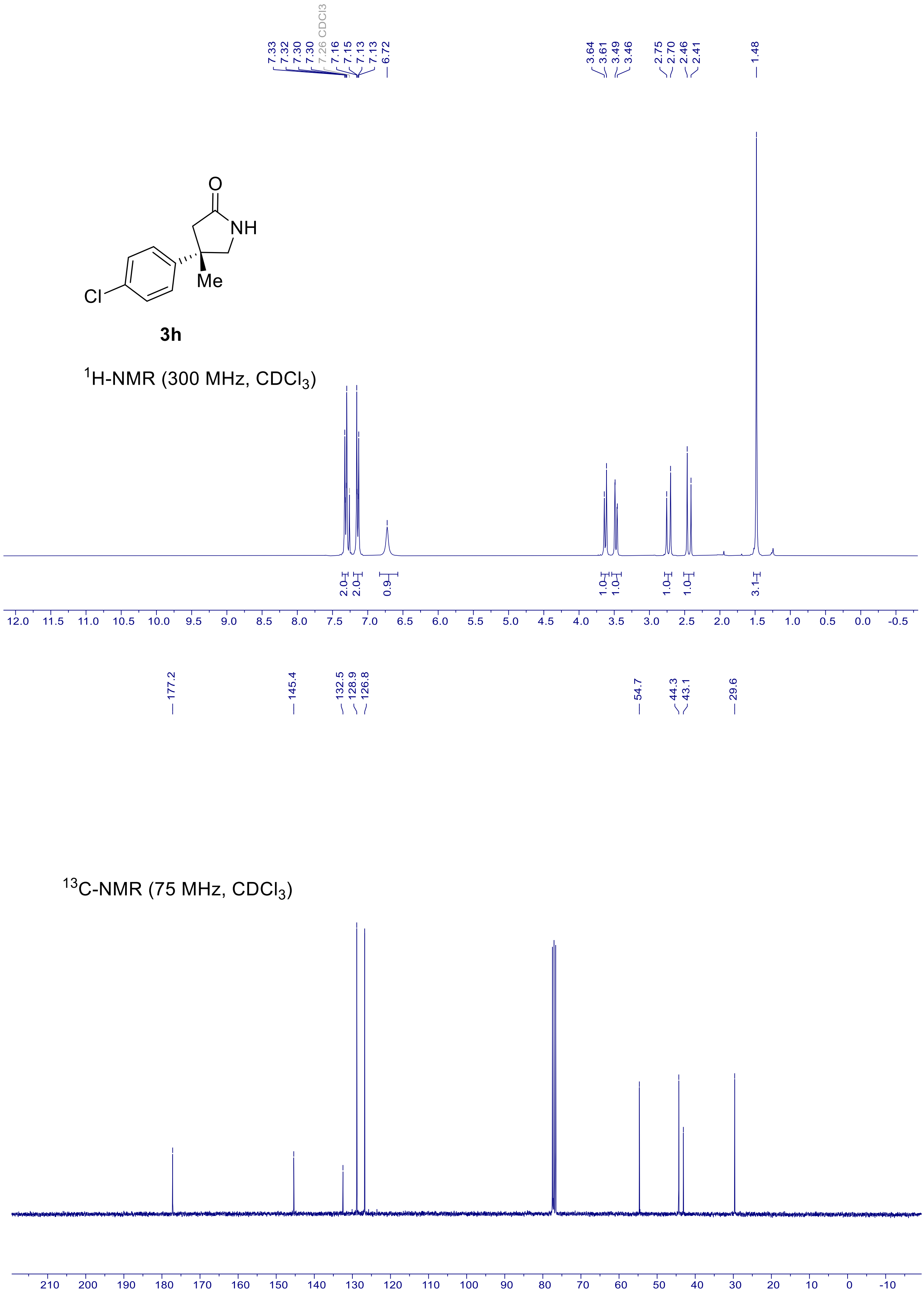

Figure SI-21. NMR spectra of compound **3h**.

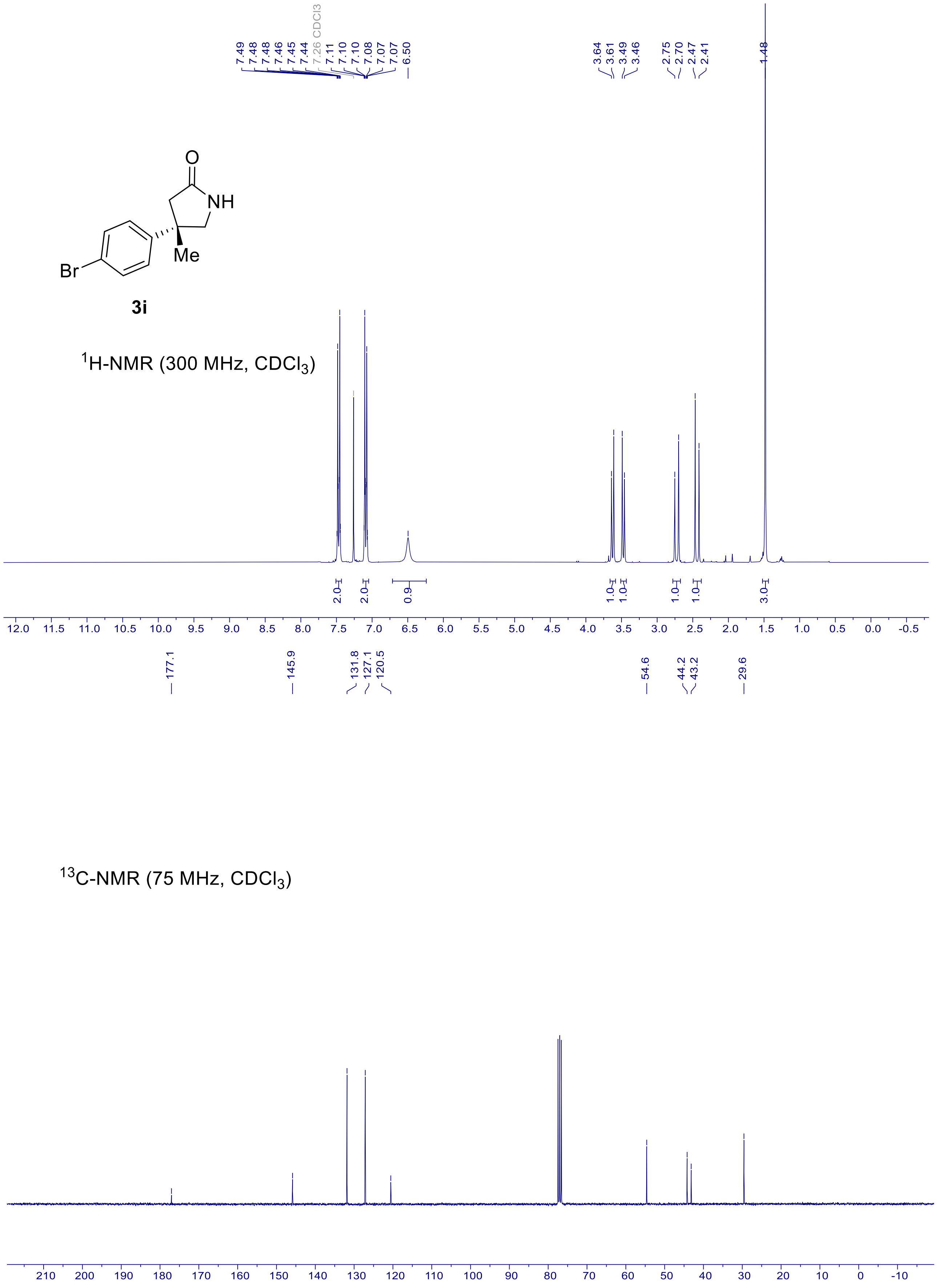

Figure SI-22. NMR spectra of compound **3i.**

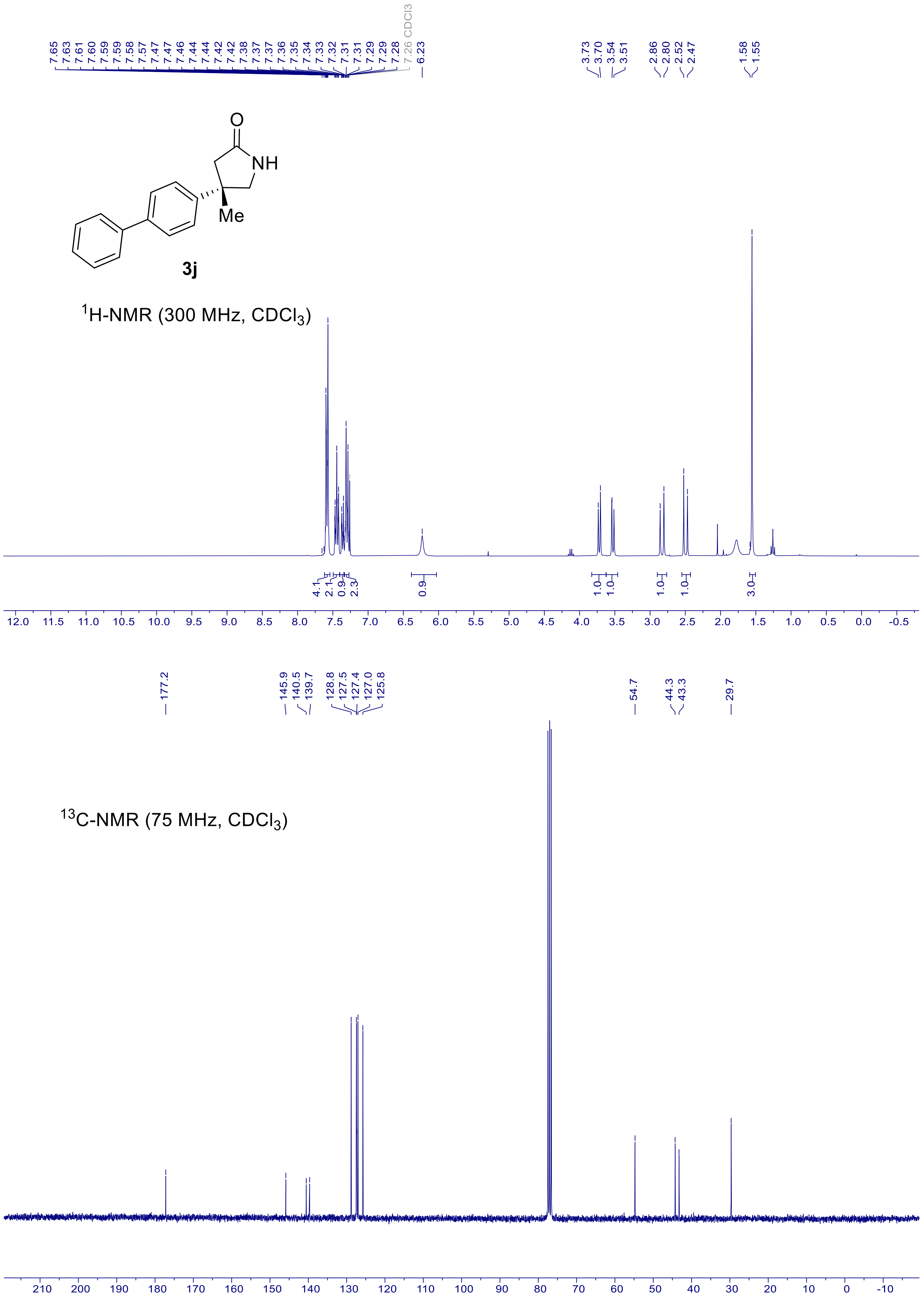

Figure SI-23. NMR spectra of compound **3j**.

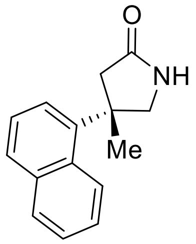

3k

^{1}H-NMR (300 MHz, $CDCl_3$)

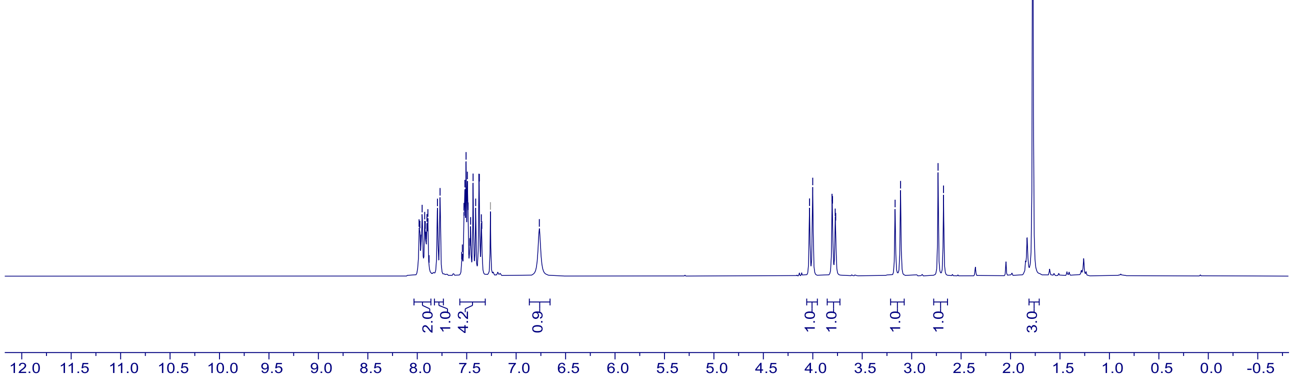

^{13}C-NMR (75 MHz, $CDCl_3$)

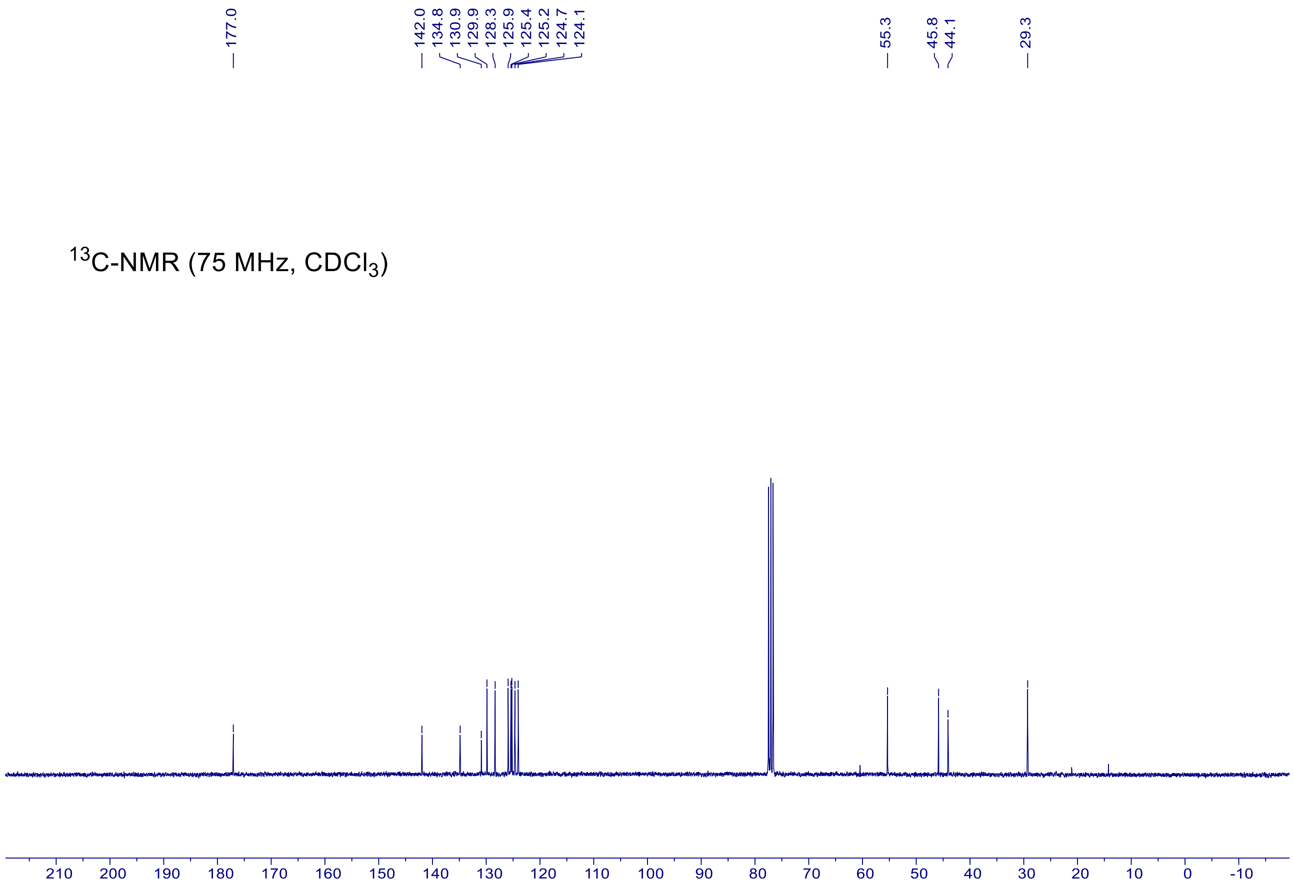

Figure SI-24. NMR spectra of compound **3k**.

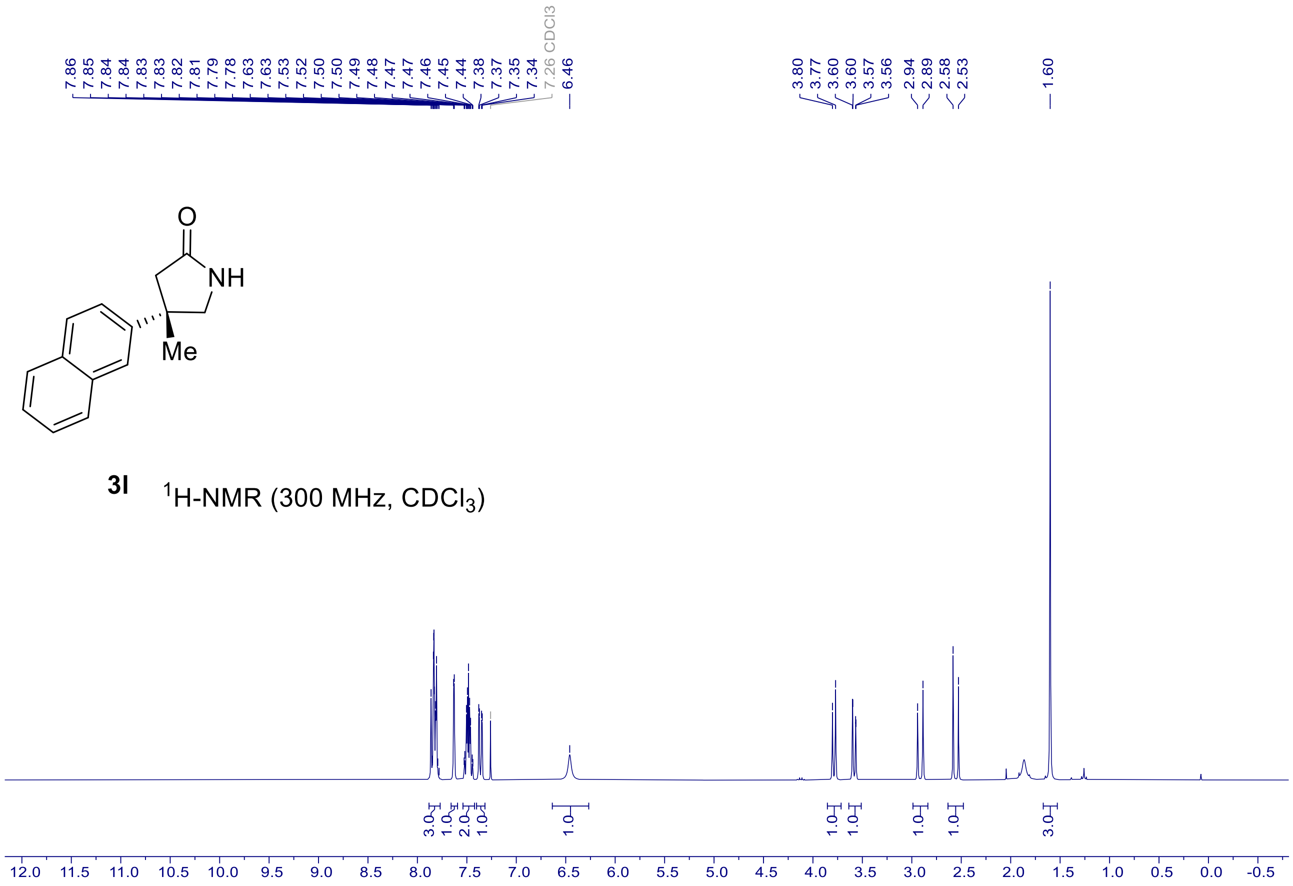

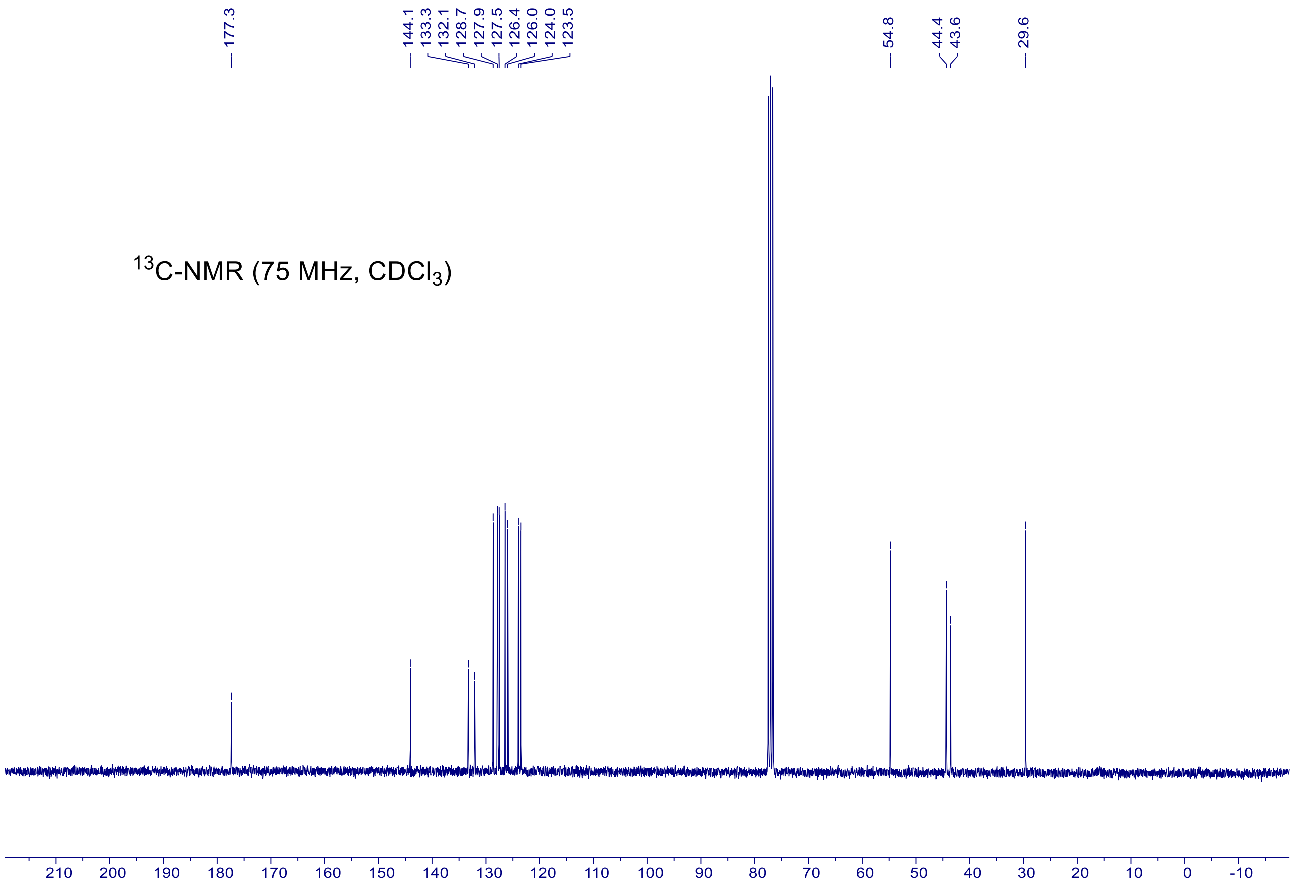

Figure SI-25. NMR spectra of compound **3l.**

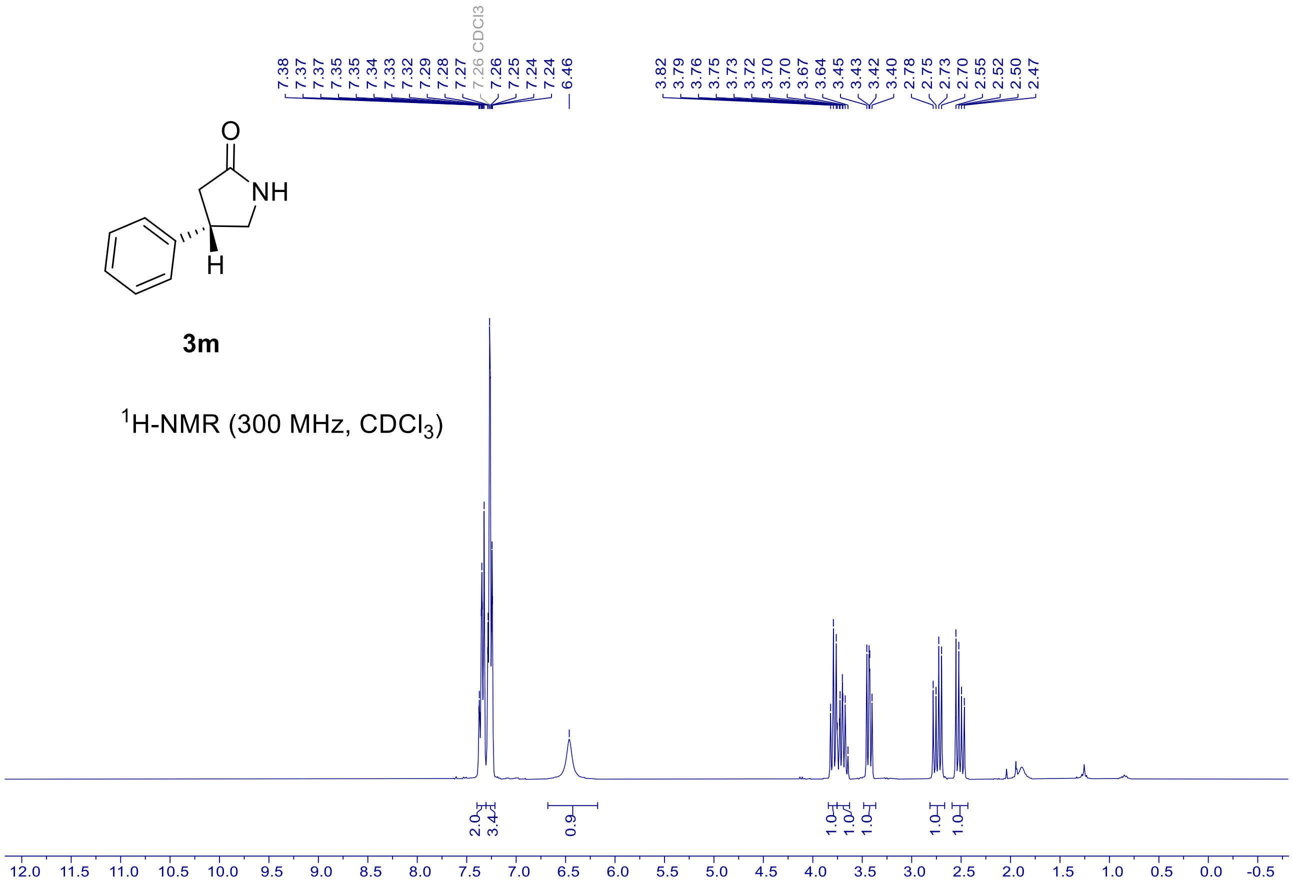

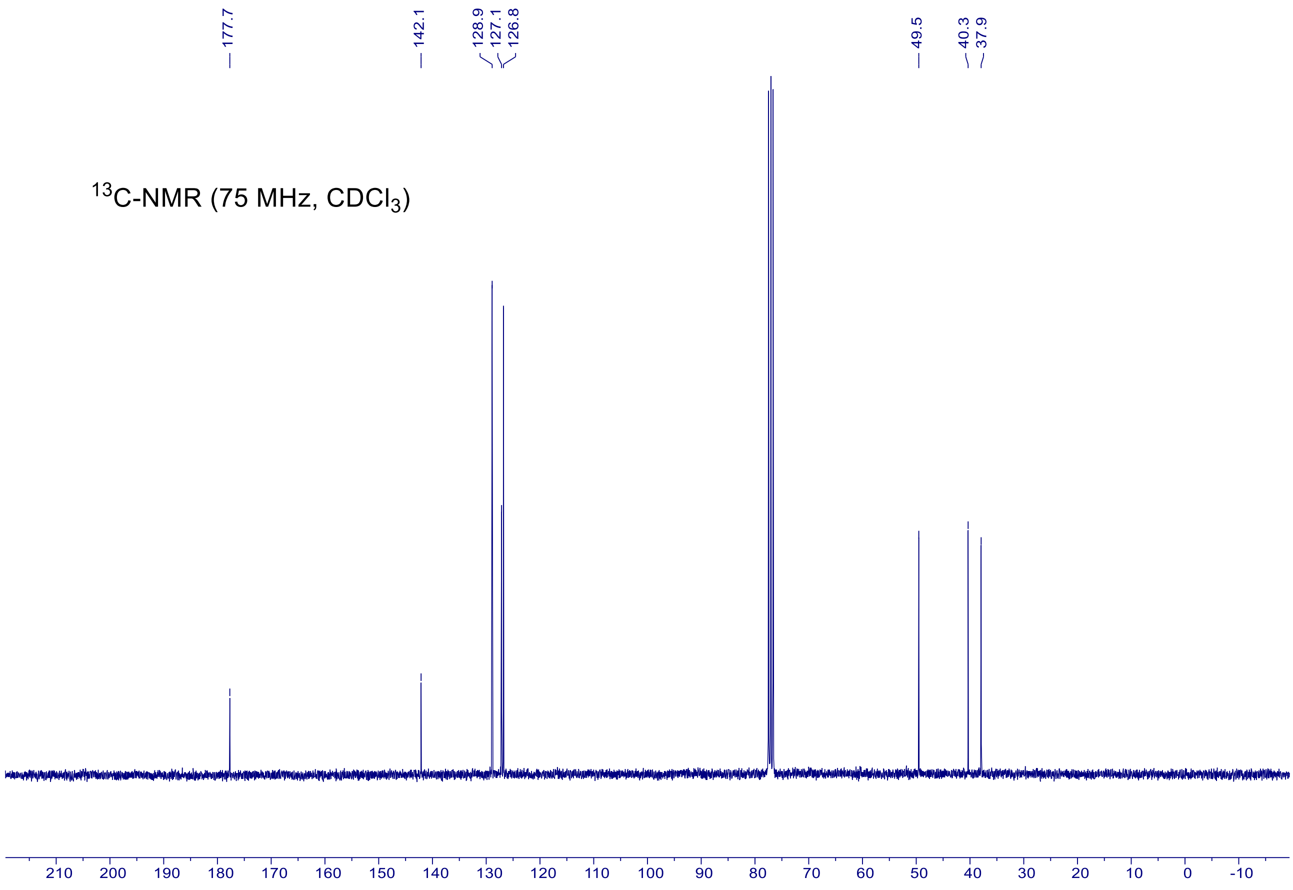

Figure SI-26. NMR spectra of compound **3m.**

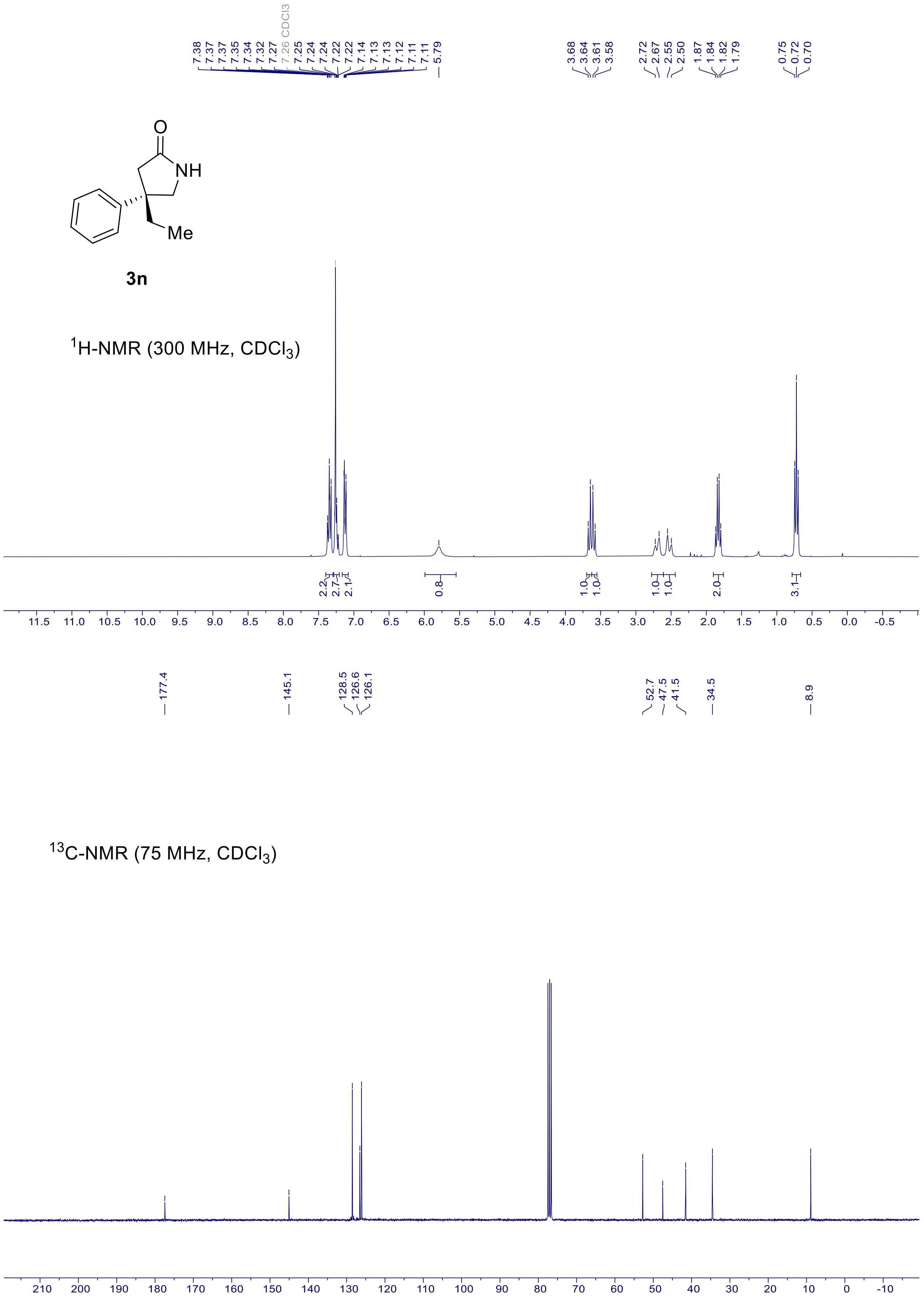

Figure SI-27. NMR spectra of compound **3n**.

3o

^{1}H-NMR (300 MHz, $CDCl_3$)

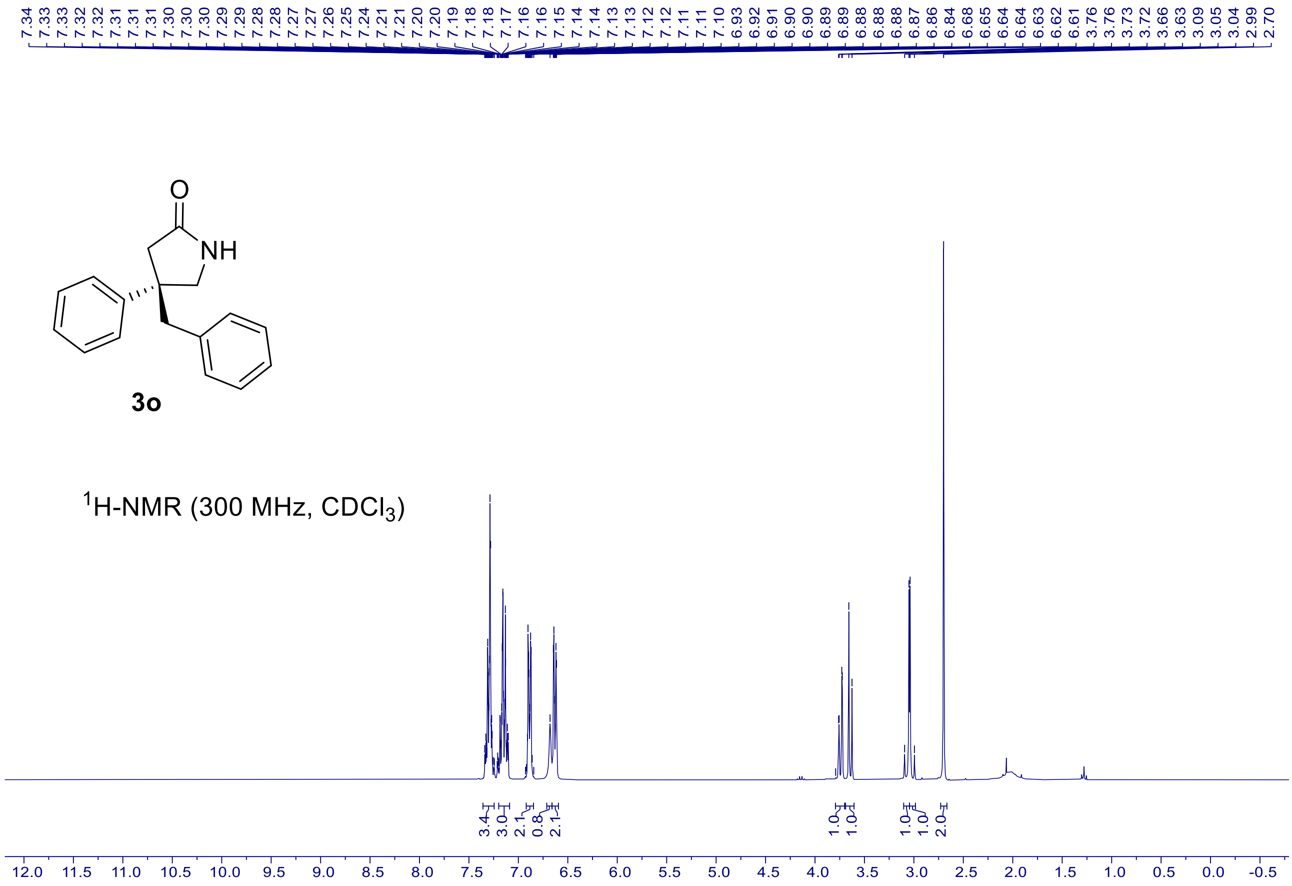

^{13}C-NMR (75 MHz, $CDCl_3$)

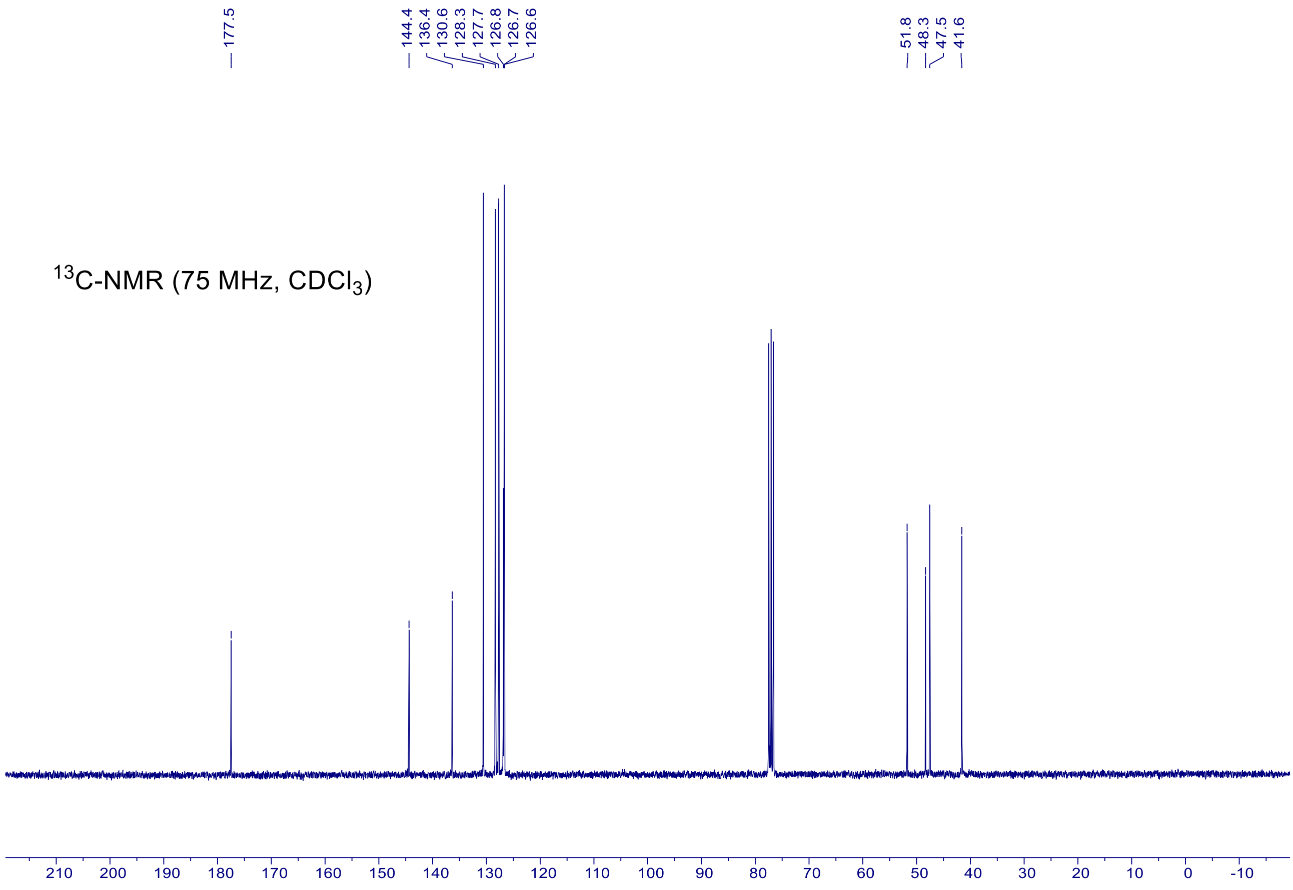

Figure SI-28. NMR spectra of compound **3o**.

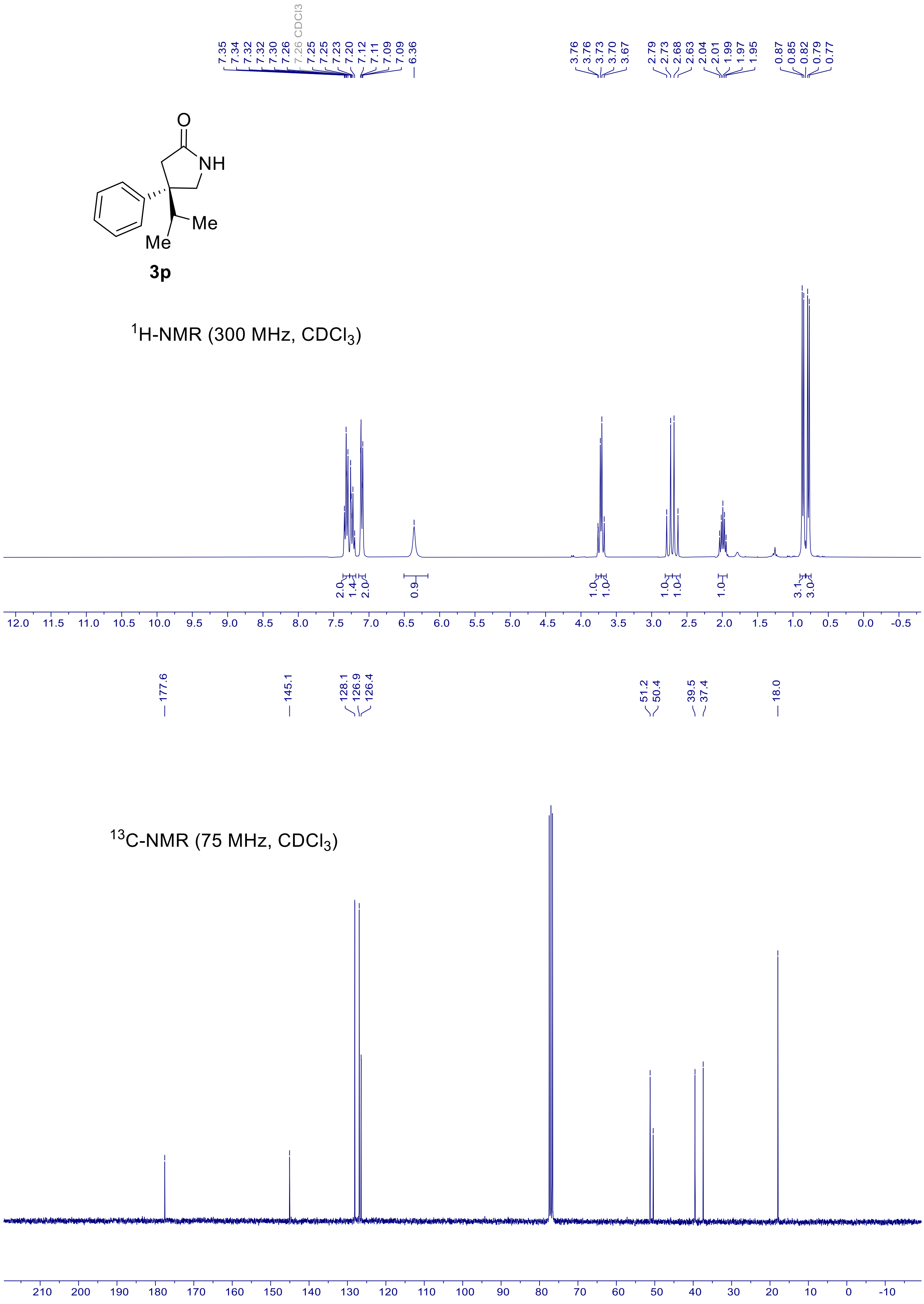

Figure SI-29. NMR spectra of compound **3p**.

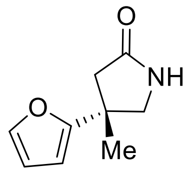

3q

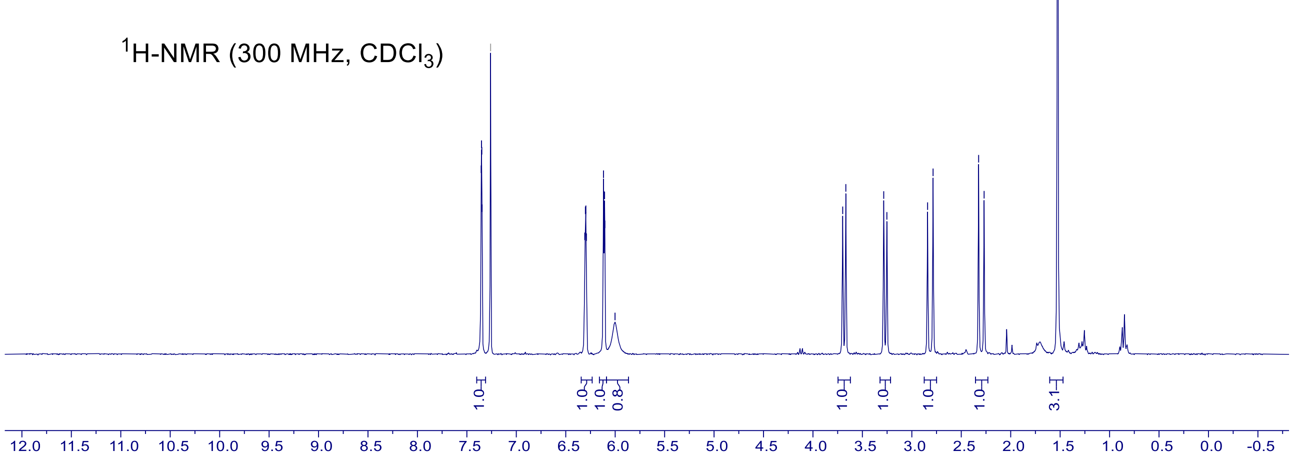

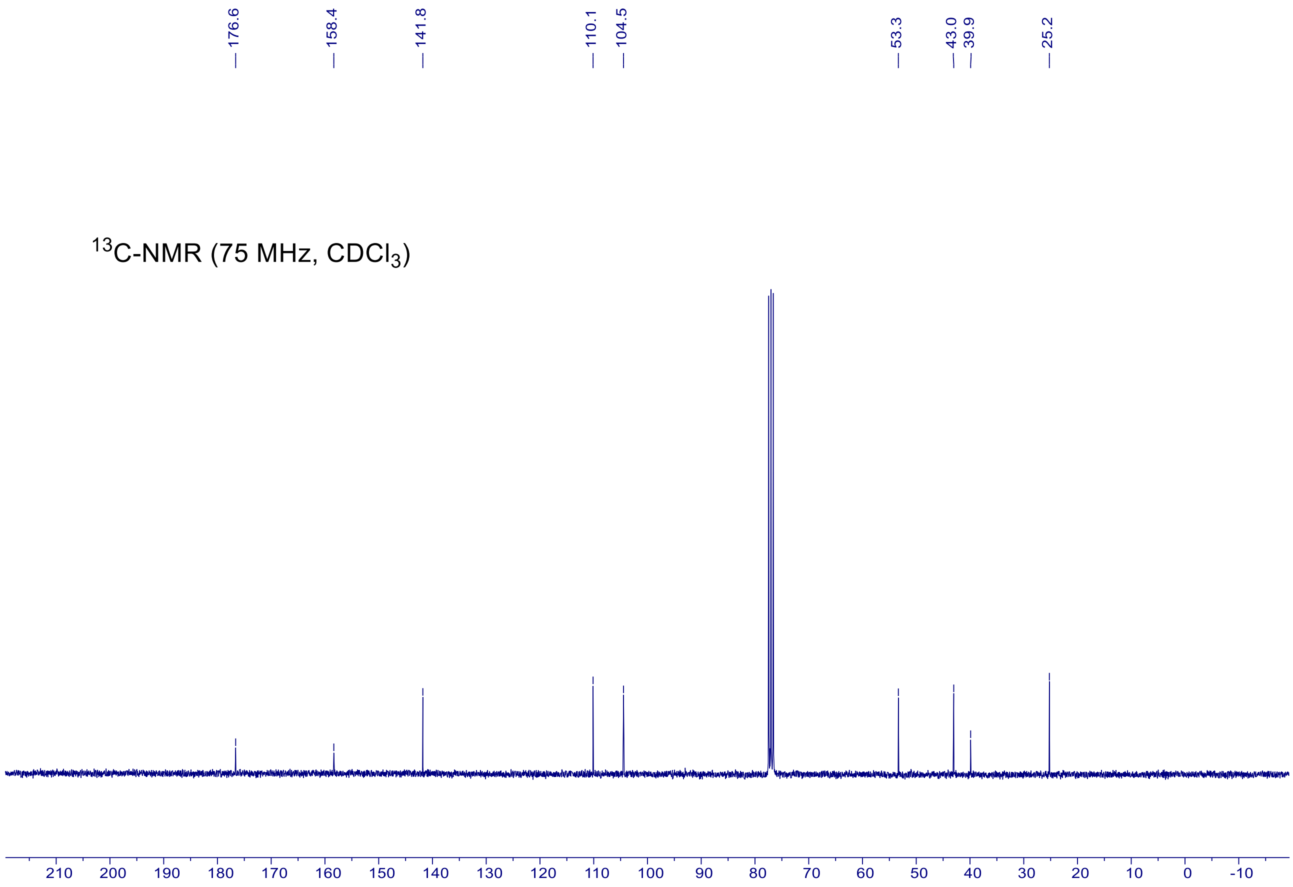

Figure SI-30. NMR spectra of compound **3q.**

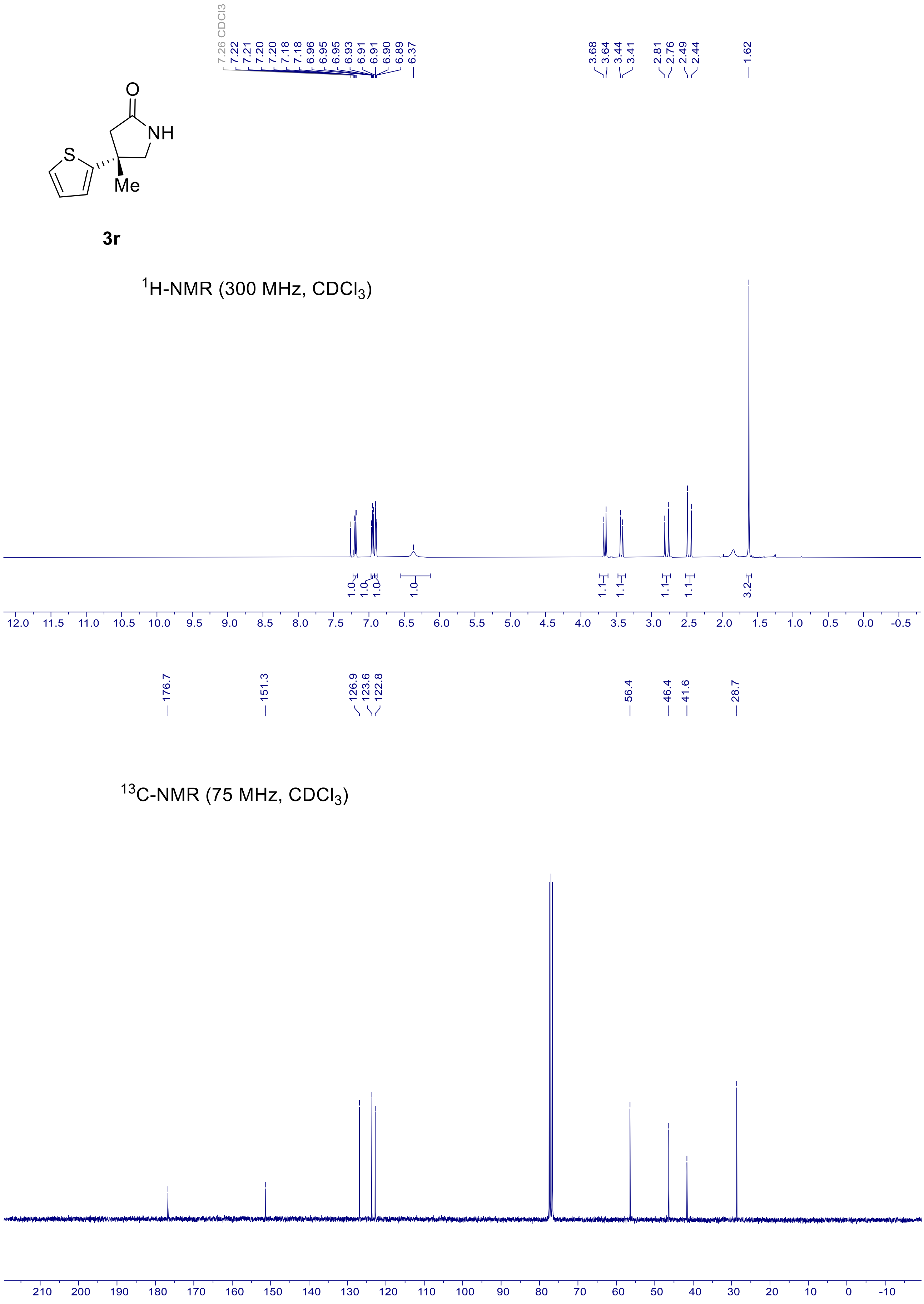

Figure SI-31. NMR spectra of compound **3r.**

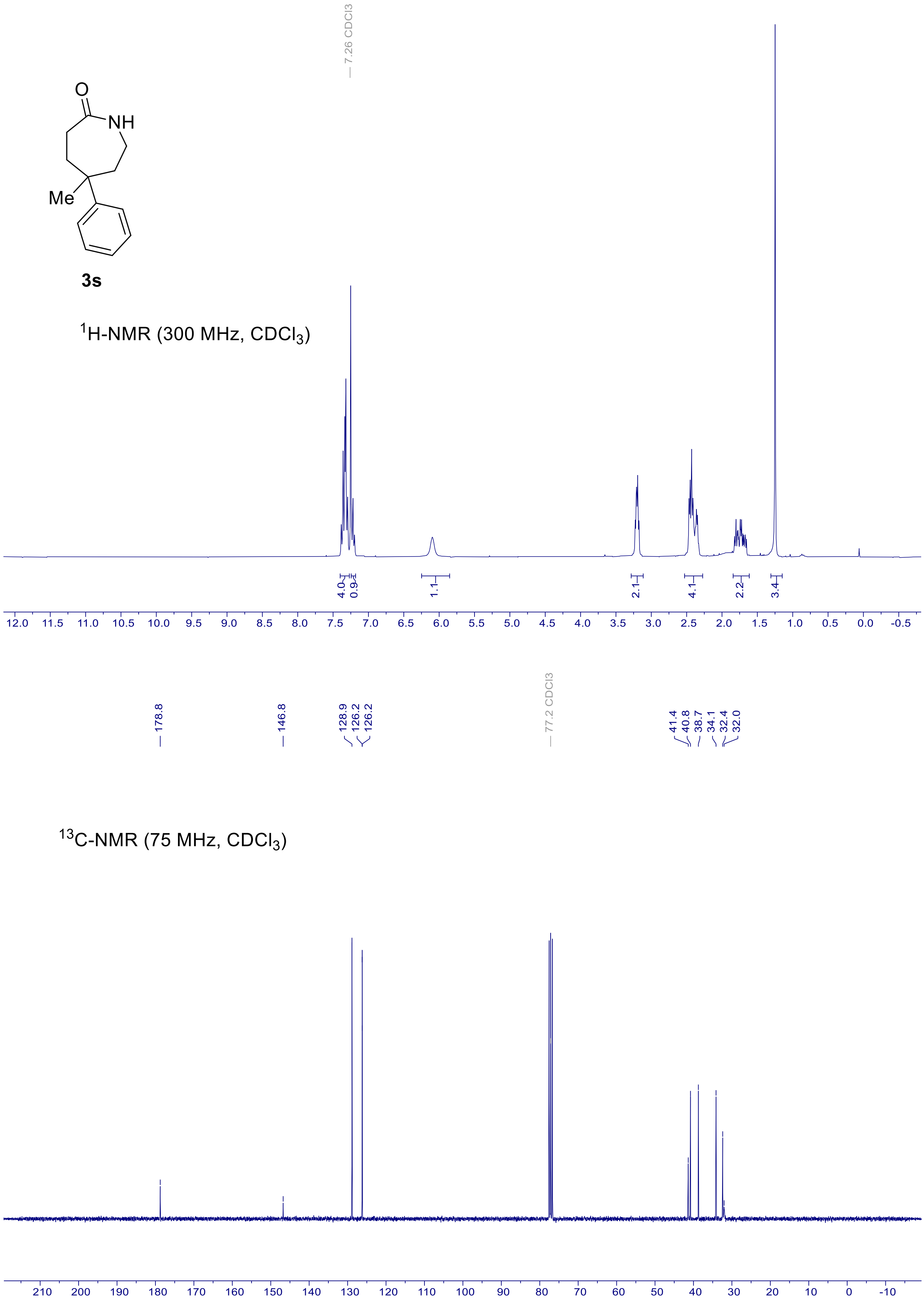

Figure SI-32. NMR spectra of compound **3s**.

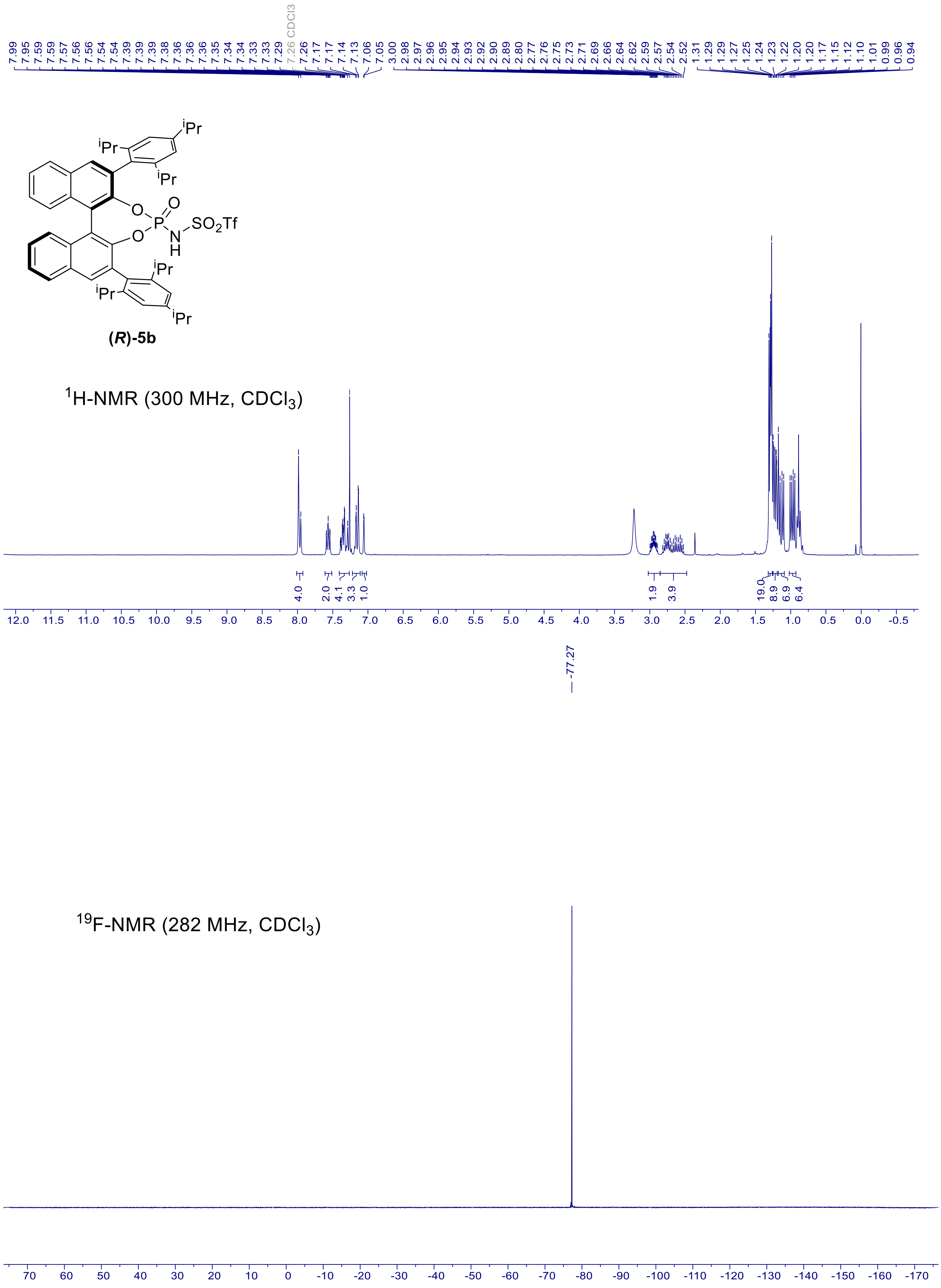

Figure SI-33. NMR spectra of compound **5b**.

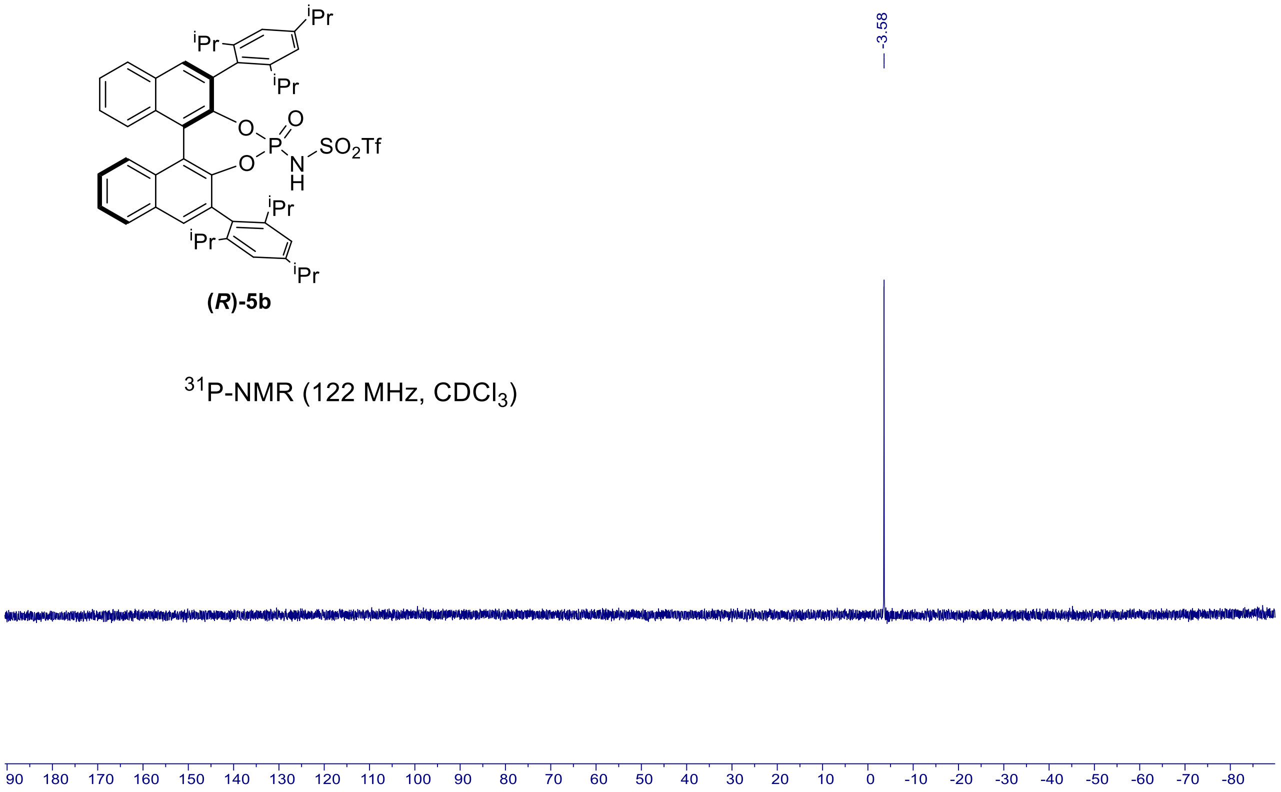

Figure SI-33. NMR spectra of compound **5b** *(continued)*.

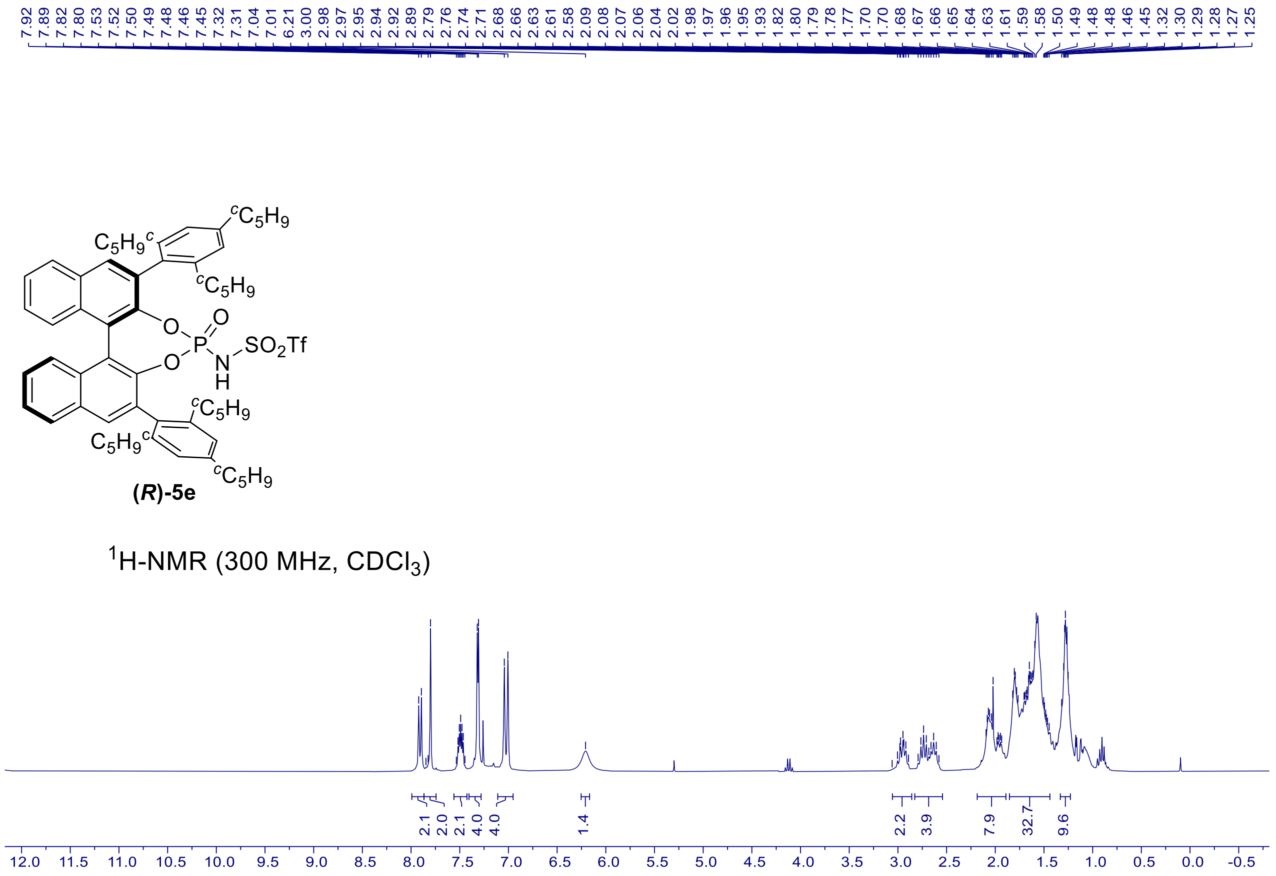

(*R*)-5e

^{1}H-NMR (300 MHz, $CDCl_3$)

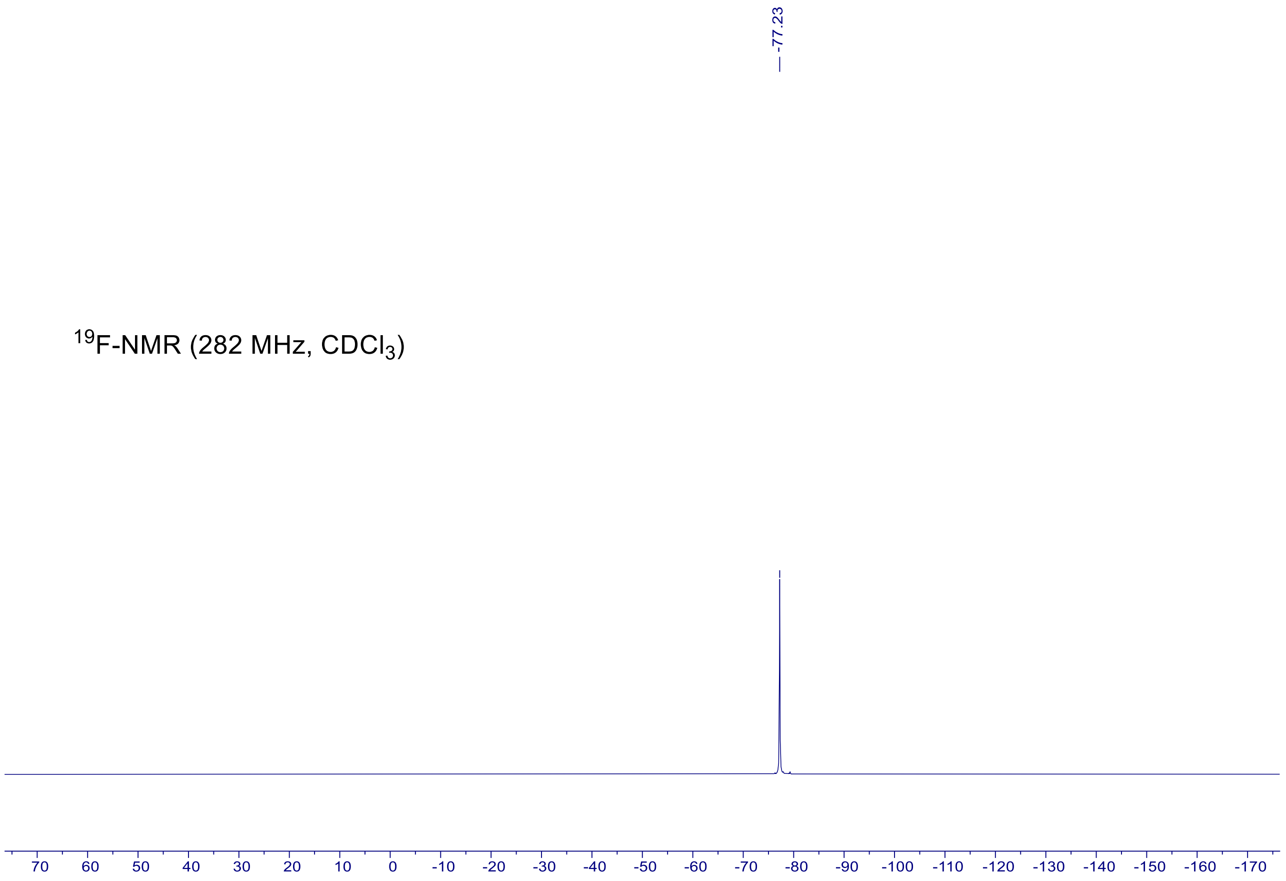

^{19}F-NMR (282 MHz, $CDCl_3$)

Figure SI-34. NMR spectra of compound **5e**.

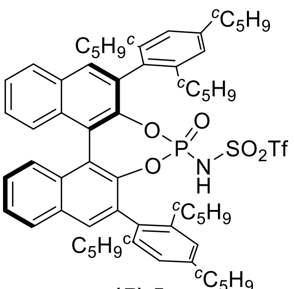

(*R*)-5e

^{31}P-NMR (122 MHz, $CDCl_3$)

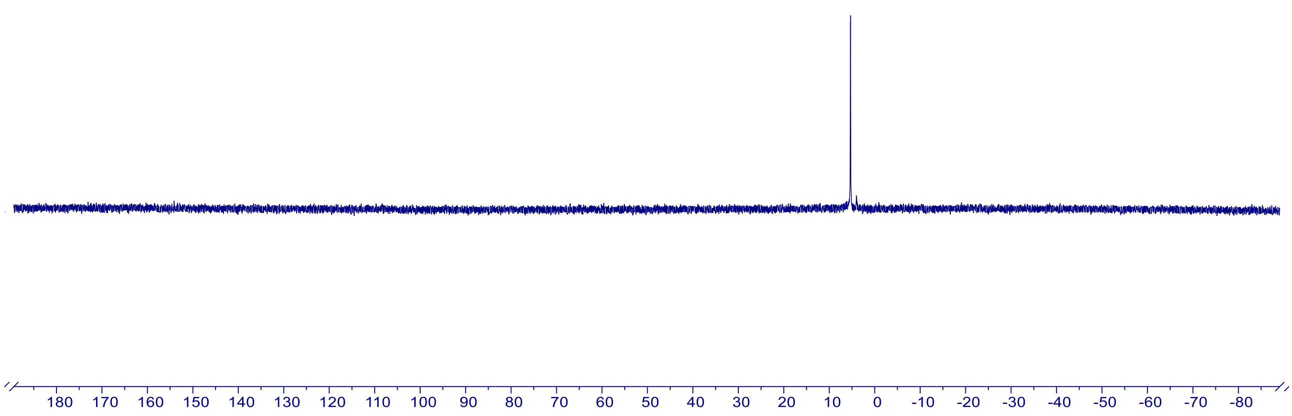

Figure SI-34. NMR spectra of compound **5e** *(continued)*.

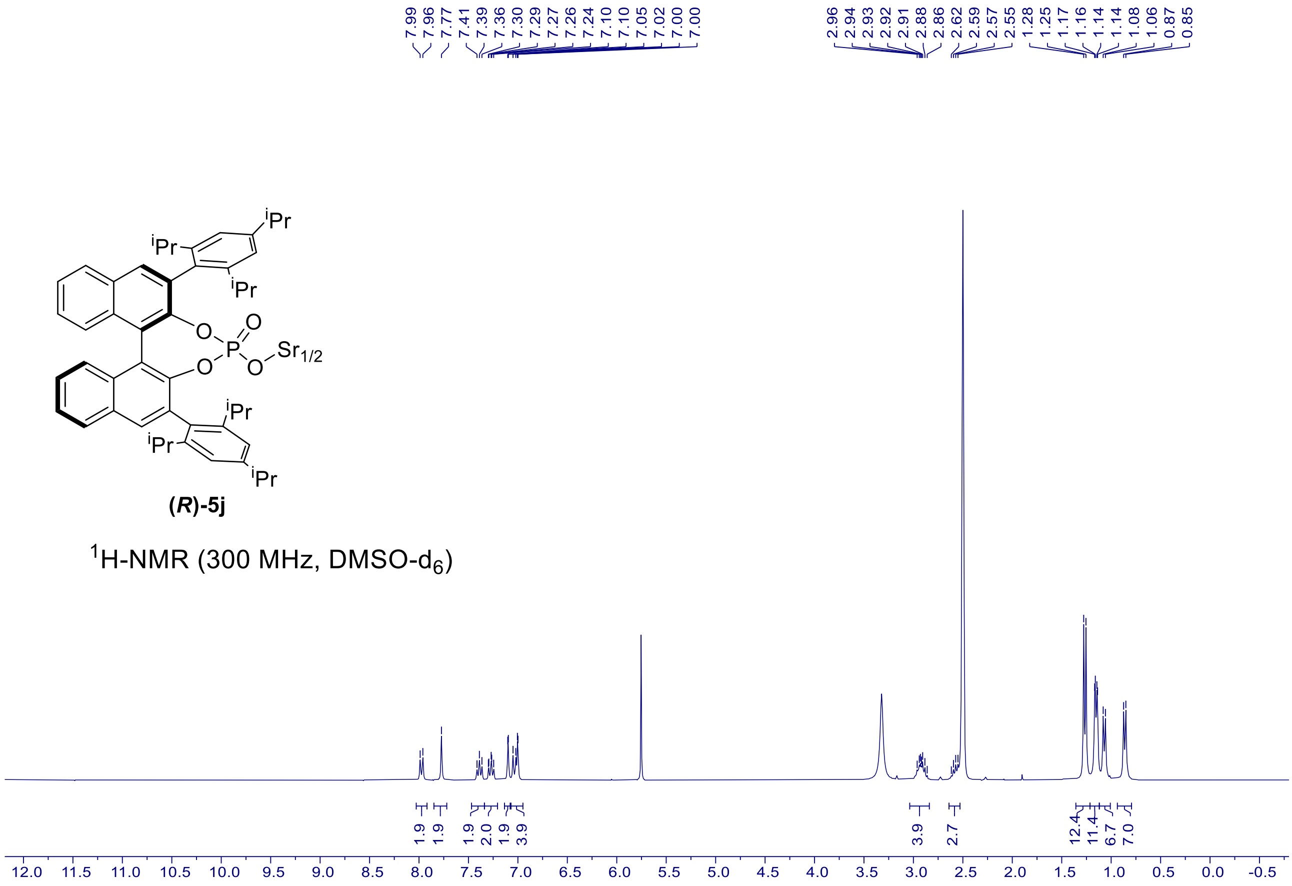

(*R*)-5j

^{1}H-NMR (300 MHz, DMSO-d$_6$)

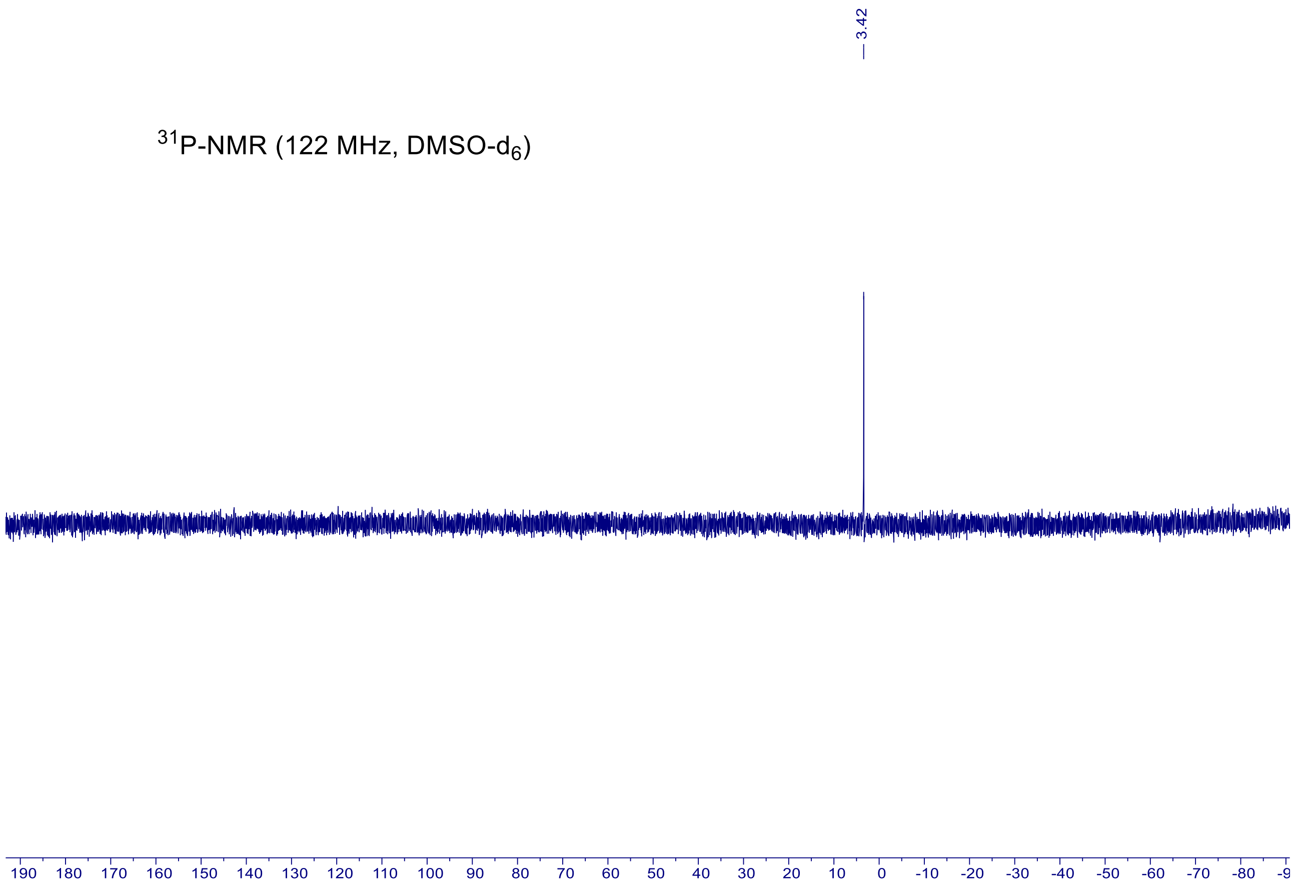

^{31}P-NMR (122 MHz, DMSO-d$_6$)

Figure SI-35. NMR spectra of compound **5j**.

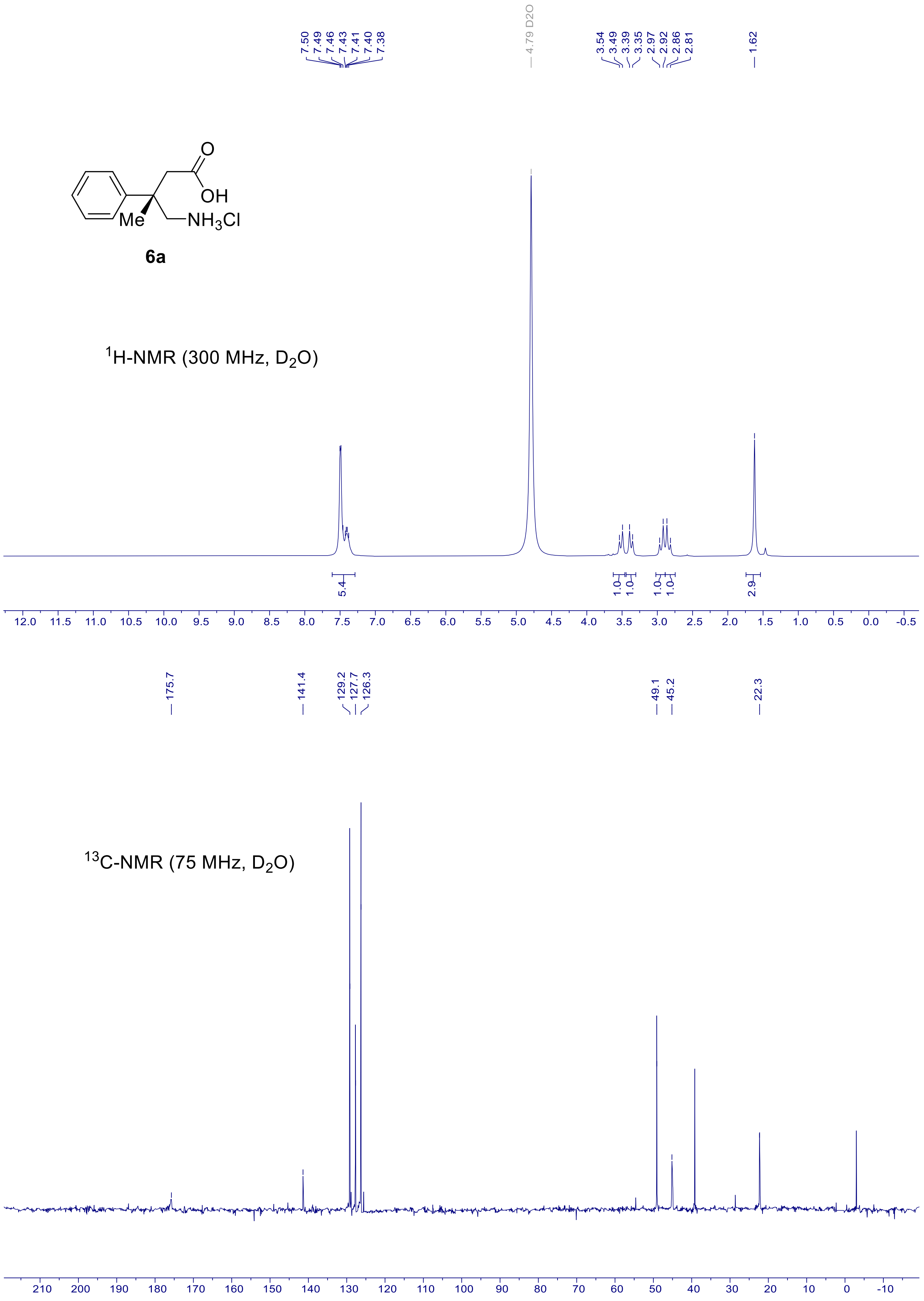

Figure SI-36. NMR spectra of compound **6a.**

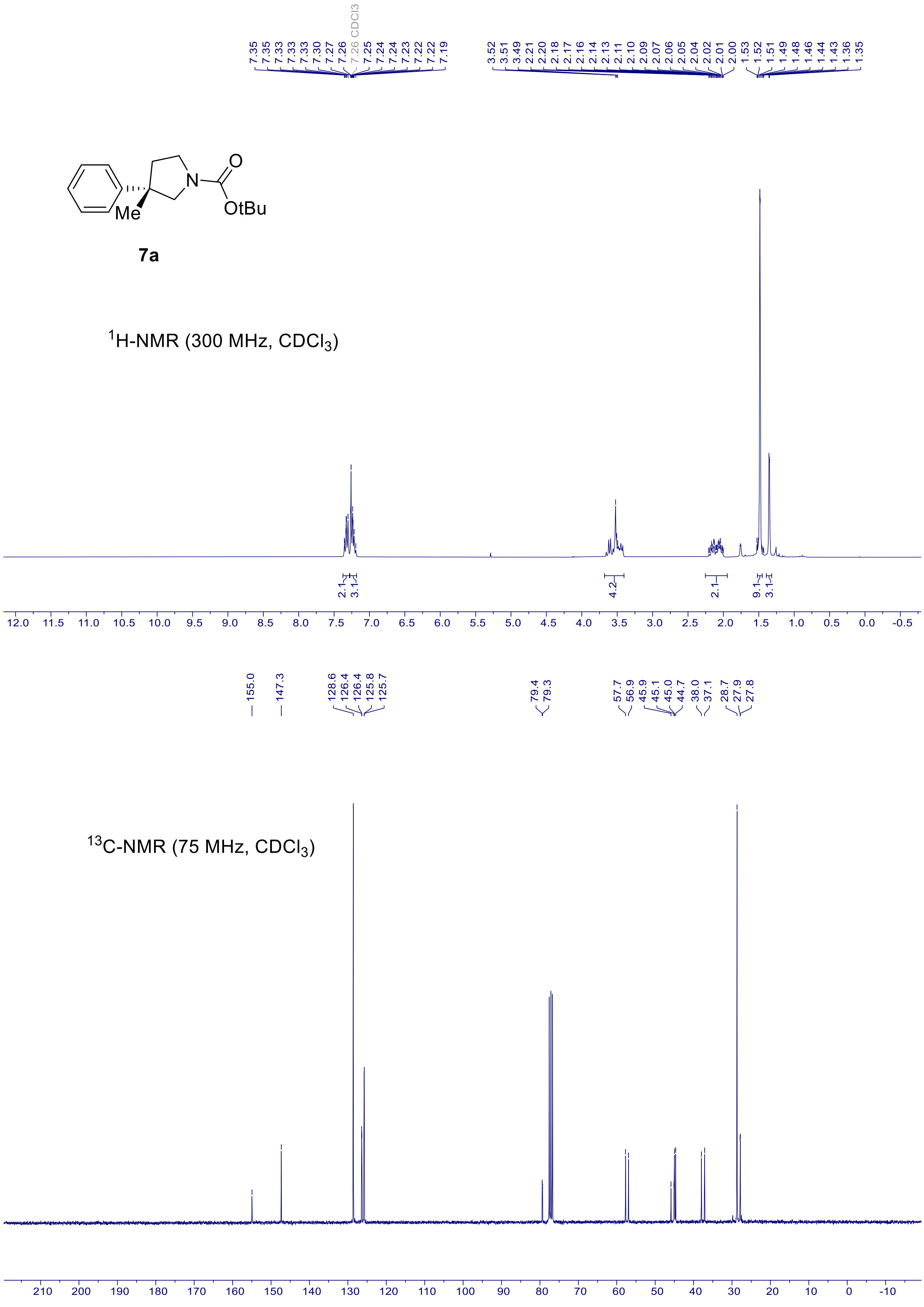

Figure SI-37. NMR spectra of compound **7a.**

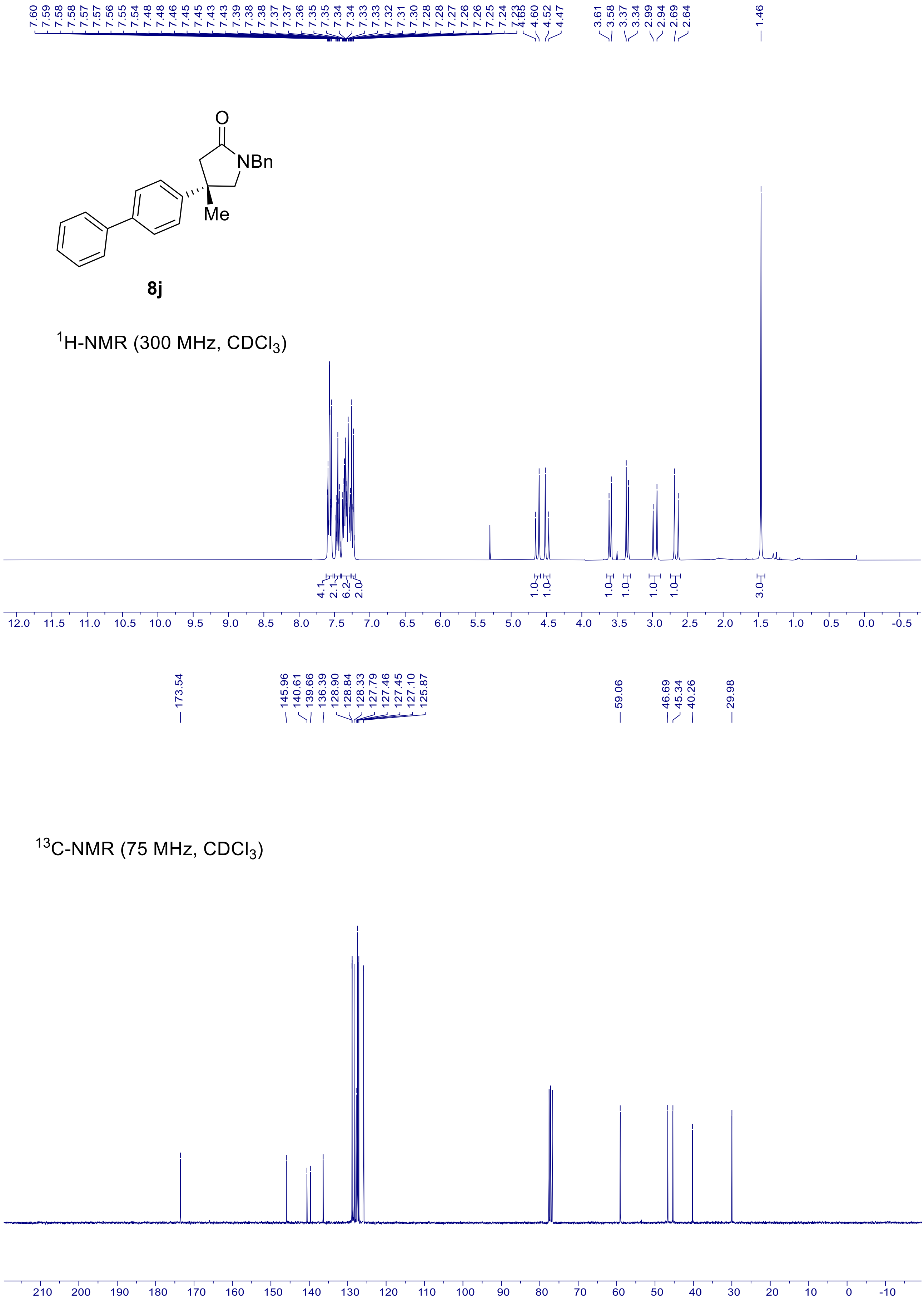

Figure SI-38. NMR spectra of compound **8j**.

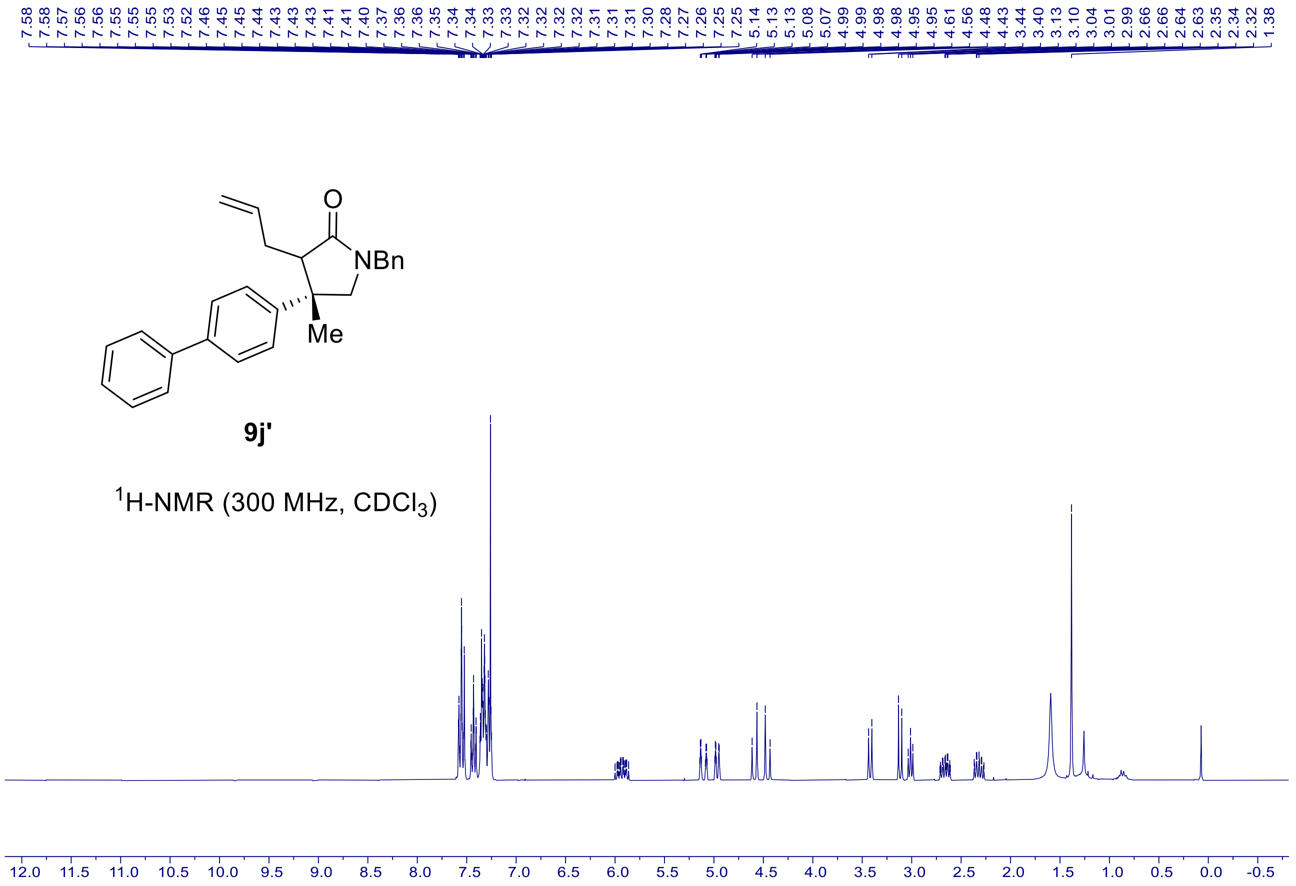

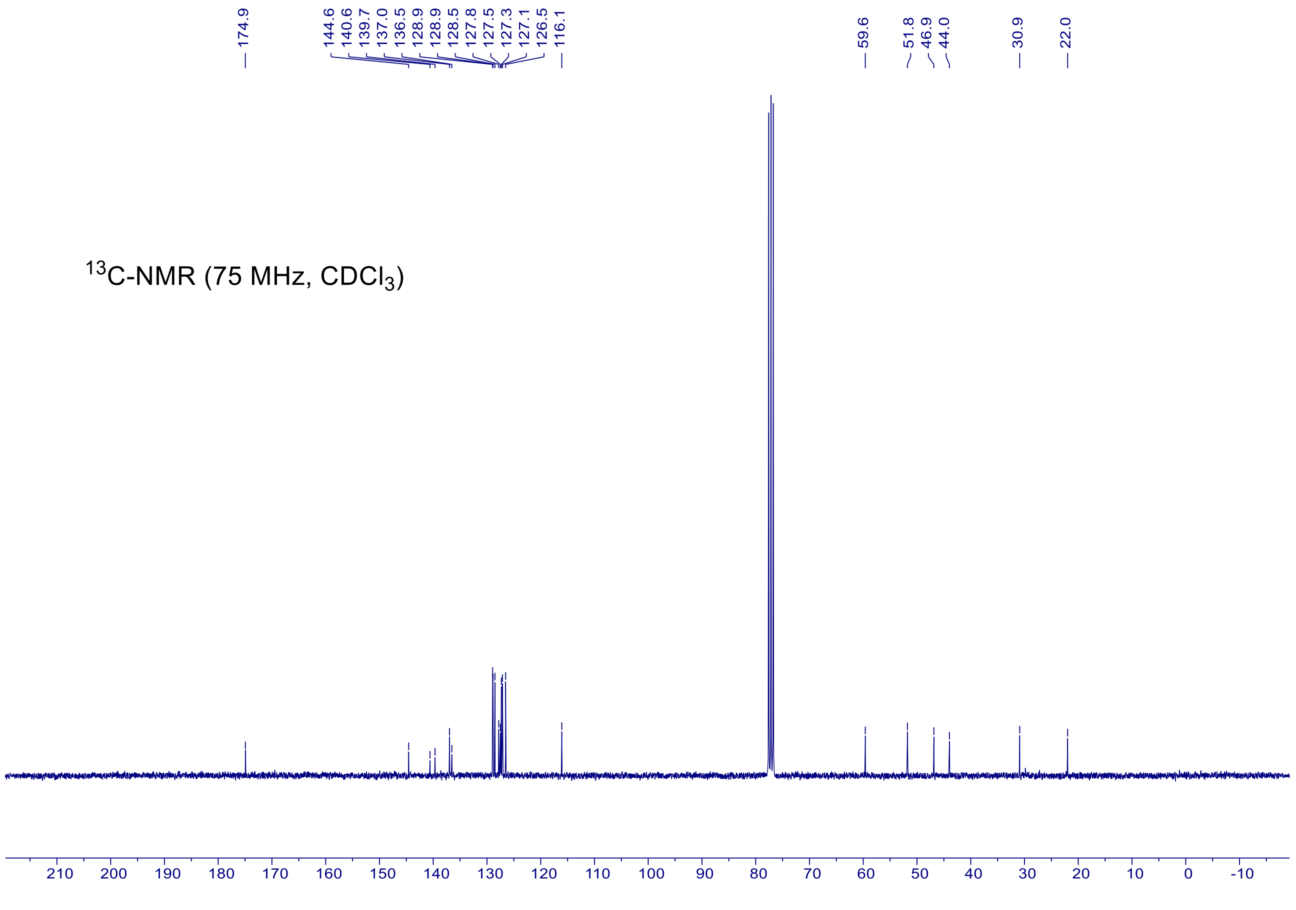

Figure SI-39. NMR spectra of compound **9j’**.

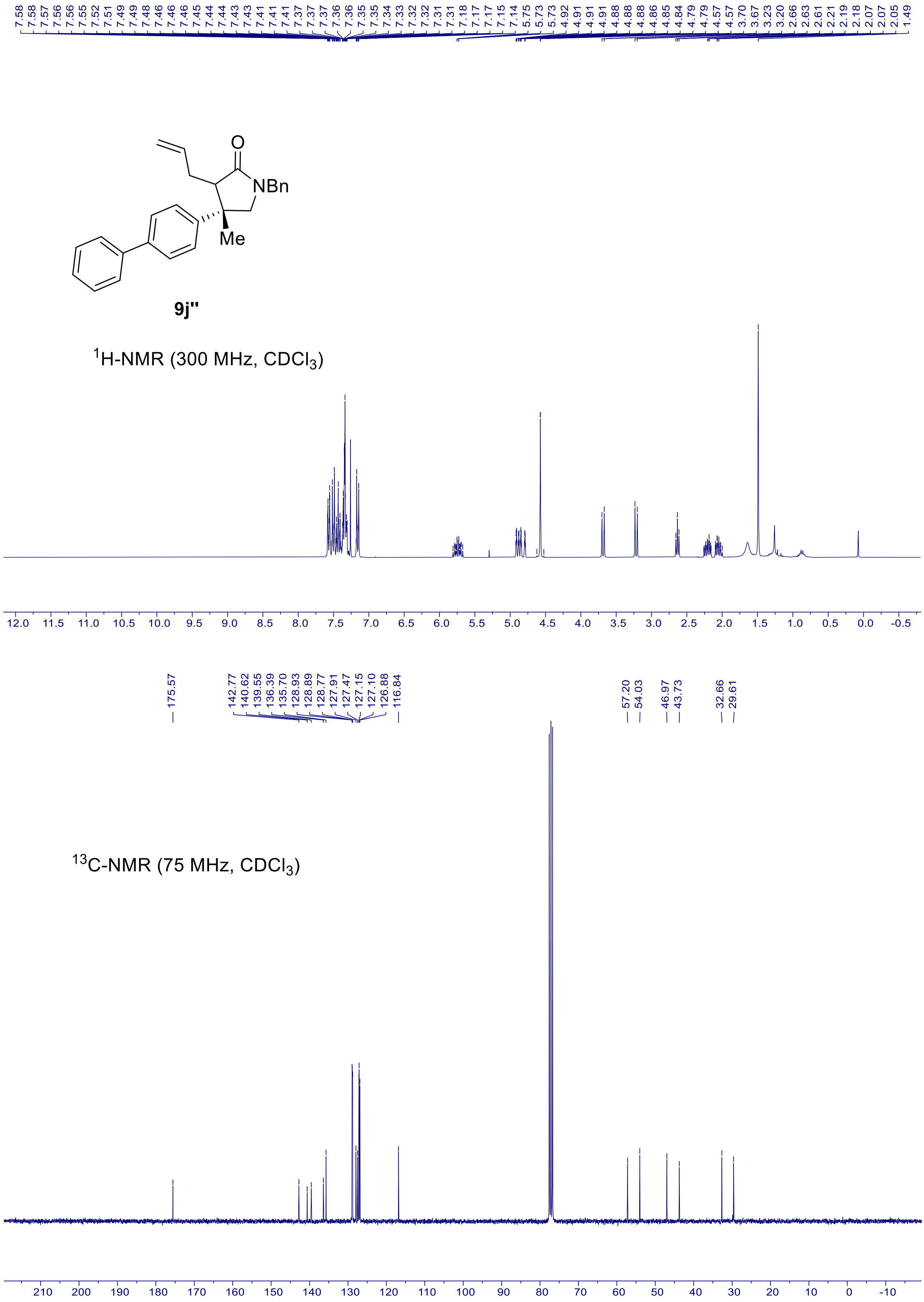

Figure SI-40. NMR spectra of compound **9j''**.

6. HPLC Traces

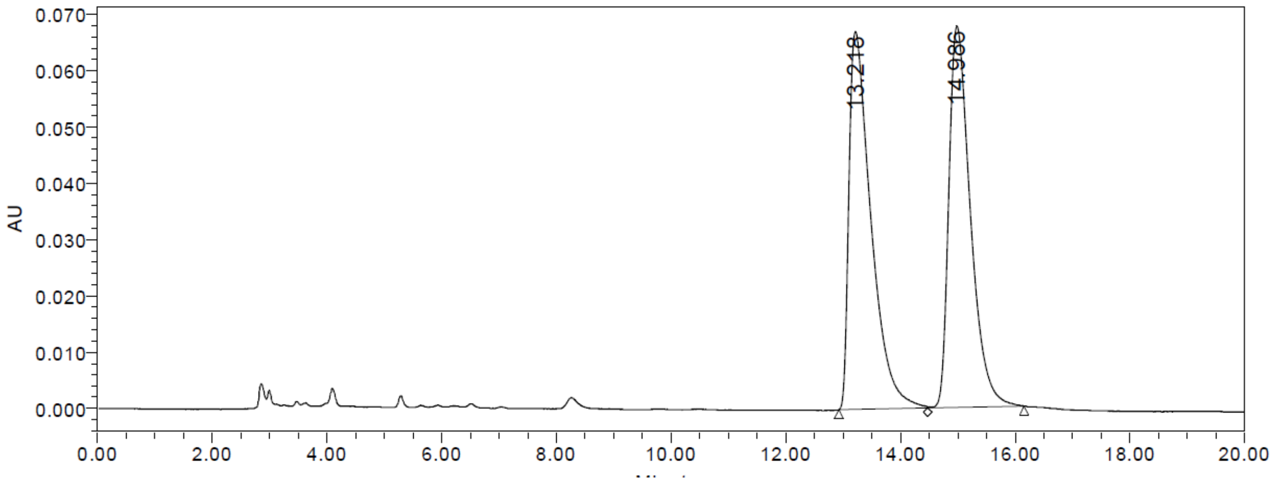

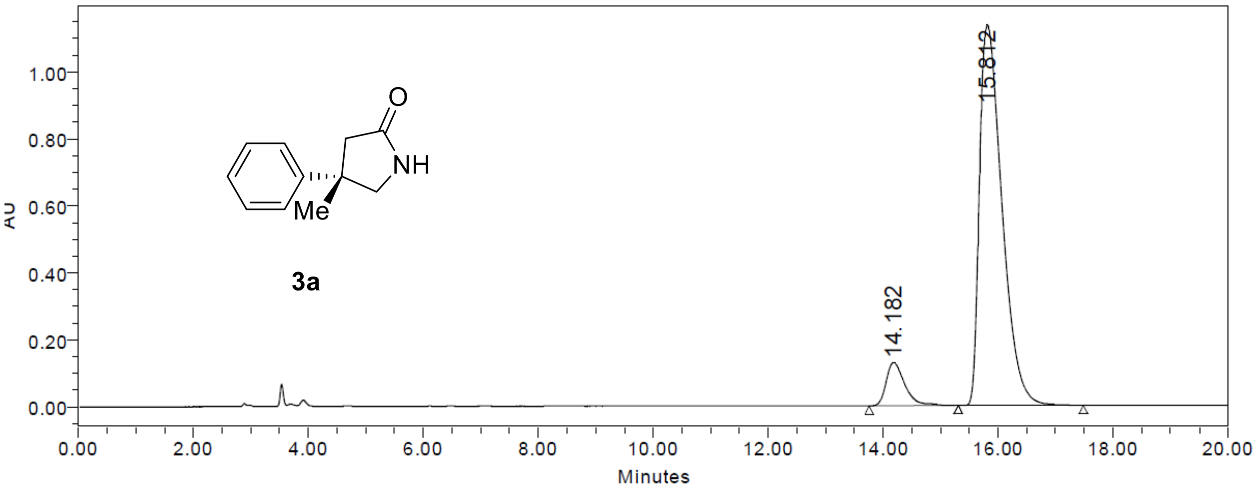

	RT	Area	% Area	Height
1	14.182	2932466	8.73	129068
2	15.812	30652722	91.27	1136287

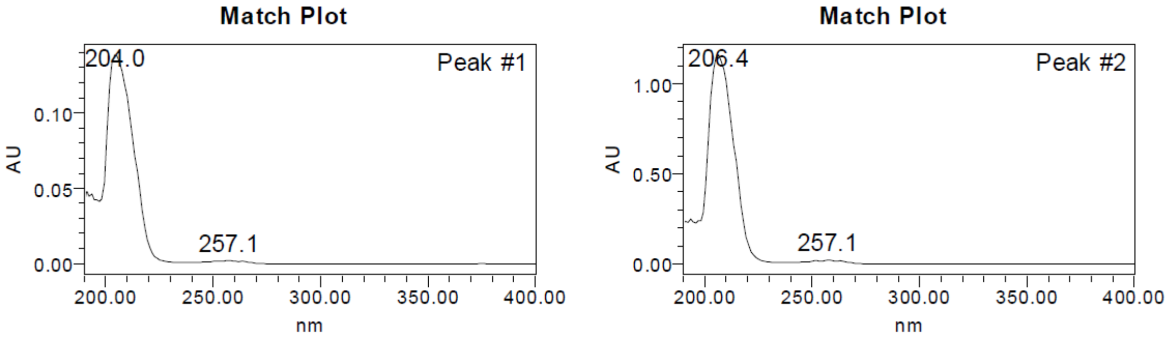

Figure SI-41. HPLC traces of compound **3a**.

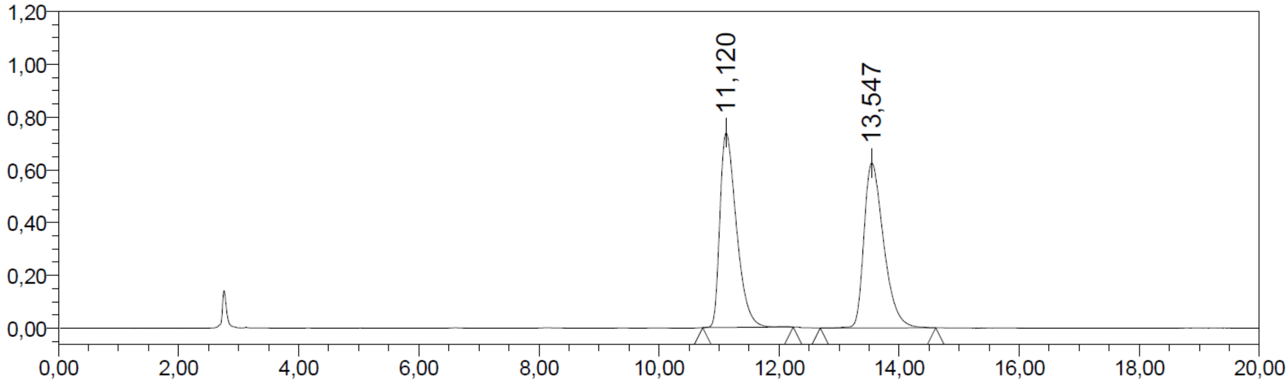

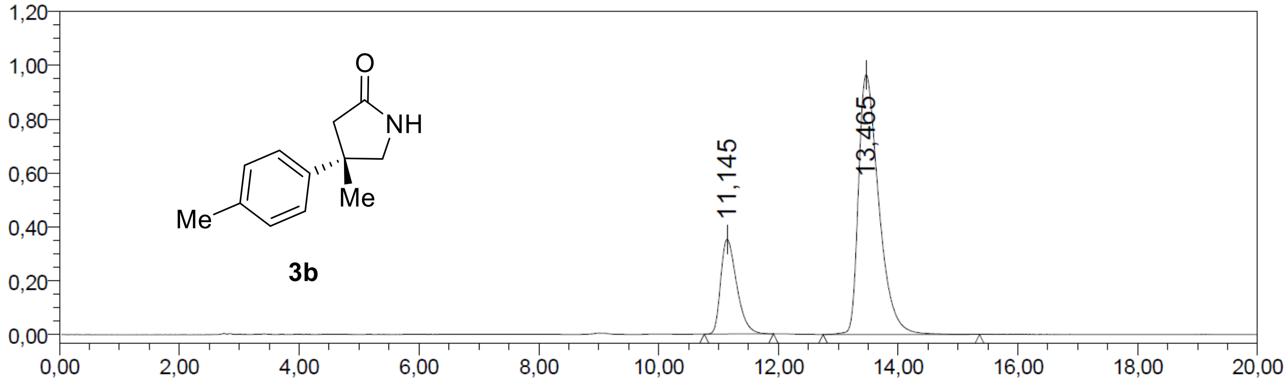

Peak Results

	Name	RT	Area	Height	% Area
1		11,145	6520938	351543	22,30
2		13,465	22720020	964018	77,70

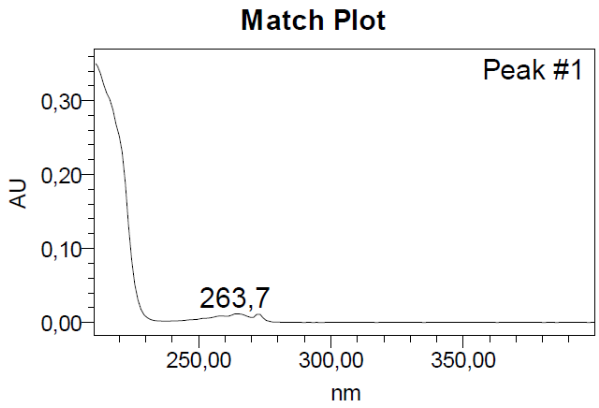

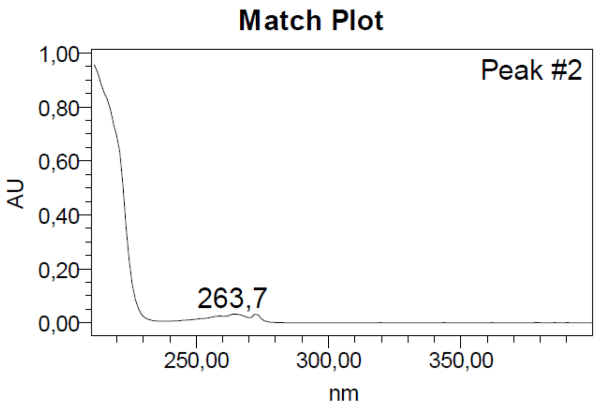

Figure SI-42. HPLC traces of compound **3b**.

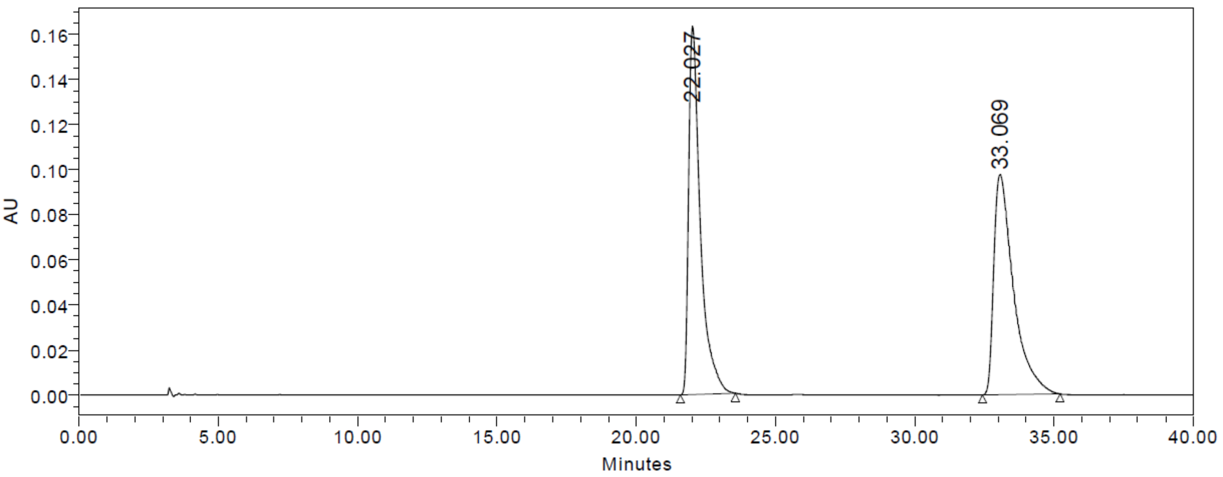

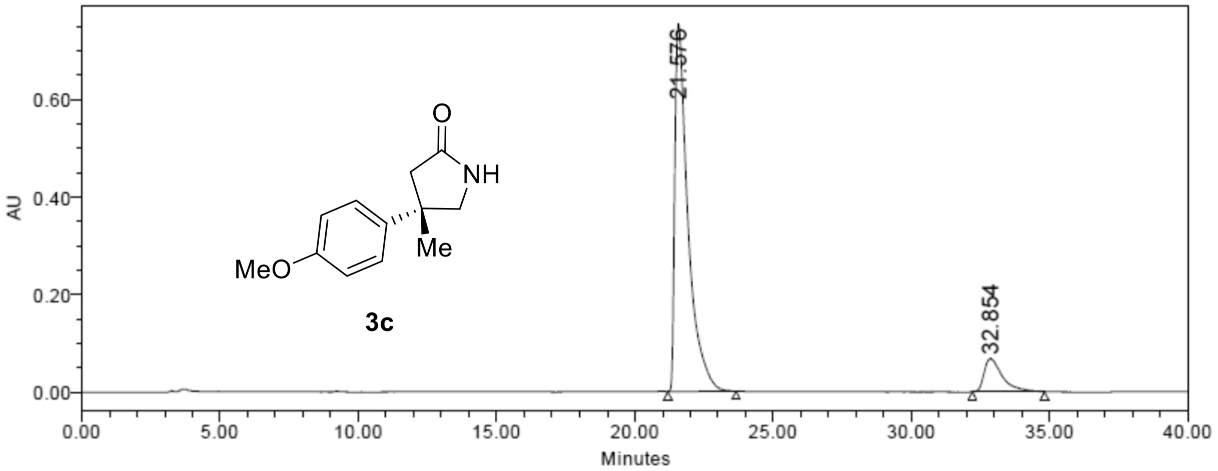

	RT	Area	% Area	Height
1	21.576	25205968	88.99	753388
2	32.854	3119543	11.01	68006

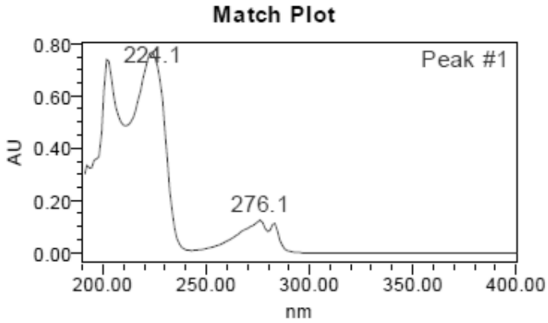

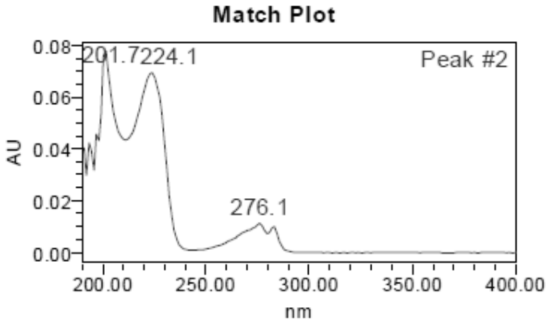

Figure SI-43. HPLC traces of compound **3c**.

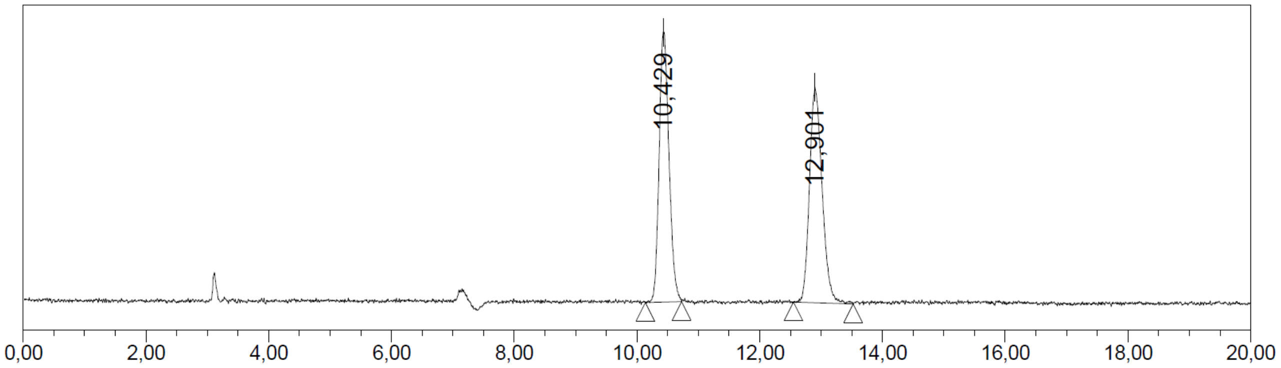

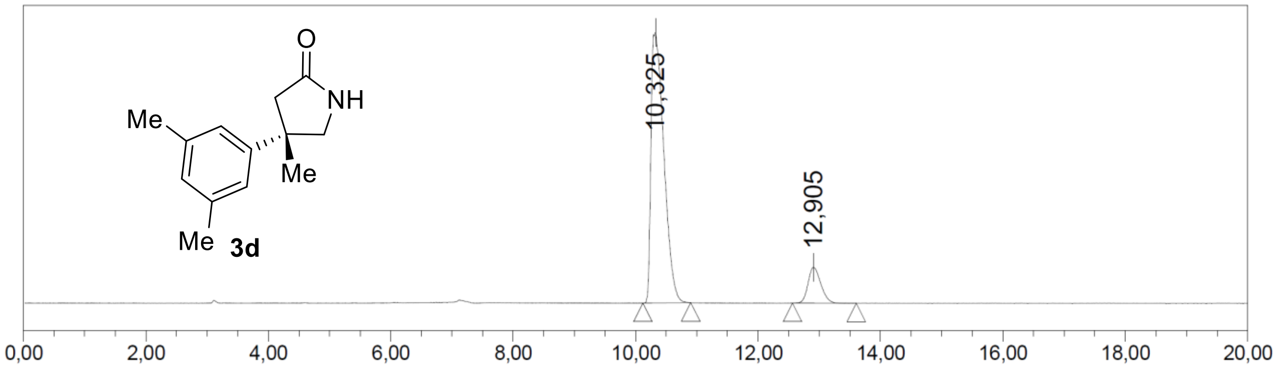

Peak Results

	Name	RT	Area	Height	% Area
1		10,325	22396878	1541797	88,30
2		12,905	2968897	204344	11,70

Match Plot

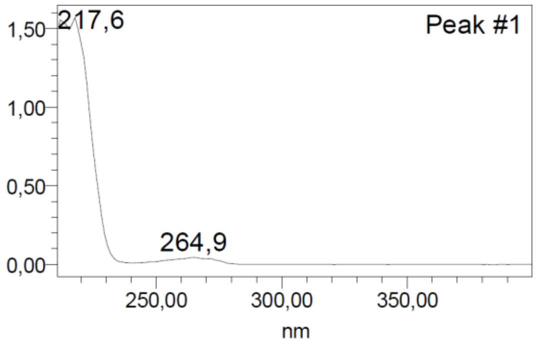

Match Plot

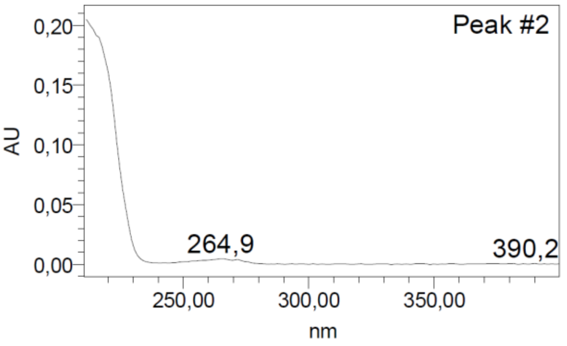

Figure SI-44. HPLC traces of compound **3d**.

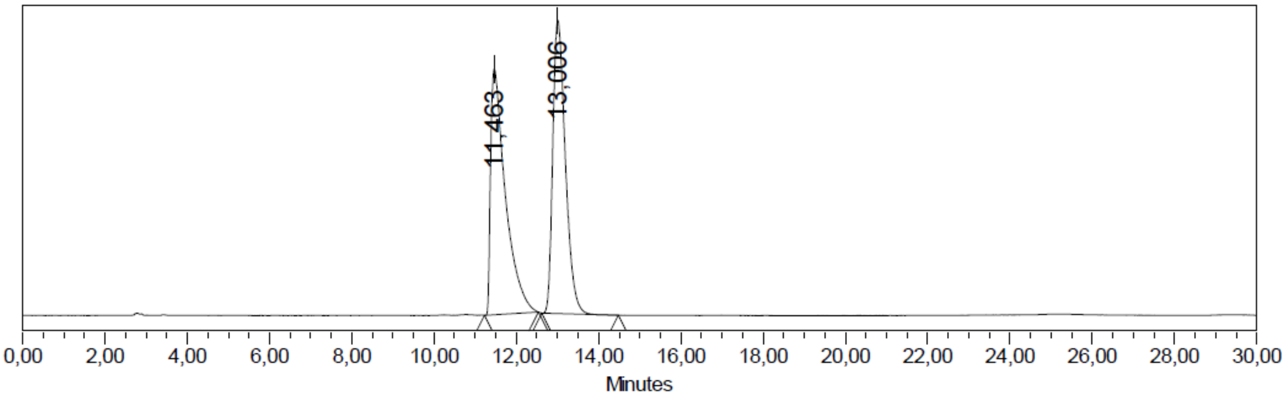

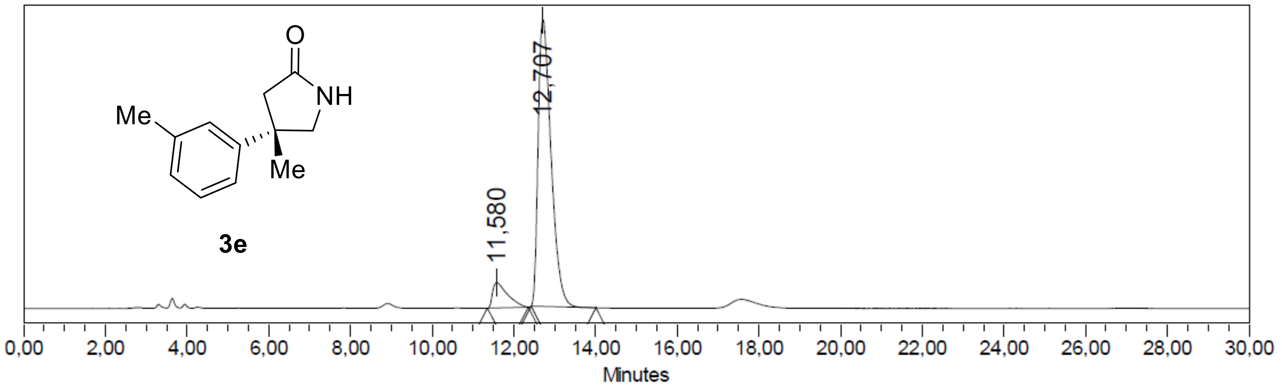

Peak Results

	Name	RT	Area	Height	% Area
1		11,580	3351483	132217	9,27
2		12,707	32808371	1494404	90,73

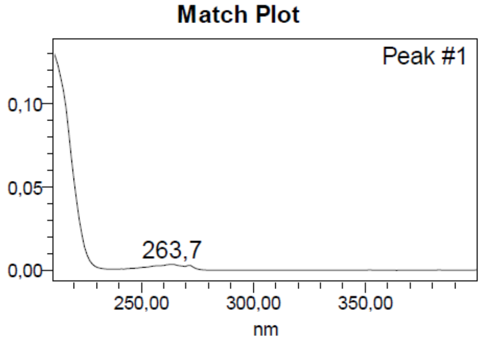

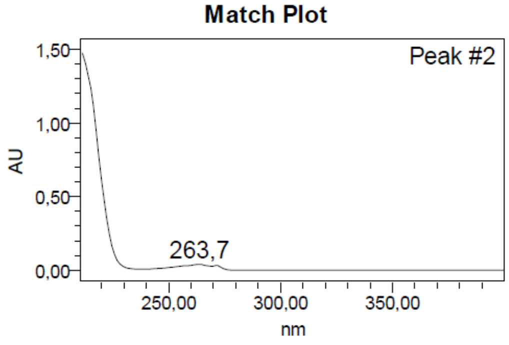

Figure SI-45. HPLC traces of compound **3e**.

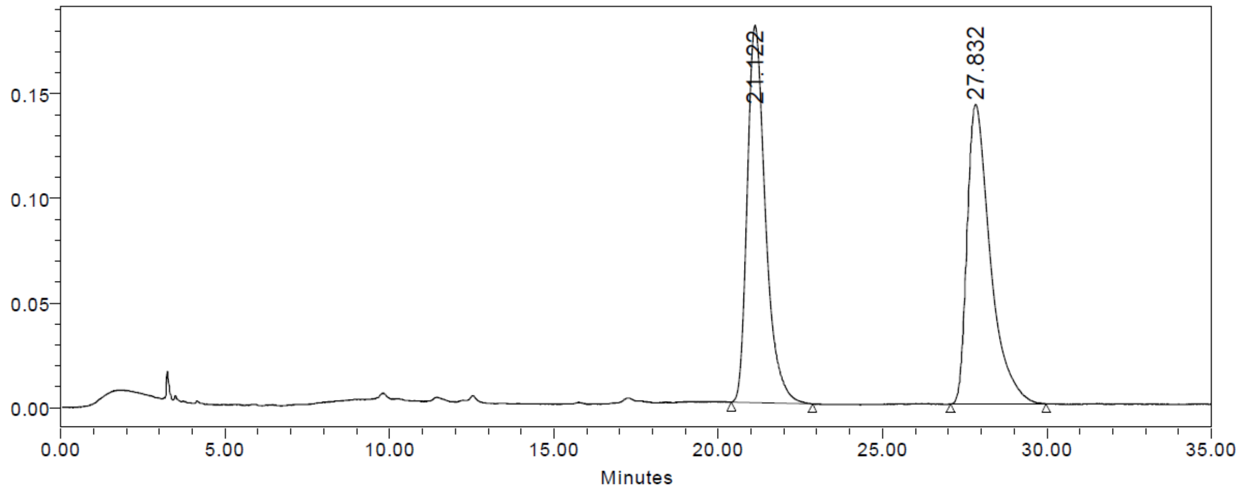

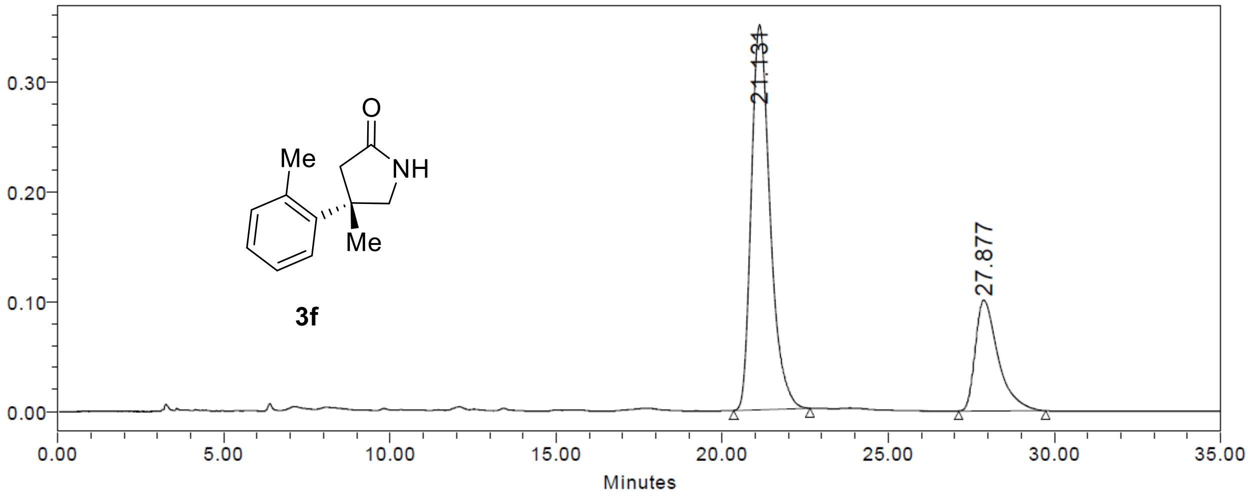

	RT	Area	% Area	Height
1	21.131	13868721	74.30	349780
2	27.877	4796271	25.70	100626

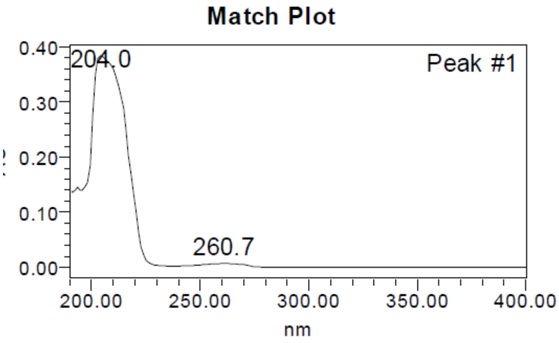

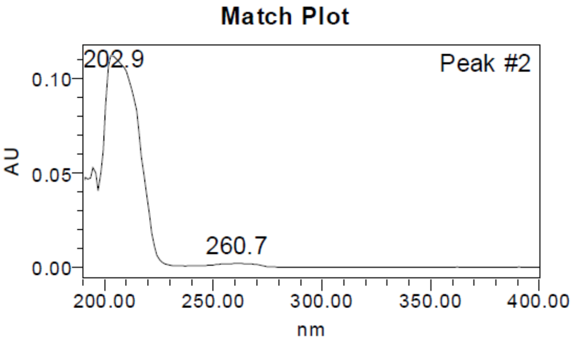

Figure SI-46. HPLC traces of compound **3f**.

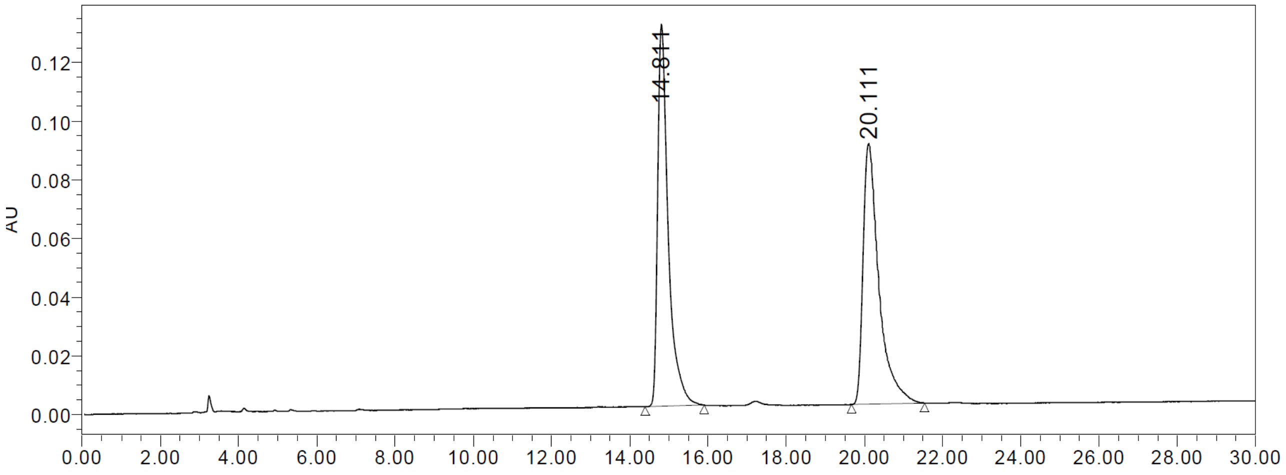

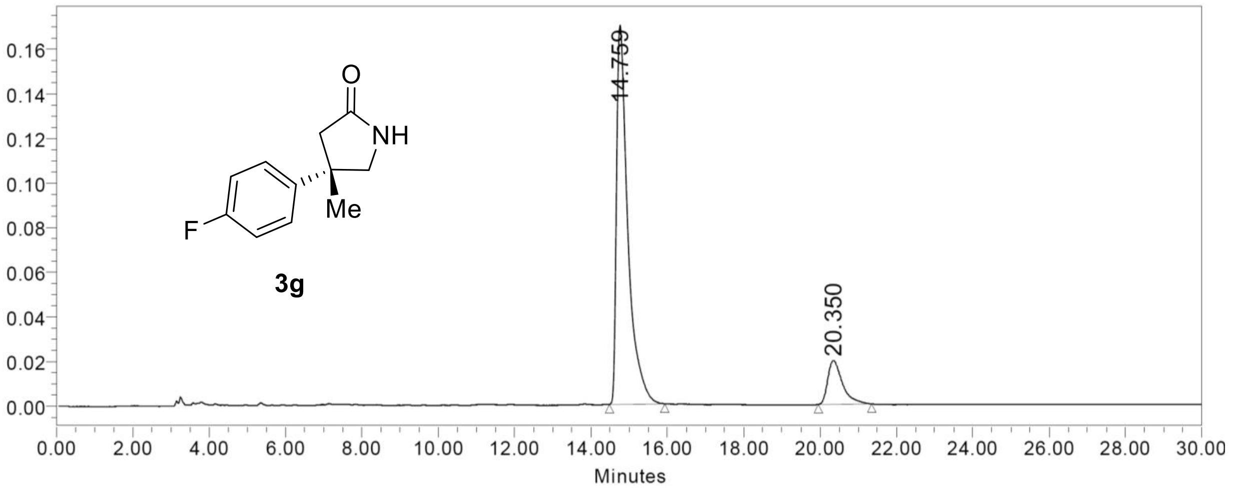

	RT	Area	% Area	Height
1	14.759	3527359	87.12	169810
2	20.350	521574	12.88	19602

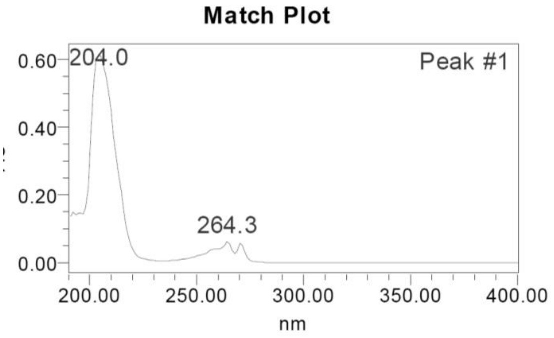

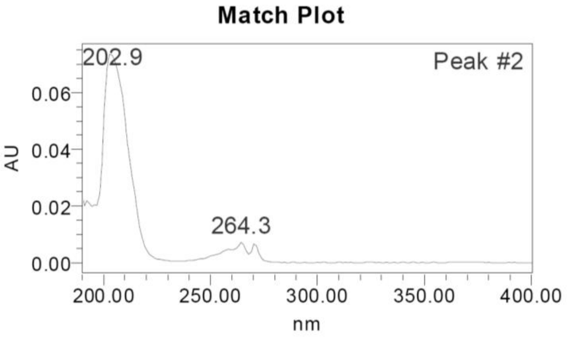

Figure SI-47. HPLC traces of compound **3g**.

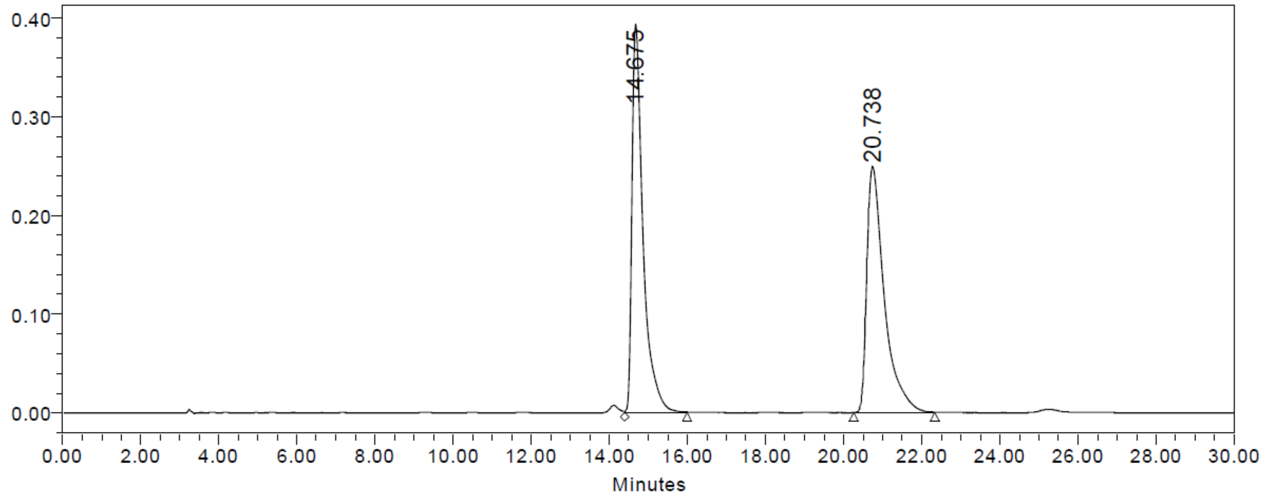

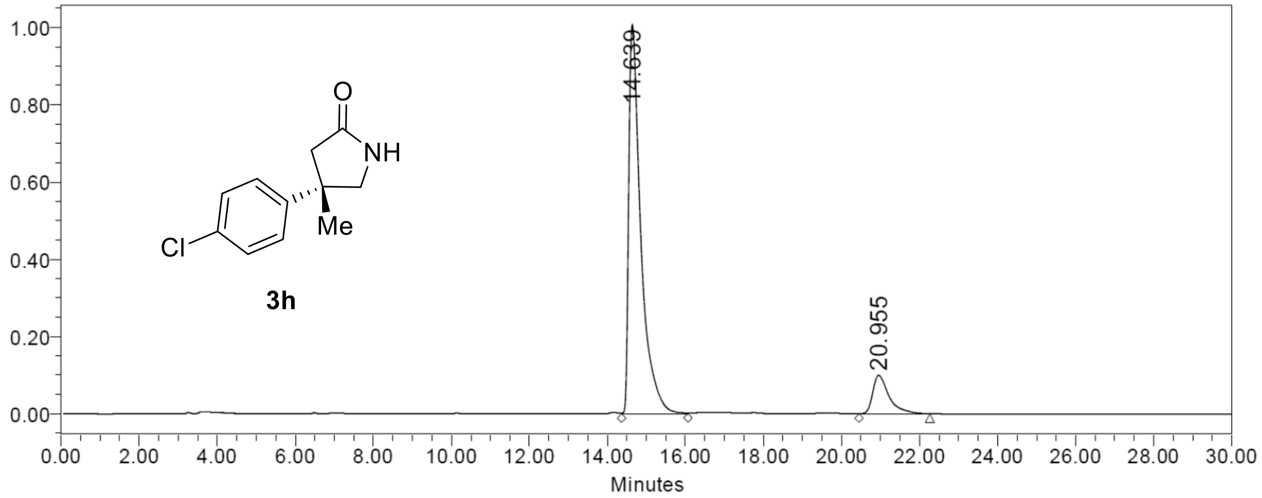

	RT	Area	% Area	Height
1	14.639	21852925	88.31	1008398
2	20.955	2891996	11.69	99661

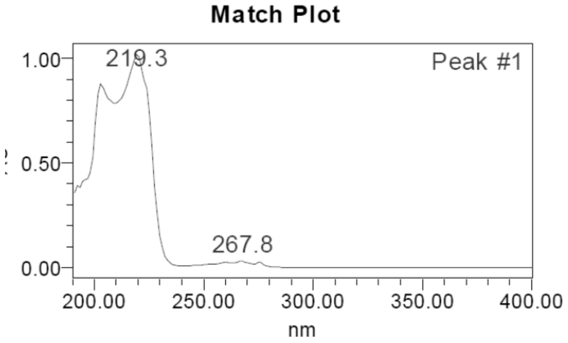

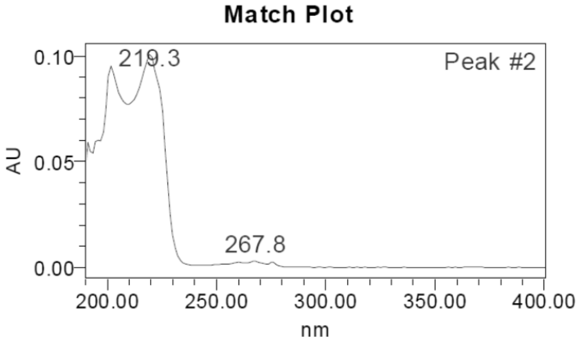

Figure SI-48. HPLC traces of compound **3h**.

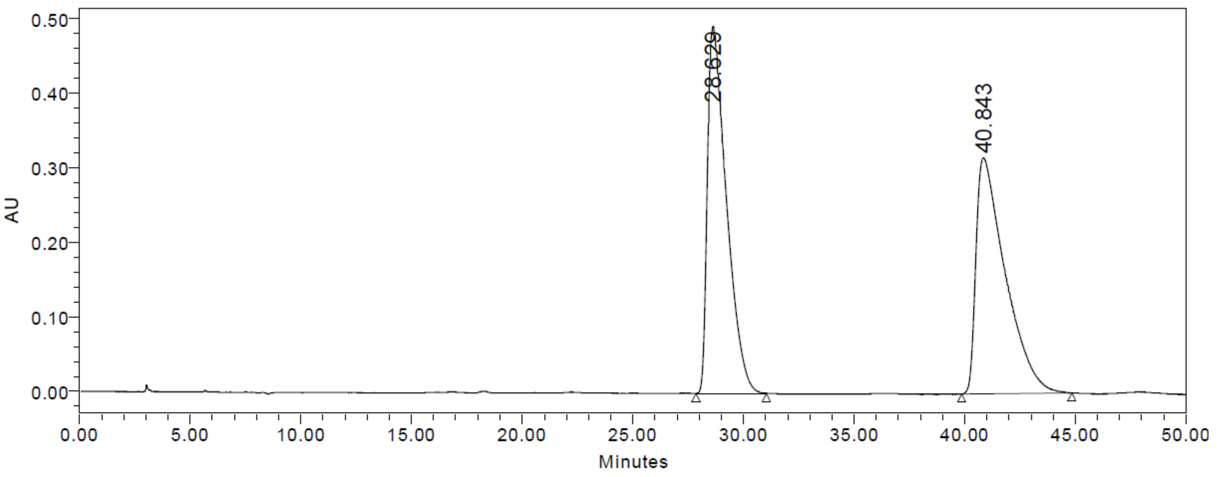

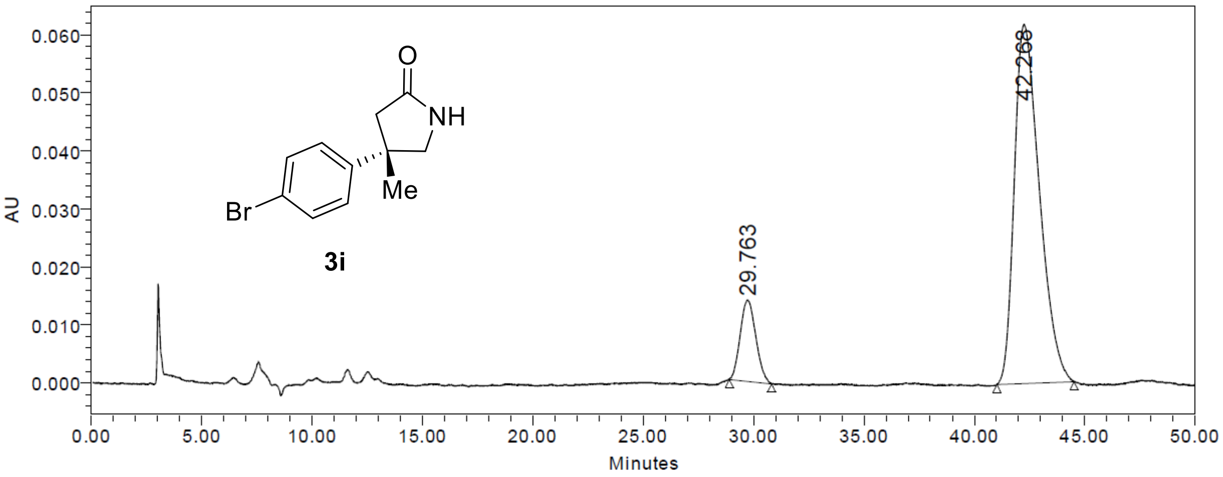

	RT	Area	% Area	Height
1	29.763	692715	12.40	14075
2	42.268	4893602	87.60	61929

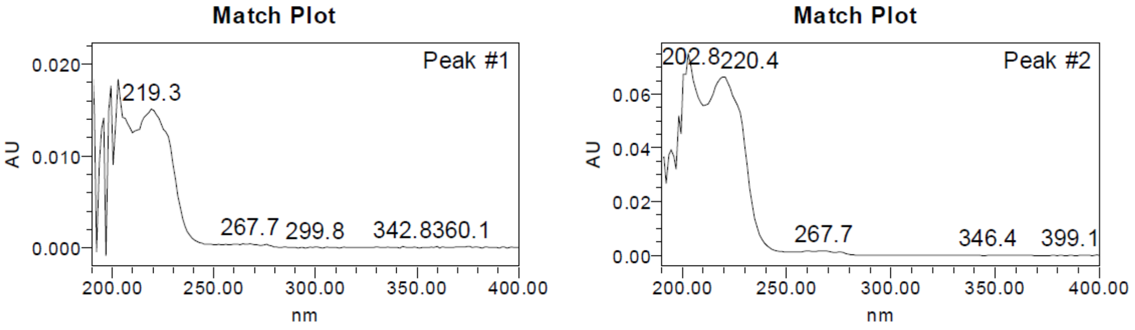

Figure SI-49. HPLC traces of compound **3i**.

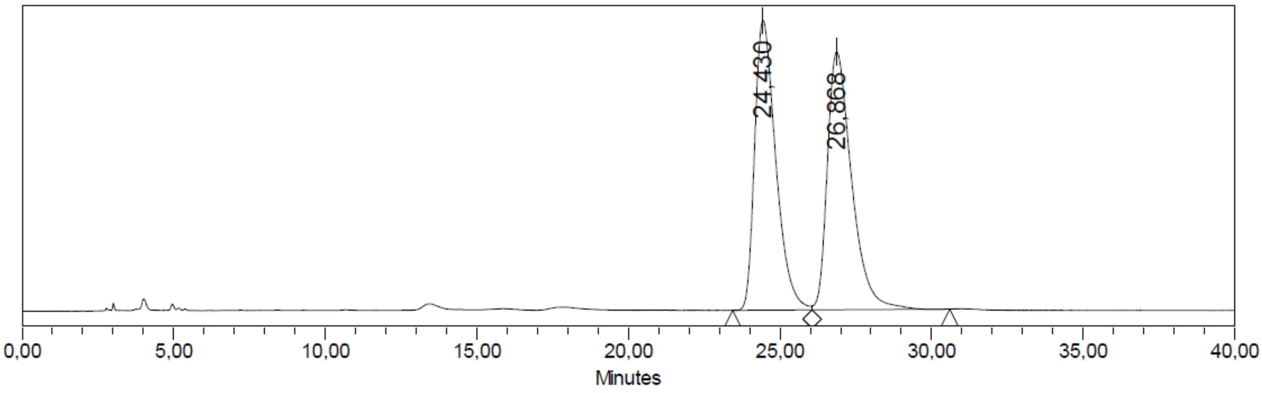

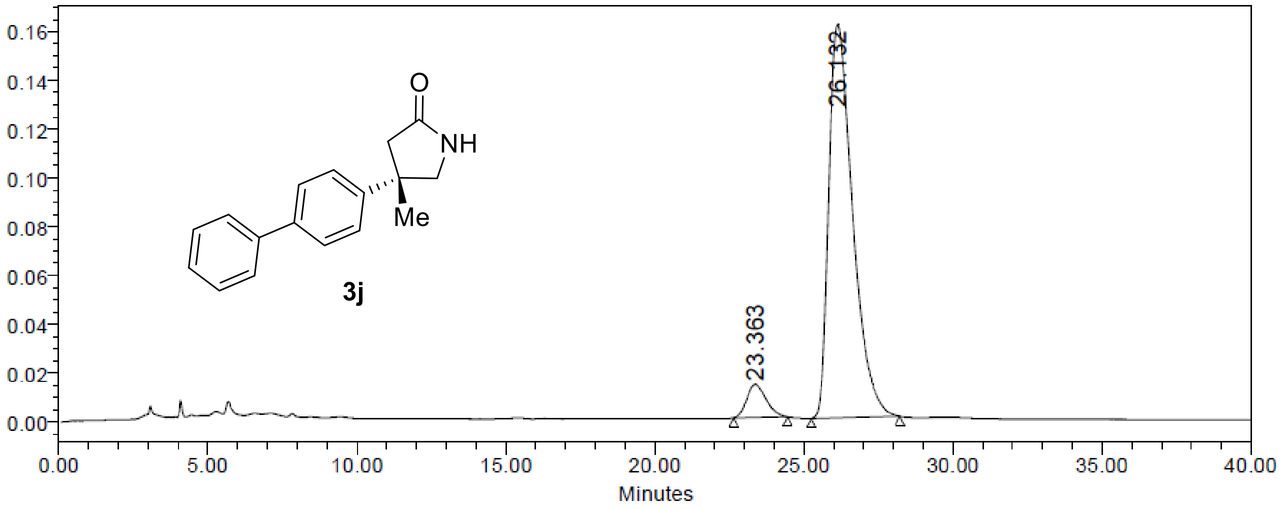

	RT	Area	% Area	Height
1	23.363	614706	6.36	13580
2	26.132	9046427	93.64	161036

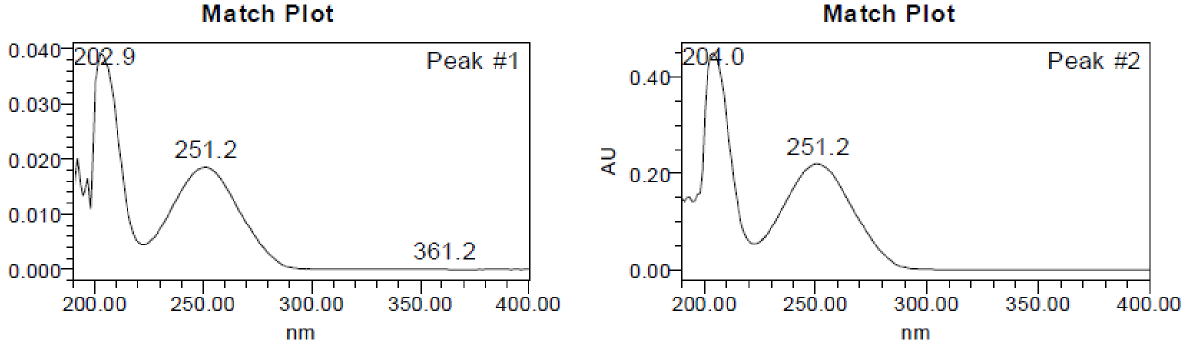

Figure SI-50. HPLC traces of compound **3j**.

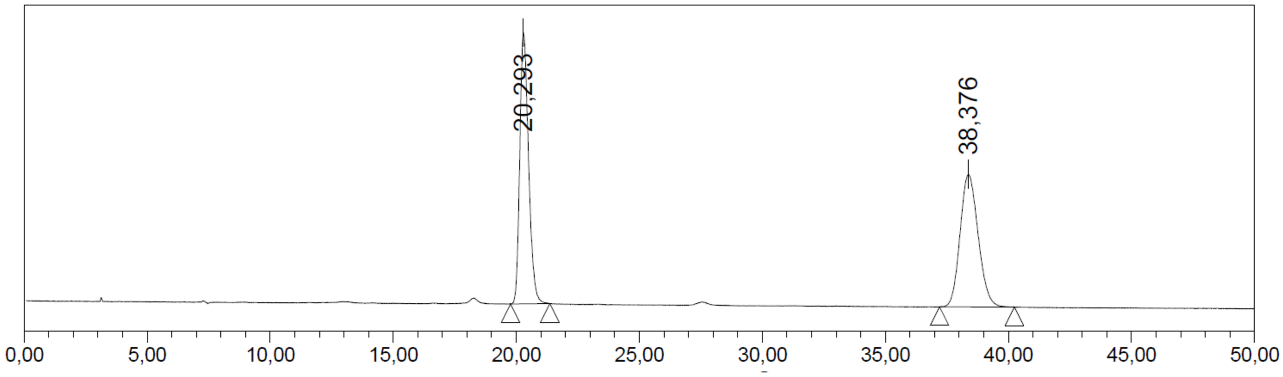

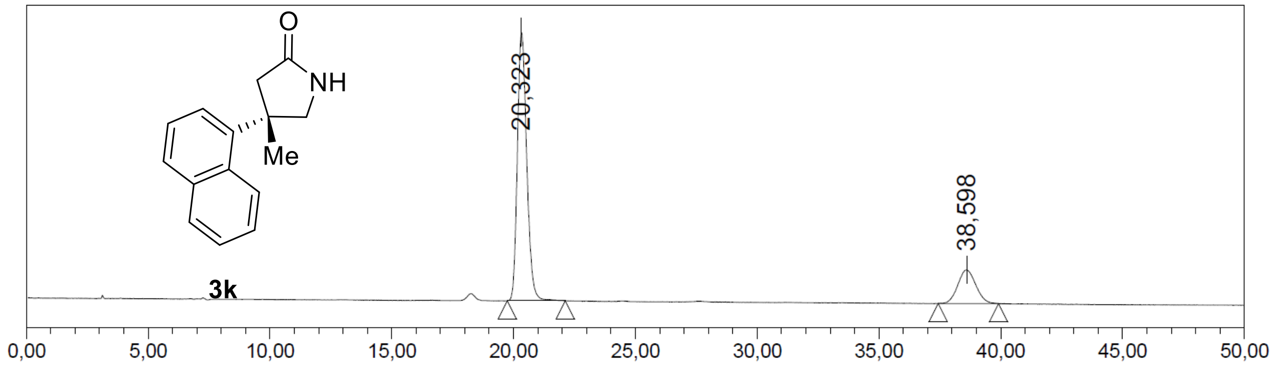

Peak Results

	Name	RT	Area	Height	% Area
1		20,323	11851296	443242	80,59
2		38,598	2854290	55670	19,41

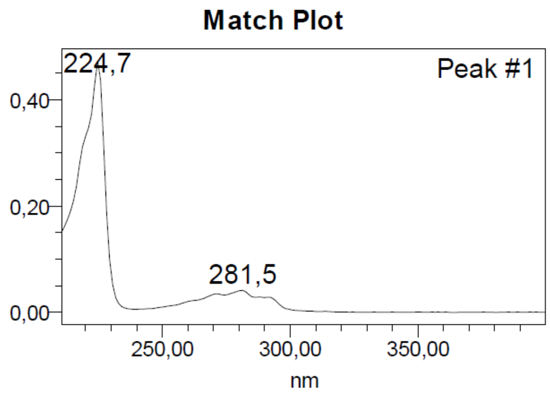

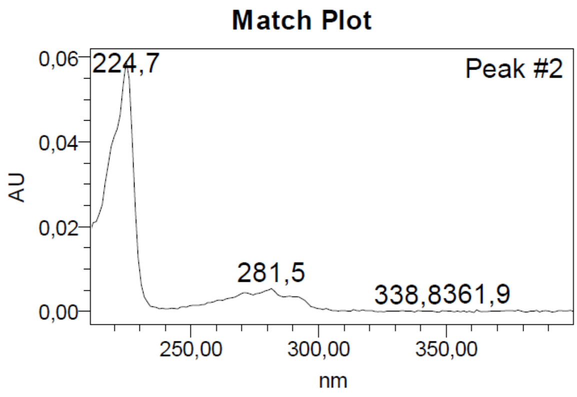

Figure SI-51. HPLC traces of compound **3k**.

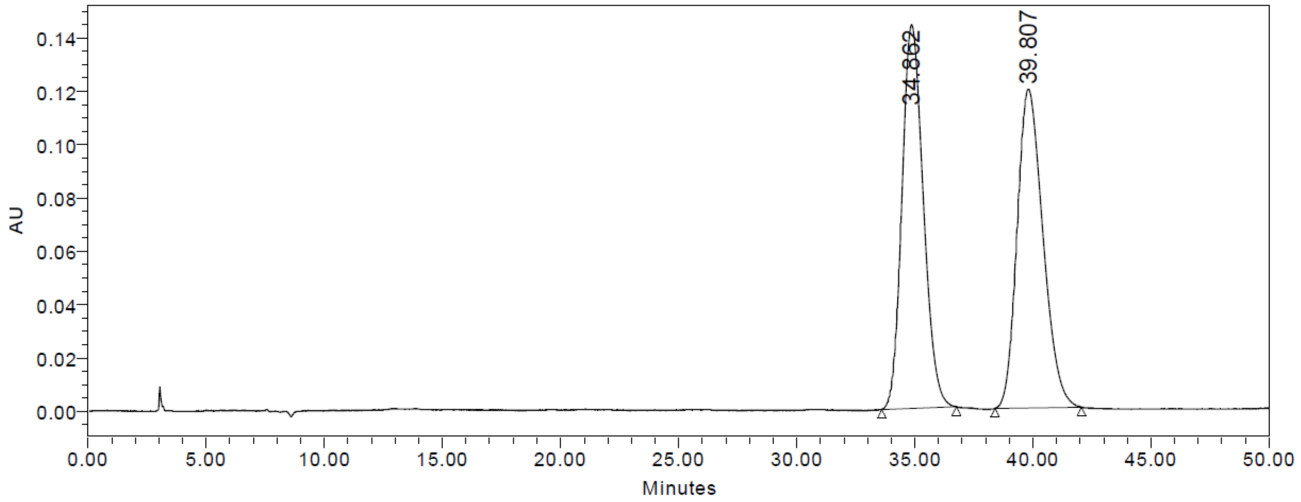

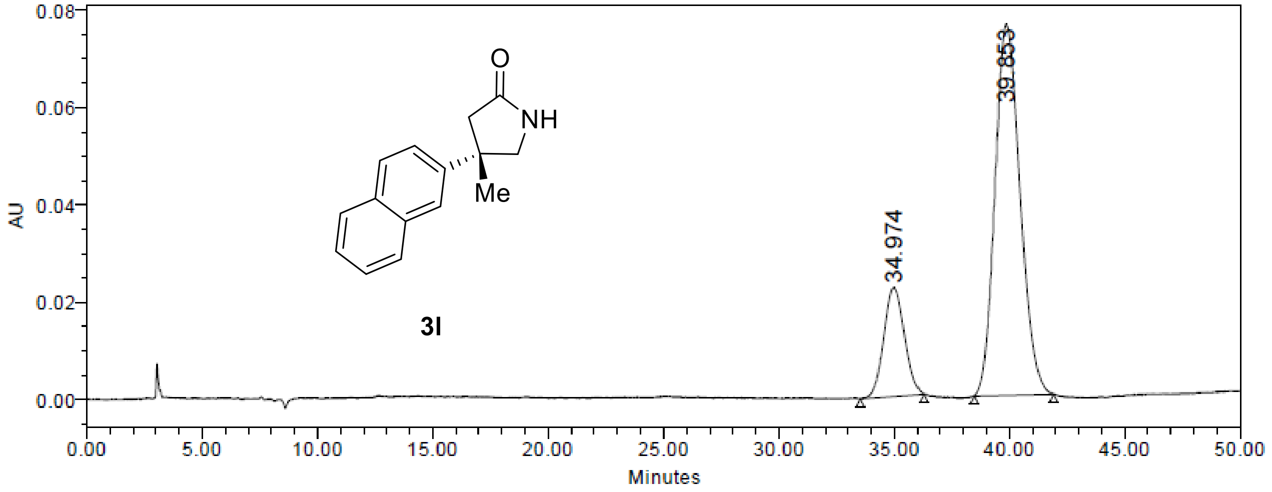

	RT	Area	% Area	Height
1	34.974	1391933	19.51	22426
2	39.853	5742252	80.49	76142

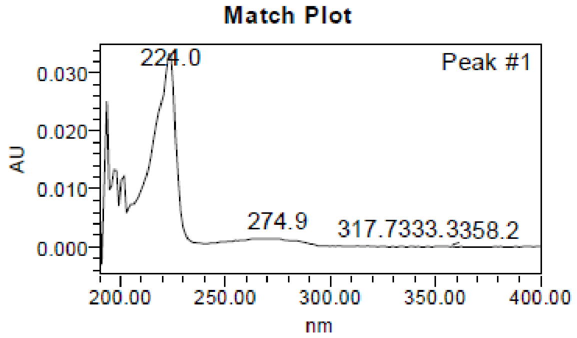

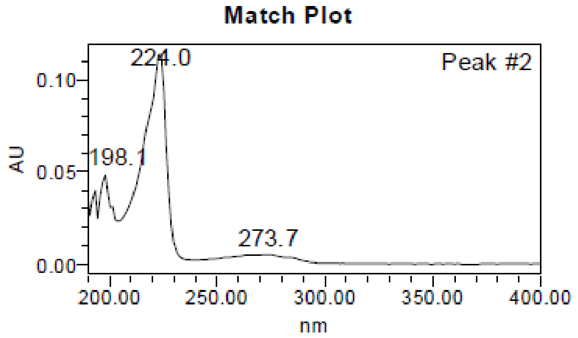

Figure SI-52. HPLC traces of compound **3l**.

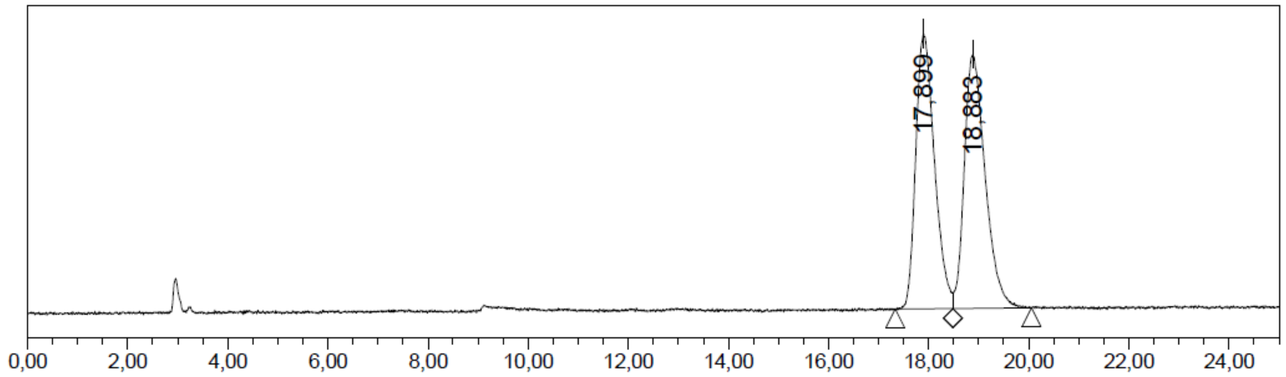

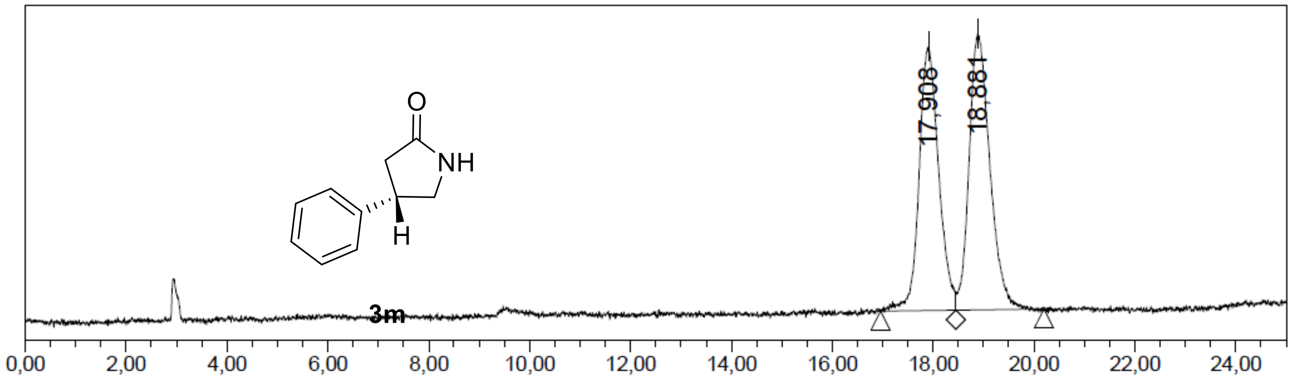

Peak Results

	Name	RT	Area	Height	% Area
1		17,908	3305428	122759	47,35
2		18,881	3675205	128185	52,65

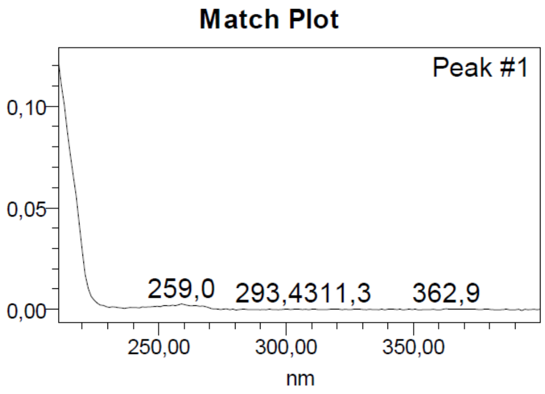

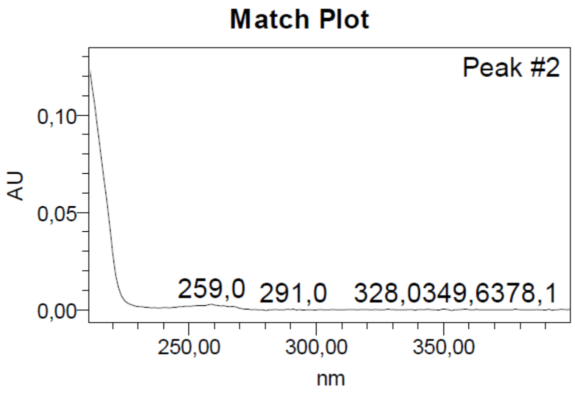

Figure SI-53. HPLC traces of compound **3m**.

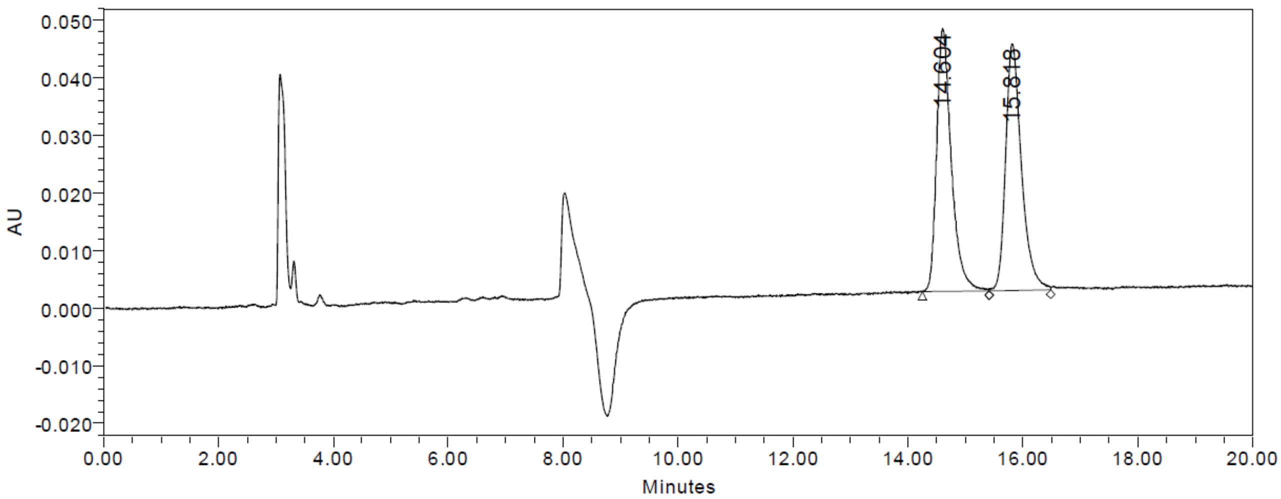

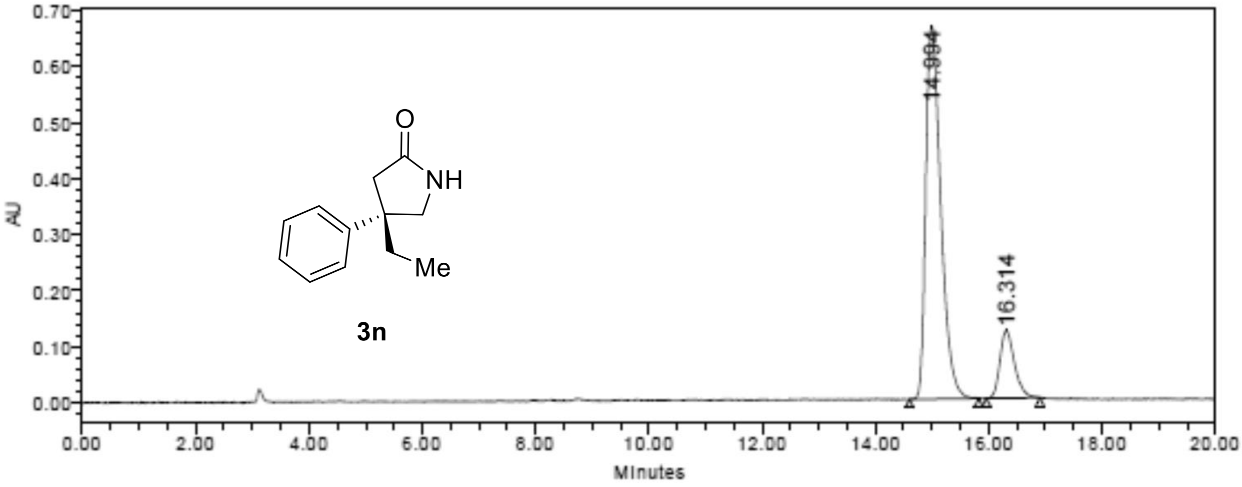

	RT	Area	% Area	Height
1	14.994	12227090	84.99	664860
2	16.314	2158879	15.01	120668

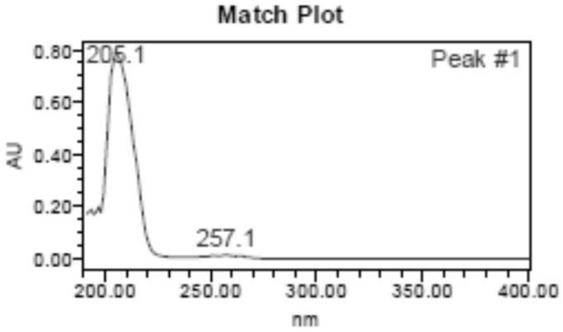

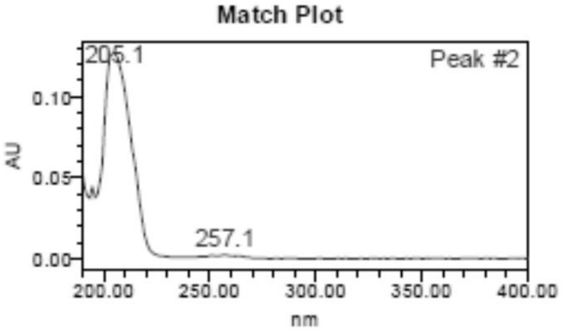

Figure SI-54. HPLC traces of compound **3n**.

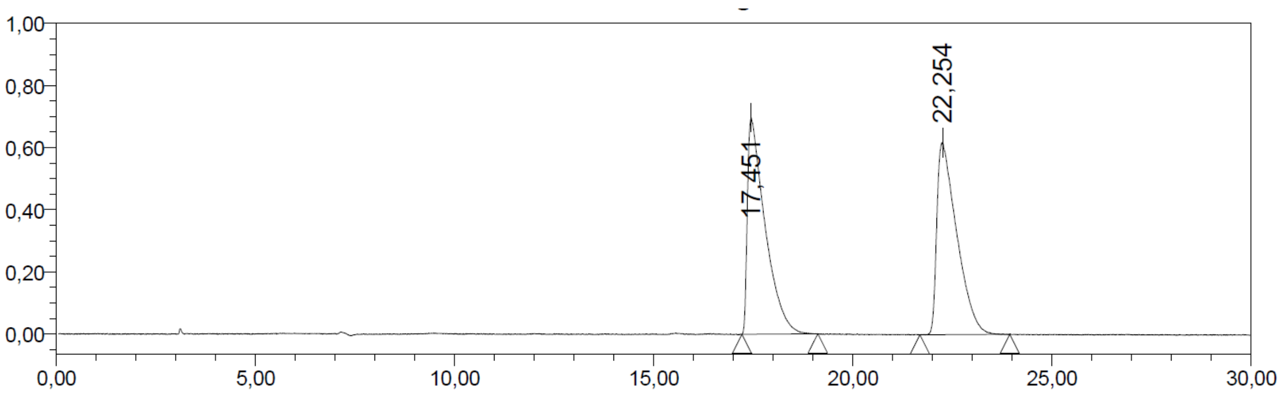

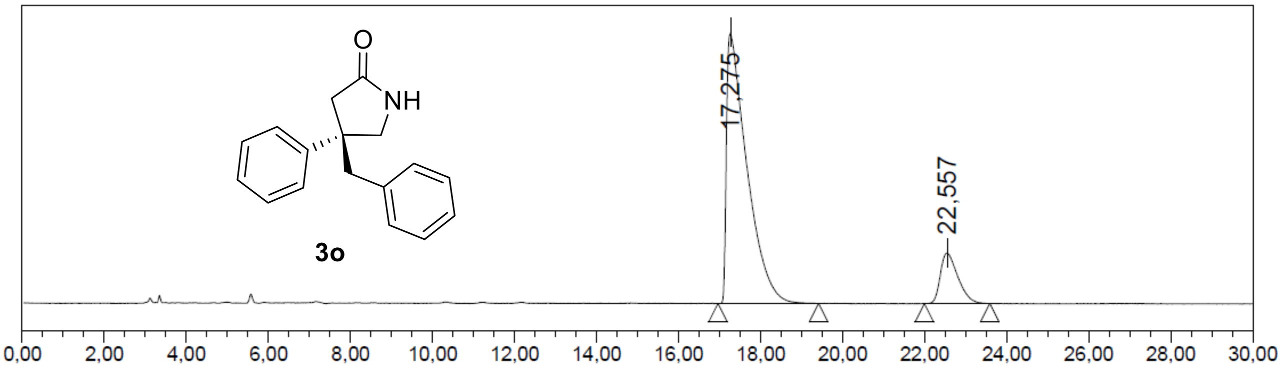

Peak Results

	Name	RT	Area	Height	% Area
1		17,275	35997624	1047788	86,15
2		22,557	5785069	195514	13,85

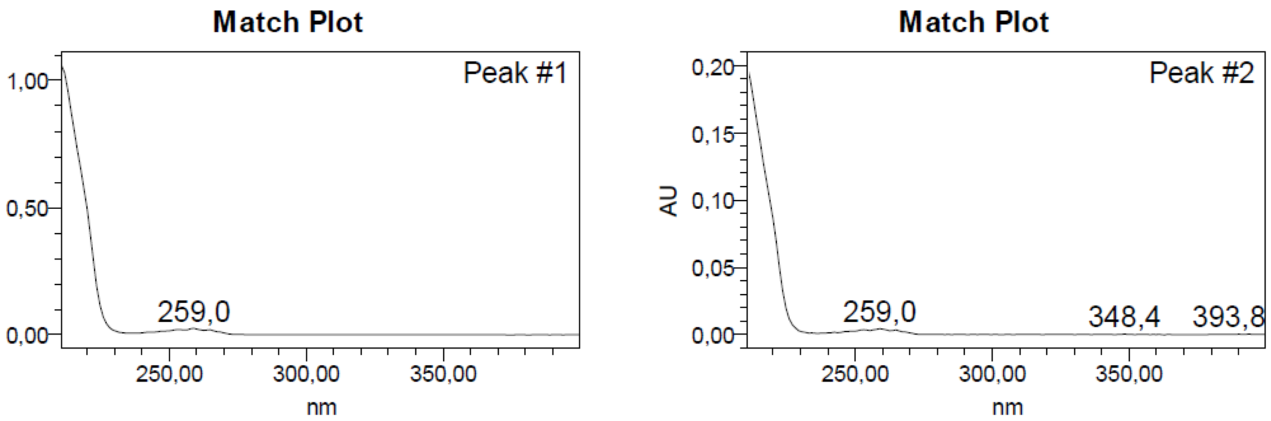

Figure SI-55. HPLC traces of compound **3o**.

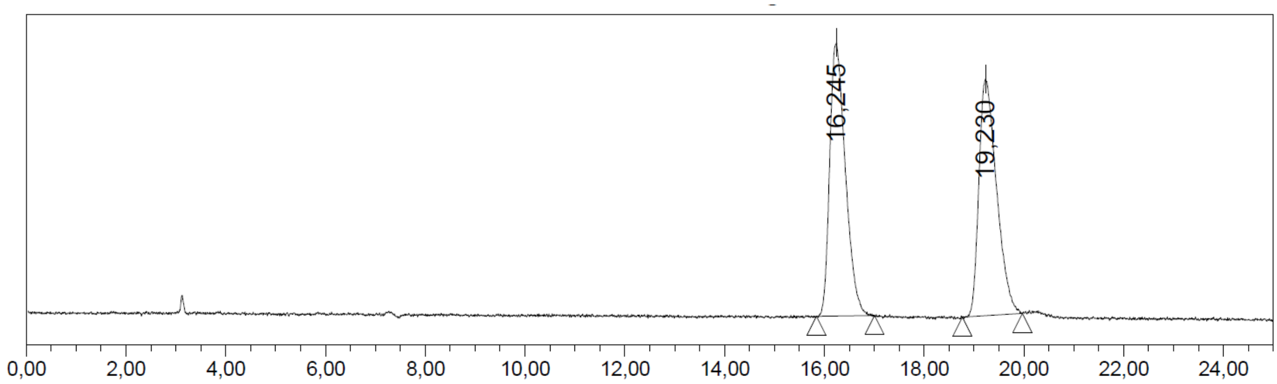

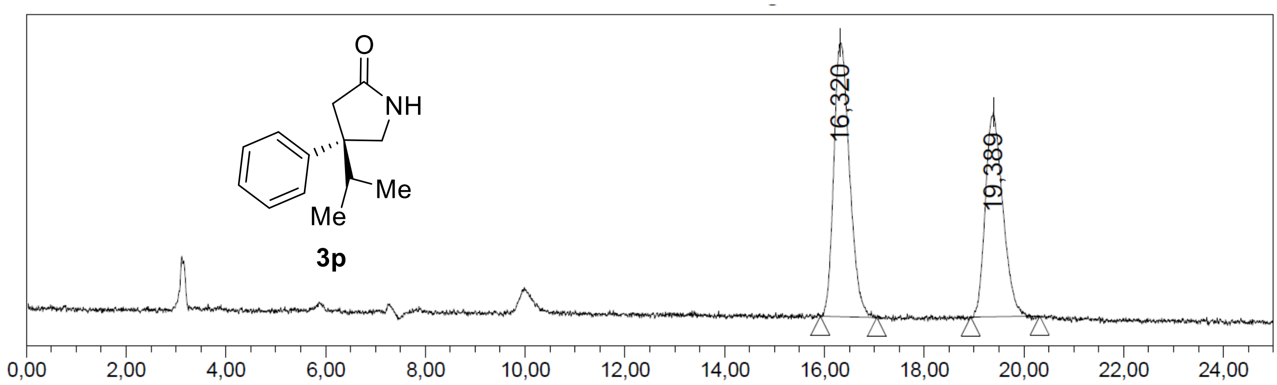

Peak Results

	Name	RT	Area	Height	% Area
1		16,245	5608047	258097	50,54
2		19,230	5489235	223075	49,46

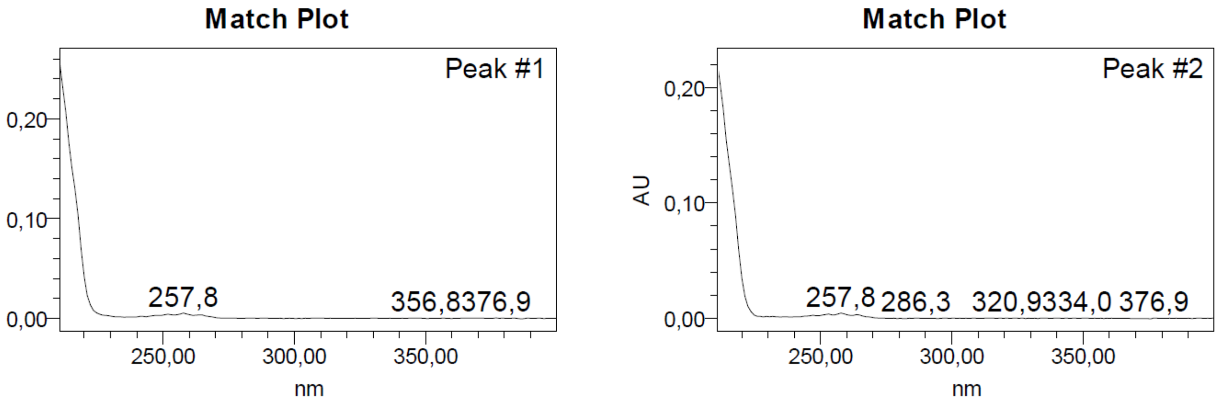

Figure SI-56. HPLC traces of compound **3p**.

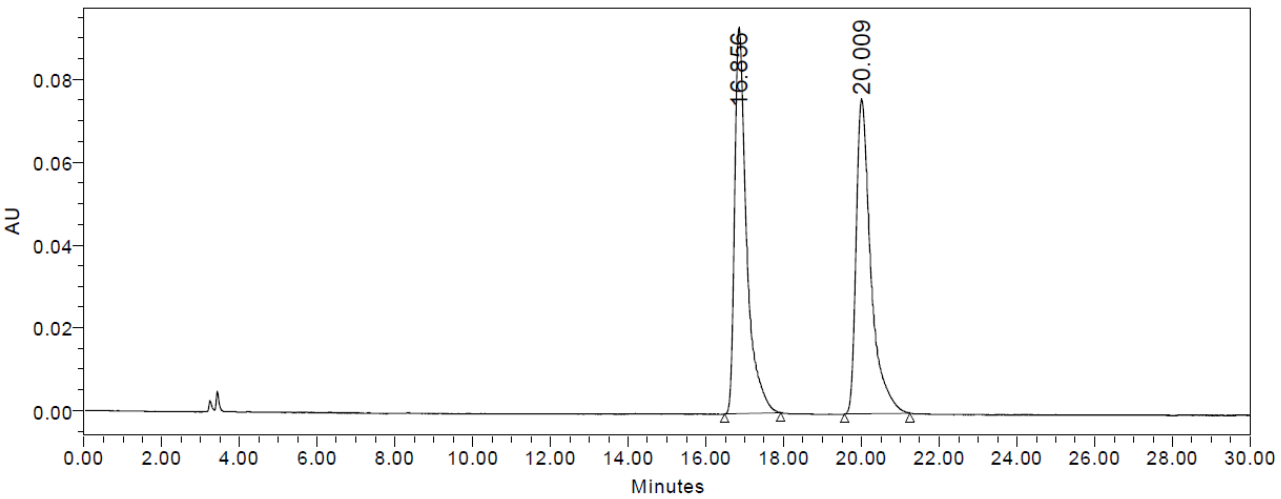

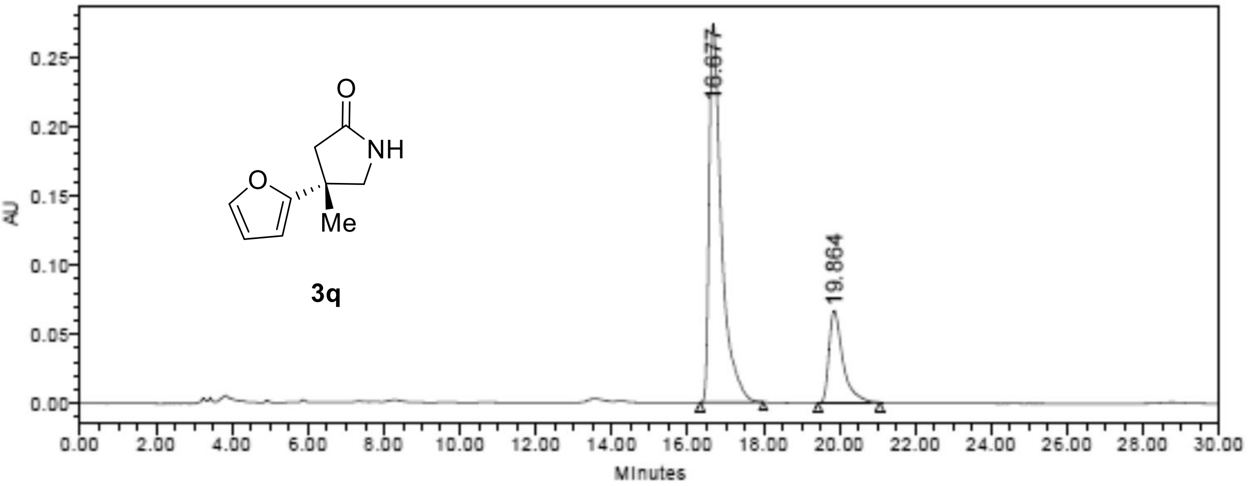

	RT	Area	% Area	Height
1	16.677	6175162	78.21	273497
2	19.864	1720463	21.79	66651

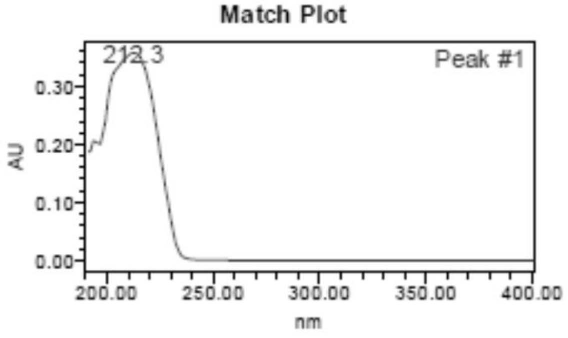

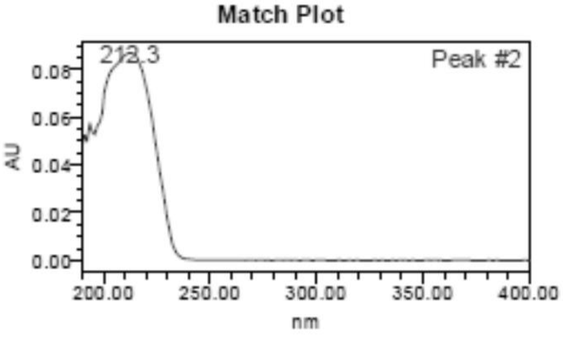

Figure SI-57. HPLC traces of compound **3q**.

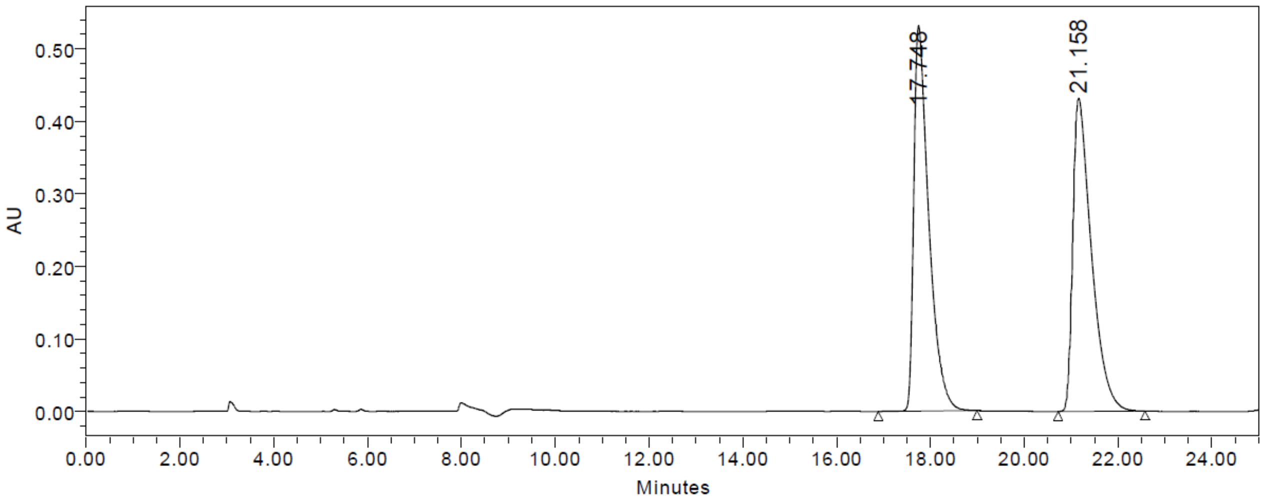

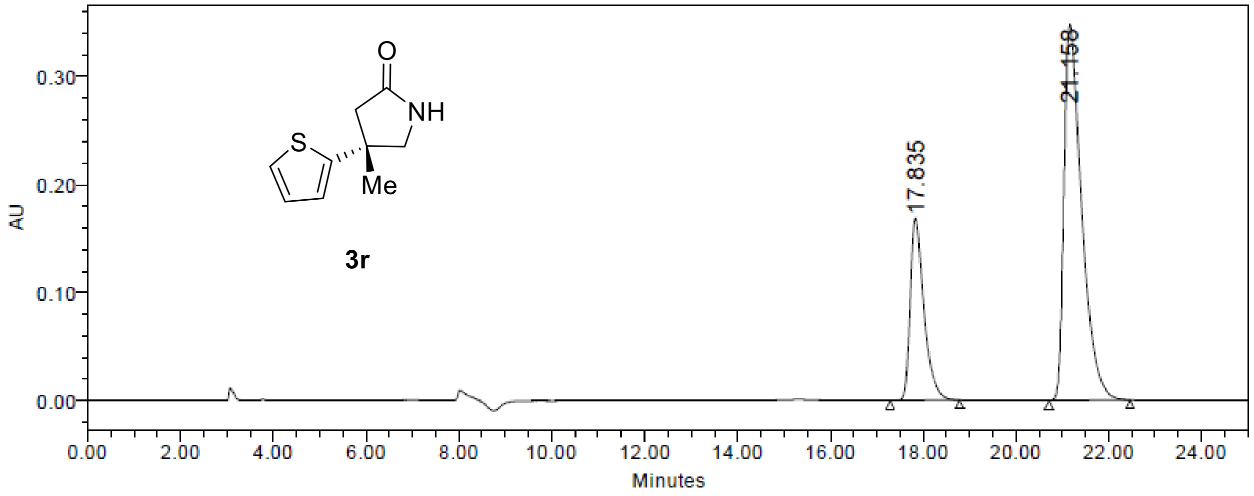

	RT	Area	% Area	Height
1	17.835	3493325	27.62	168557
2	21.158	9153213	72.38	347730

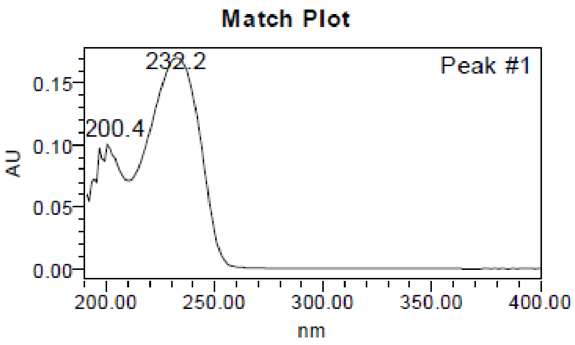

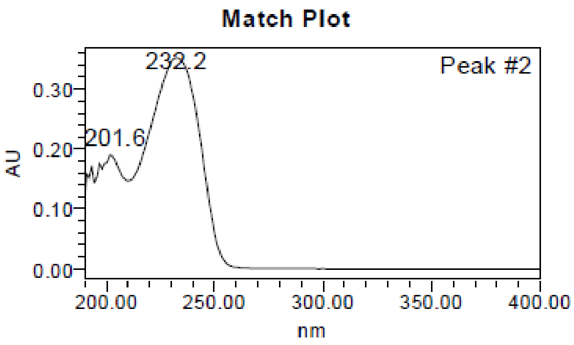

Figure SI-58. HPLC traces of compound **3r**.

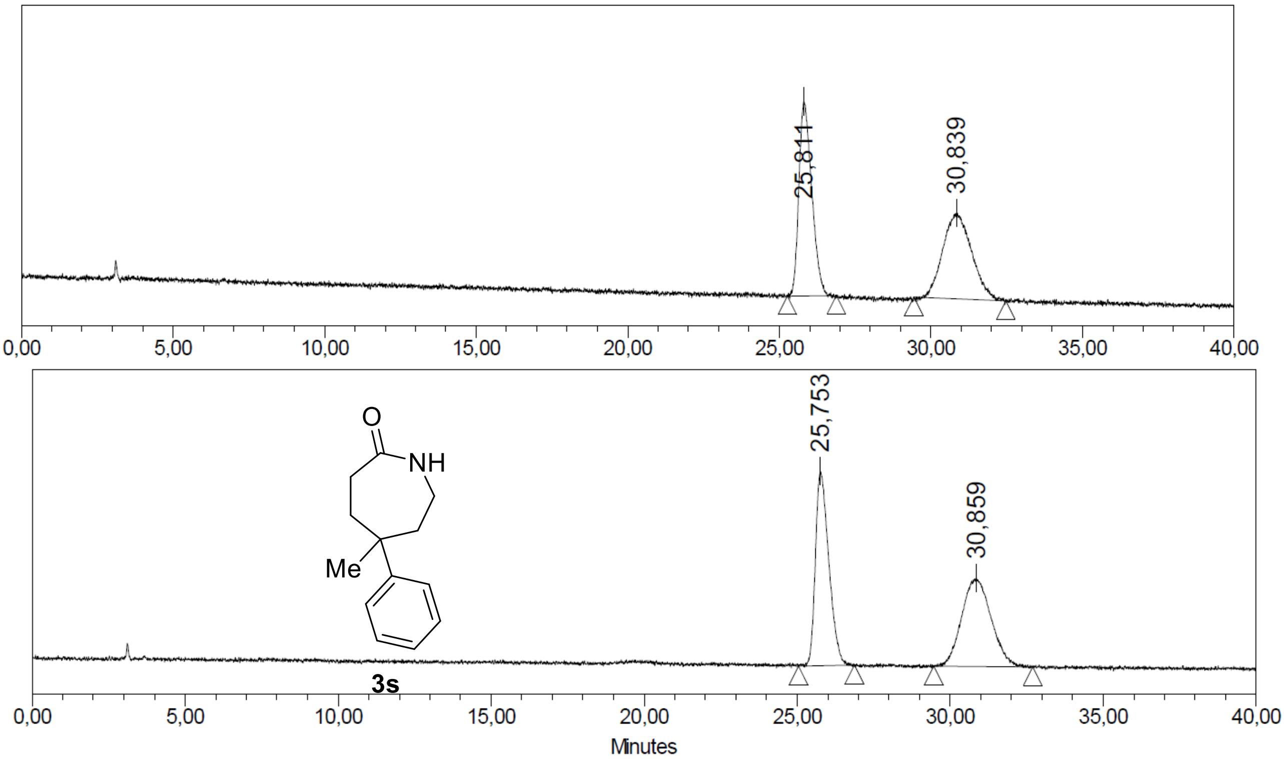

Peak Results

	Name	RT	Area	Height	% Area
1		25,753	5274564	173183	50,20
2		30,859	5232094	78637	49,80

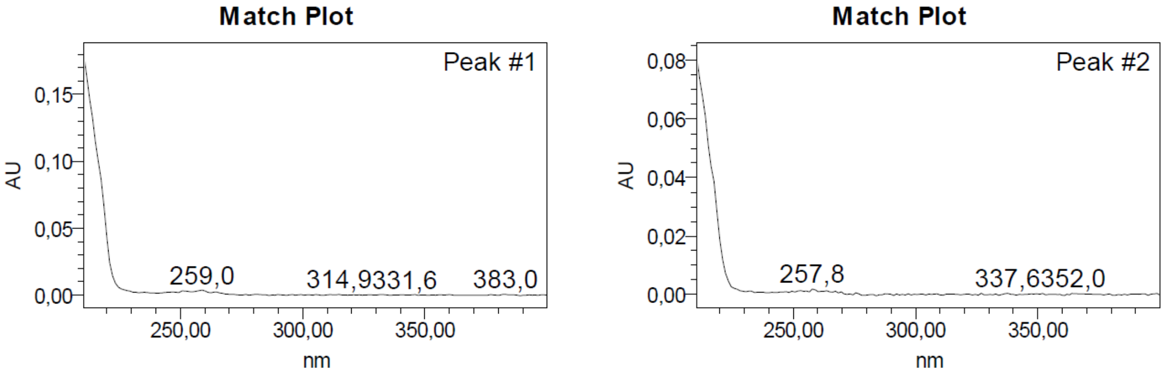

Figure SI-59. HPLC traces of compound **3s**.

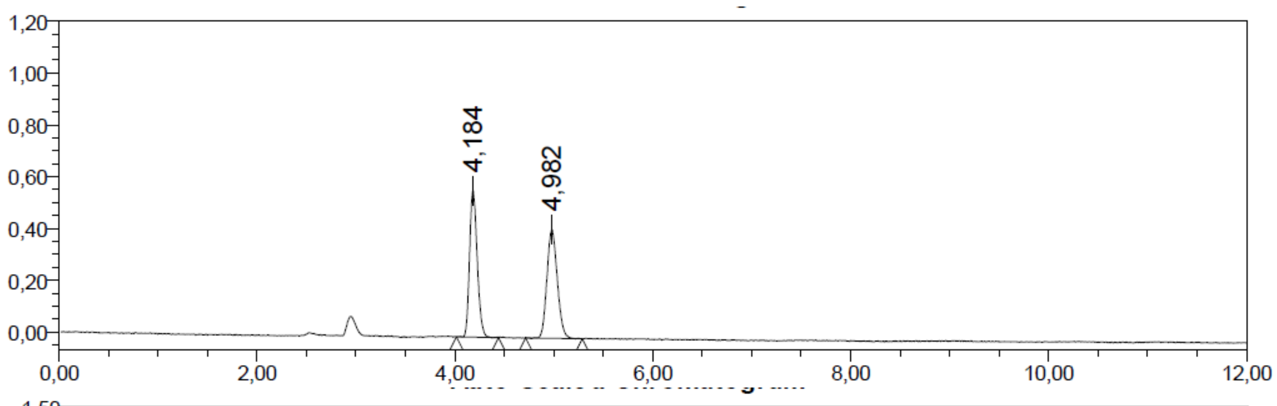

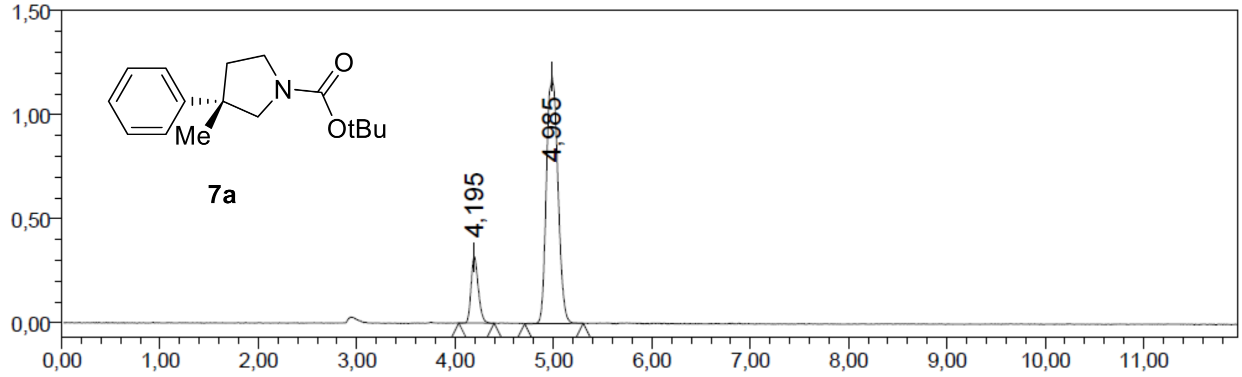

Peak Results

	Name	RT	Area	Height	% Area
1		4,195	1627890	318777	14,49
2		4,985	9607547	1188613	85,51

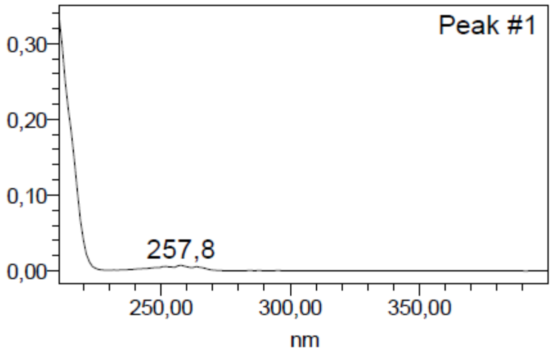

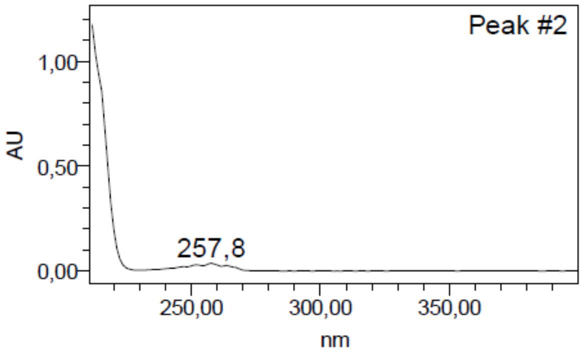

Figure SI-60. HPLC traces of compound **7a**.

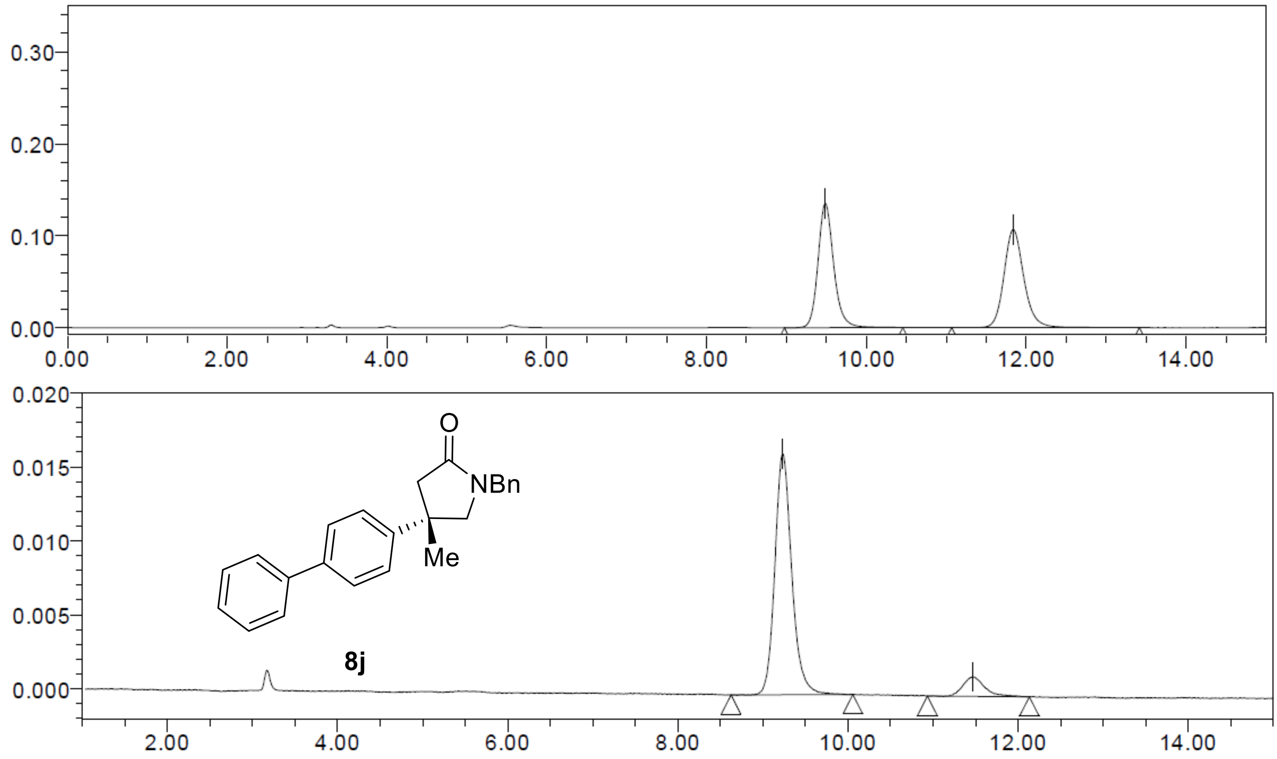

Peak Results

	RT	Area	Height	% Area
1	9.234	226235	16288	90.40
2	11.470	24024	1330	9.60

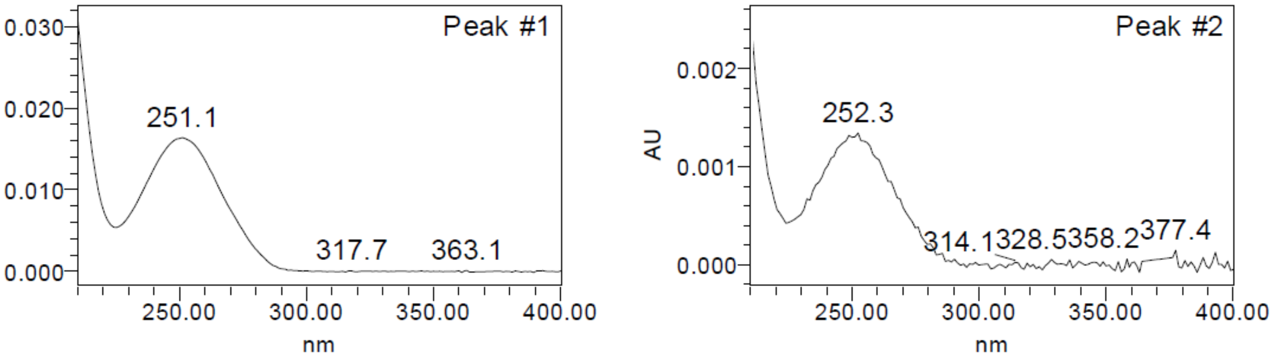

Figure SI-61. HPLC traces of compound **8j**.

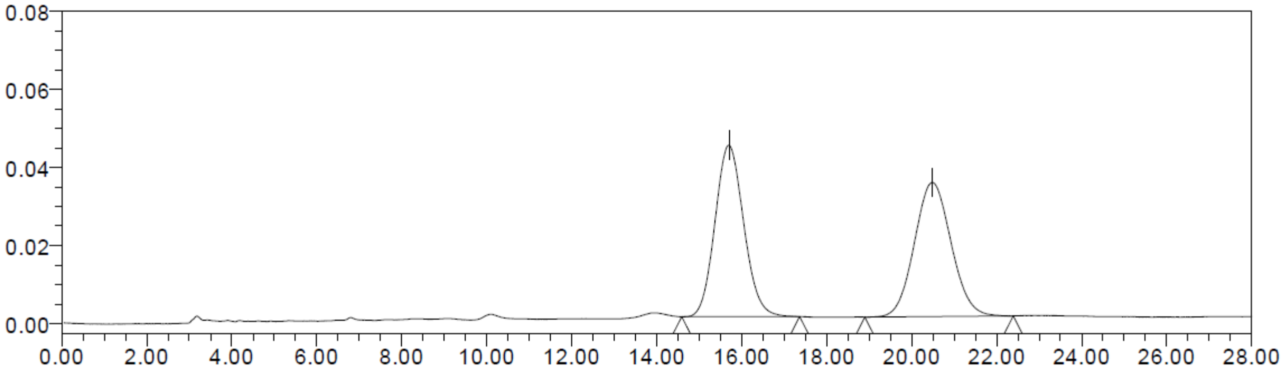

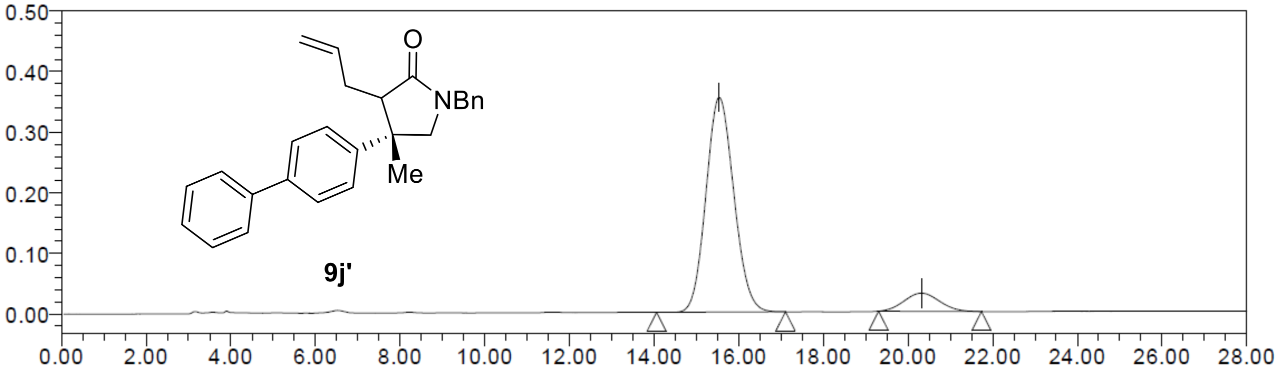

Peak Results

	RT	Area	Height	% Area
1	15.539	16221942	353913	90.35
2	20.321	1732585	29927	9.65

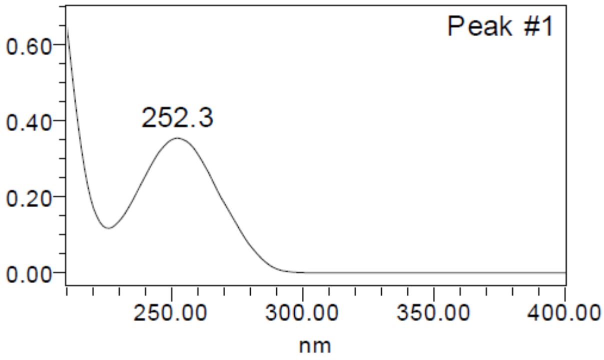

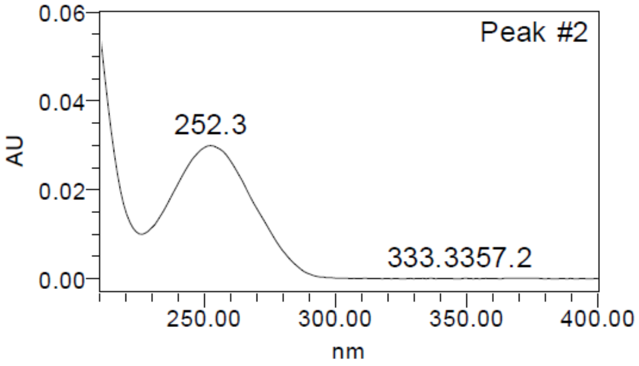

Figure SI-62. HPLC traces of compound **9j'**.

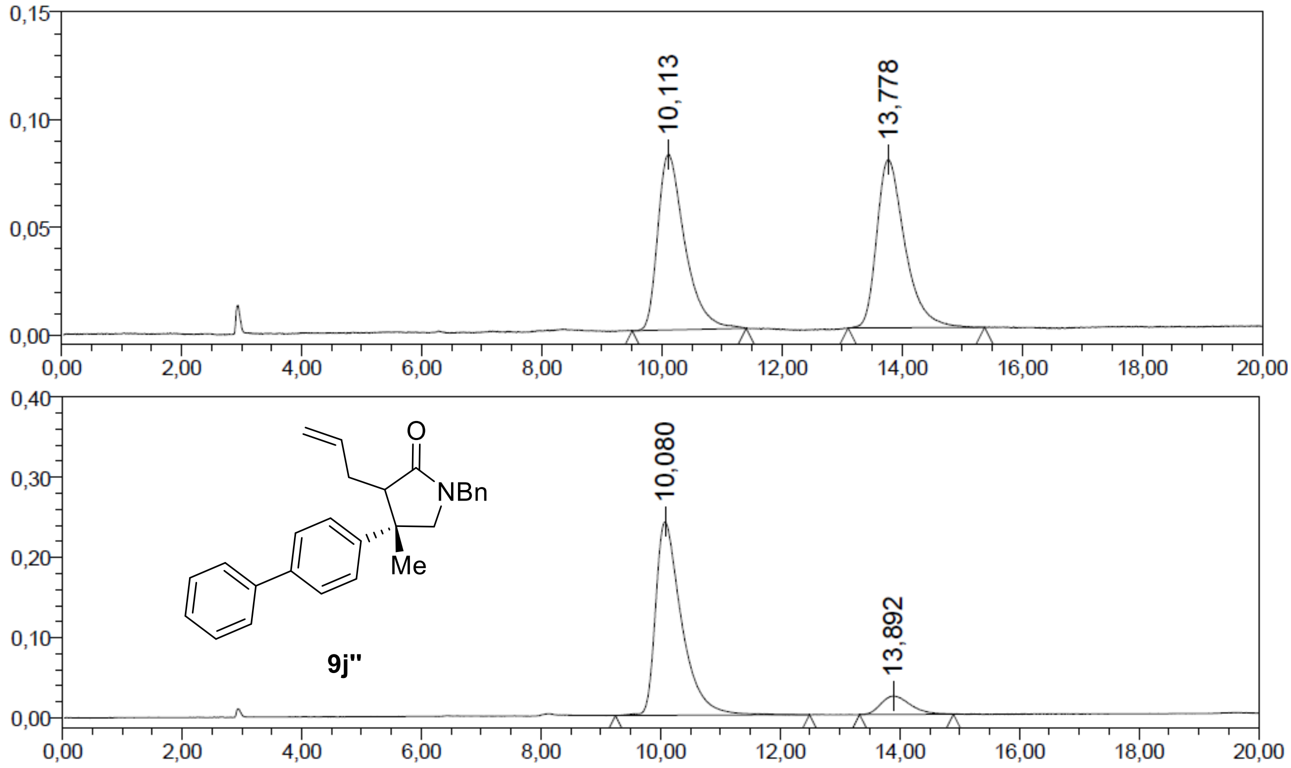

Peak Results

	Name	RT	Area	Height	% Area
1		10,080	7312627	240918	90,36
2		13,892	780397	22946	9,64

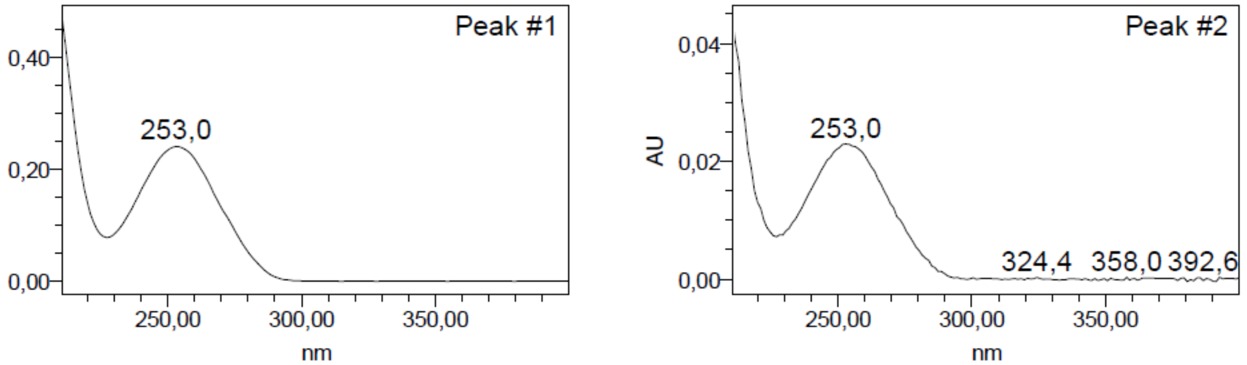

Figure SI-63. HPLC traces of compound **9j''**.
